# Carbene Complexes of Neptunium

**DOI:** 10.1021/jacs.2c02152

**Published:** 2022-05-24

**Authors:** Conrad A. P. Goodwin, Ashley J. Wooles, Jesse Murillo, Erli Lu, Josef T. Boronski, Brian L. Scott, Andrew J. Gaunt, Stephen T. Liddle

**Affiliations:** † Department of Chemistry and Centre for Radiochemistry Research, The University of Manchester, Oxford Road, Manchester M13 9PL, U.K.; ‡ Chemistry Division, Los Alamos National Laboratory, Los Alamos, New Mexico 87545, United States; § Materials Physics and Applications Division, Los Alamos National Laboratory, Los Alamos, New Mexico 87545, United States

## Abstract

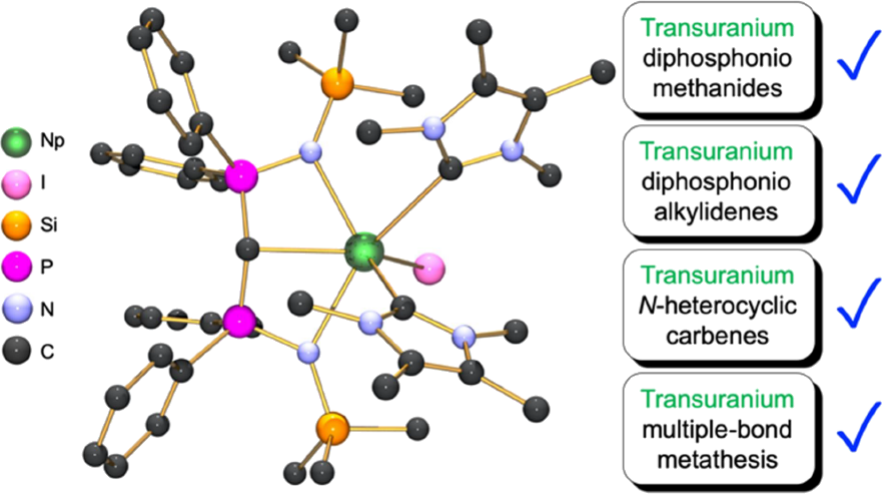

Since the advent of organotransuranium chemistry six decades ago, structurally verified complexes remain restricted to π-bonded carbocycle and σ-bonded hydrocarbyl derivatives. Thus, transuranium-carbon multiple or dative bonds are yet to be reported. Here, utilizing diphosphoniomethanide precursors we report the synthesis and characterization of transuranium-carbene derivatives, namely, diphosphonio-alkylidene- and *N*-heterocyclic carbene–neptunium(III) complexes that exhibit polarized-covalent σ^2^π^2^ multiple and dative σ^2^ single transuranium-carbon bond interactions, respectively. The reaction of [Np^III^I_3_(THF)_4_] with [Rb(BIPM^TMS^H)] (BIPM^TMS^H = {HC(PPh_2_NSiMe_3_)_2_}^1–^) affords [(BIPM^TMS^H)Np^III^(I)_2_(THF)] (**3Np**) in situ, and subsequent treatment with the *N*-heterocyclic carbene {C(NMeCMe)_2_} (I^Me4^) allows isolation of [(BIPM^TMS^H)Np^III^(I)_2_(I^Me4^)] (**4Np**). Separate treatment of in situ prepared **3Np** with benzyl potassium in 1,2-dimethoxyethane (DME) affords [(BIPM^TMS^)Np^III^(I)(DME)] (**5Np**, BIPM^TMS^ = {C(PPh_2_NSiMe_3_)_2_}^2–^). Analogously, addition of benzyl potassium and I^Me4^ to **4Np** gives [(BIPM^TMS^)Np^III^(I)(I^Me4^)_2_] (**6Np**). The synthesis of **3Np**–**6Np** was facilitated by adopting a scaled-down prechoreographed approach using cerium synthetic surrogates. The thorium(III) and uranium(III) analogues of these neptunium(III) complexes are currently unavailable, meaning that the synthesis of **4Np**–**6Np** provides an example of experimental grounding of 5f- vs 5f- and 5f- vs 4f-element bonding and reactivity comparisons being led by nonaqueous transuranium chemistry rather than thorium and uranium congeners. Computational analysis suggests that these Np^III^=C bonds are more covalent than U^III^=C, Ce^III^=C, and Pm^III^=C congeners but comparable to analogous U^IV^=C bonds in terms of bond orders and total metal contributions to the M=C bonds. A preliminary assessment of Np^III^=C reactivity has introduced multiple bond metathesis to transuranium chemistry, extending the range of known metallo-Wittig reactions to encompass actinide oxidation states III-VI.

## Introduction

The growing wealth of structurally authenticated Th and U covalent multiple bond chemistry that has been realized in recent years has redrawn the known boundaries and molecular-level comprehension of these early members of the 5f-block actinide (An) series.^1−4^ In contrast, structurally authenticated examples of molecular non-dioxo(actinyl) transuranium-element multiple bonds are limited to a high-valent Np^V^ bis(imido) complex,^5^ that is, an isolobal *N*-donor actinyl analogue, and one Np^V^ terminal mono(oxo) complex.^6^ Low-valent transuranium-element multiple bonds remain restricted to spectroscopically detected [AnE]^*n*+^ (E = O, S; *n* = 0–2) species.^7−13^ Indeed, in contrast to the dominance of lanthanide (Ln) chemistry in the trivalent state, An-ligand (L) multiple bonding is generally found for An^IV-VI^ ions. Nonetheless, with more attention given to the pursuit of transuranium-ligand multiply bonded motifs it may be possible to access An^III^=L/An^IV^=L and An^III^=L/Ln^III^=L comparisons that are currently not possible from the study of Th and U alone. The mixed-valent hexauranium ring complexes [{U^III^(BIPM^TMS^)}_3_{U^IV^(BIPM^TMS^)}_3_(μ-I)_3_(μ-η^6^:η^6^-C_6_H_5_R)_3_] ((BIPM^TMS^)^2–^ = {C(PPh_2_NSiMe_3_)_2_}^2–^, a bis(iminophosporano)methanediide; R = H, CH_3_), formally containing U^III^=C bonds represent examples of U^III^-ligand multiple bonding, but the presence of noninnocent arene bridges clouds assignments.^14^ Although organotransuranium chemistry has begun to mature over the past 5 years or so, this still sparsely populated area remains dominated by π-bonded ligands, such as the venerable cyclopentadienyl, arene, and cyclooctatetraenyl ligand sets,^15−37^ and only two σ-bonded hydrocarbyl Np complexes have been structurally validated.^38,39^

**Figure 1:**
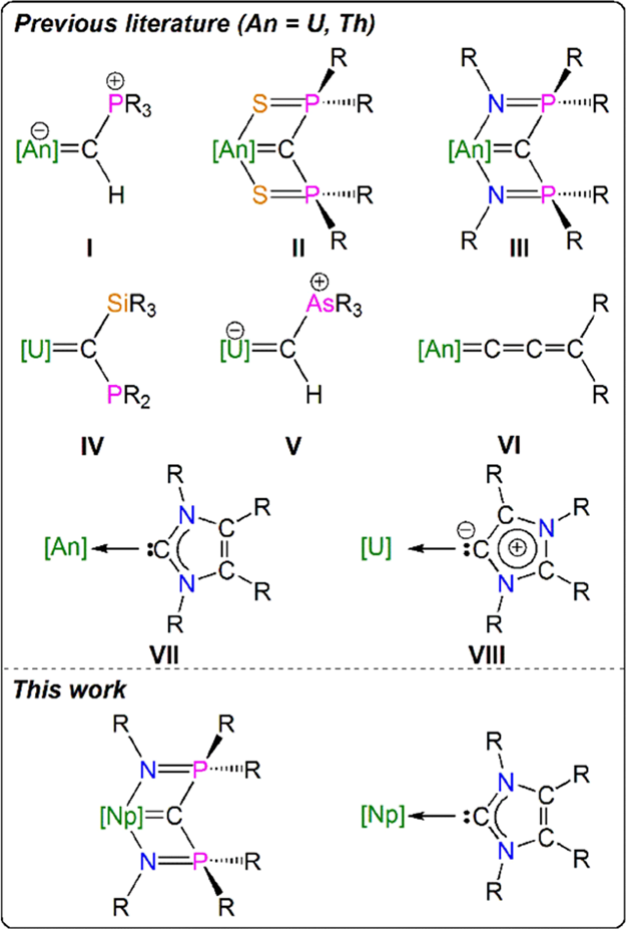
Key An=C and An ← C linkage types in early An-chemistry reported previously and in this work. Use of bracketed [An] (An = U or Th), [U], and [Np] is to acknowledge the various range of metal oxidation states and coligands that are omitted for clarity.

As fundamental species in organometallic chemistry,^40^ there is enduring interest in the chemistry of metal-carbene complexes; for example, Fischer carbenes, and of particular pertinence to this work polarized-covalent M=C double bonds, that is, alkylidenes, and dative M ← C bonds such as those from *N*-heterocyclic carbene (NHC) complexes.^40−45^ The first structurally characterized An-carbene complex was the phosphonio-alkylidene complex [U^IV^(CHPMe_2_Ph)(η^5^-C_5_H_5_)_3_] reported in 1981 (Figure 1, type **I**).^46^ Subsequently, a range of phosphonio- and diphosphonio-alkylidene complexes of U and Th have emerged (Figure 1, type **II** and **III**),^41,47,48^ and more recently, phosphino-silyl-alkylidene ({C(PPh_2_)(SiMe_3_)}^2–^),^49−51^ arsonium-alkylidene ({CHAsPh_3_}^1–^),^52^ and allenylidene ({CCCPh_2_}^2–^)^53^ derivatives have been reported (Figure 1, type **IV**-**VI**). The first An-NHC complexes (Figure 1, type **VII**), [UO_2_Cl_2_{C(NMesCX)_2_}_2_] (Mes = 2,4,6-Me_3_C_6_H_2_; X = H or Cl), were reported in 2001.^54,55^ More recently, the {C(NMeCMe)_2_} (I^Me4^) NHC has proven to be useful for supporting uranium(III)^56^ and (IV)^57,58^ and for providing comparison to mesoionic carbene derivatives (Figure 1, type **VIII**).^59^ To date, there are no transuranium-carbon multiple bonds for any transuranium oxidation state and no transuranium-NHC complexes.

Here, we report the preparation of a diphosphoniomethanide-Np complex, which contains a polarized-covalent transuranium-carbon single σ^2^-bond. This methanide complex provides an entry-point to transuranium-carbene complexes, including two diphosphonio-alkylidene-Np^III^ and two Np^III^-NHC derivatives that constitute transuranium-carbon polarized-covalent σ^2^π^2^ multiple bond and dative σ^2^ single bond interactions, respectively, Figure 1. The synthesis of these low-valent Np complexes produces clear-cut An^III^-ligand multiple bonding free of redox-active ancillary ligands and was facilitated by adopting a scaled-down prechoreographed approach using Ce as a synthetic surrogate. The analogous Th and U complexes remain experimentally unavailable, and so these Np complexes provide an instance where, instead of Th and U, it is low-valent transuranium chemistry that provides the precedent for experimentally benchmarking comparisons of homologous 5f and 4f electronic structure and bonding.

## Results and Discussion

### Synthetic Considerations

**Scheme 1:**
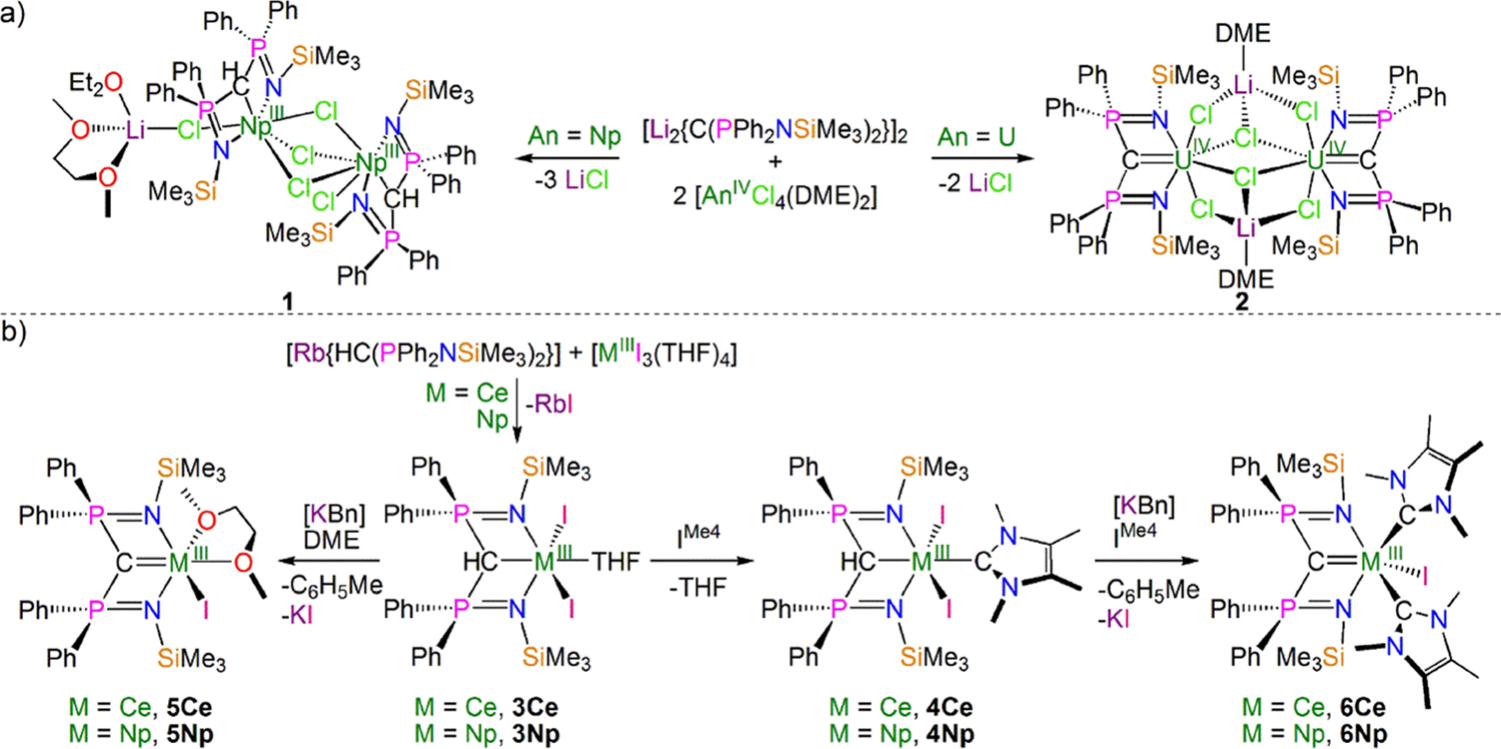
Synthesis of **1**, **2**, and **3M**–**6M** (M = Ce, Np)^a^ Complex **3Np** was not isolated. DME = 1,2-dimethoxyethane; Bn = benzyl; I^Me4^ = {C(NMeCMe)_2_}.

Previously, we found that the reaction of half an equivalent of [Li_2_{C(PPh_2_NSiMe_3_)_2_}]_2_ ([Li_2_BIPM^TMS^]_2_) with [U^IV^Cl_4_(THF)_3_] straightforwardly and reliably afforded [(BIPM^TMS^)U^IV^(Cl)(μ-Cl)_2_Li(THF)_2_] or [{(BIPM^TMS^)U^IV^(Cl)(μ-Cl)(THF)}_2_] depending on the work-up conditions employed.^60−62^ In contrast, we find that the analogous reaction between [Li_2_BIPM^TMS^]_2_ and [Np^IV^Cl_4_(DME)_2_], Scheme 1a, mostly results in intractable, dark product mixtures. However, a small crop of crystals of [(BIPM^TMS^H)Np^III^(Cl)(μ-Cl)_3_Np^III^{μ-(Cl)Li(DME)(OEt_2_)}(BIPM^TMS^H)] (**1**) was isolated on one occasion. Here, Np^IV^ has been reduced to Np^III^, and each (BIPM^TMS^)^2–^ dianion has become protonated to its (BIPM^TMS^H)^1–^ anion form ((BIPM^TMS^H)^1–^ = {HC(PPh_2_NSiMe_3_)_2_}^1–^, a bis(iminophosphorano)methanide). We note that repeating the reaction under identical conditions, except using [U^IV^Cl_4_(DME)_2_] instead of [Np^IV^Cl_4_(DME)_2_], results in the isolation of [{(BIPM^TMS^)U^IV^}_2_(μ-Cl)_6_{Li(DME)}_2_] (**2**), analogously to our earlier reports.^60−62^ In this case, U^IV^ ions are retained and no protonation of the (BIPM^TMS^)^2–^ dianion occurs, Scheme 1a. These different observations for U and Np highlight the greater redox stability of U^IV^ compared to Np^IV^,^63^ but the presence of occluded LiCl in [(BIPM^TMS^)U^IV^(Cl)(μ-Cl)_2_Li(THF)_2_] and **2** also suggested that using Li/Cl combinations could complicate reaction product outcomes. We therefore concluded that Li-reagents should be avoided and that a Np^III^ starting material could facilitate a rational route to access Np=C bonds without undesired redox chemistry.

We identified [Np^III^I_3_(THF)_4_] as a suitable starting material from which Np=C bonds could be prepared using BIPM^TMS^, given that a new, convenient Np^0^-metal-free route was recently reported to access this starting material.^64^ The combination of limited Np stocks (in comparison to Th, U, and Ln materials) coupled with the relatively high-specific radioactivity of ^237^Np, and its daughter isotopes, necessitated a small-scale use strategy (typically <30 mg Np) and preoptimized reaction and crystallization conditions based on surrogate trials. Thus, to manage the use of valuable Np we first choreographed scaled-down reactions using [Ce^III^I_3_(THF)_4_] as a synthetic surrogate for [Np^III^I_3_(THF)_4_]. The synthesis of [(BIPM^TMS^H)Ce^III^(I)_2_(THF)] (**3Ce**) using [Rb(BIPM^TMS^H)] and [Ce^III^I_3_(THF)_4_] on multigram (>10 mmol) scales has been reported previously.^65^ To determine compatibility with our Np experimental protocols, we optimized the synthesis of **3Ce** with those reagents at ∼0.04 mmol scale, Scheme 1b, and found that on this scale reactions can be performed rapidly and still yield crystalline **3Ce**. During reaction optimizations, we noted that the THF in **3Ce** is seemingly labile presenting opportunities for decomposition, which can present a major impediment on small scales. We therefore prepared the new derivative [(BIPM^TMS^H)Ce^III^(I)_2_(I^Me4^)] (**4Ce**, I^Me4^ = {C(NMeCMe)_2_}), Scheme 1b, because the I^Me4^ is a strong, kinetically inert donor and its use would pave the way to introducing NHC ligands to transuranium chemistry. With small-scale preparations of crystalline **3Ce** and **4Ce** in hand, we treated each with benzyl potassium in the presence of DME and I^Me4^,^66,67^ respectively, yielding crystalline samples of previously reported [(BIPM^TMS^)Ce^III^(I)(DME)] (**5Ce**) and the new derivative [(BIPM^TMS^)Ce^III^(I)(I^Me4^)_2_] (**6Ce**), Scheme 1b.

With the small-scale synthesis of **3Ce**–**6Ce** accomplished, we attempted the synthesis of the Np^III^ analogues, Scheme 1b. At a small scale (∼0.03–0.04 mmol of Np), utilizing [Rb(BIPM^TMS^H)] and [Np^III^I_3_(THF)_4_] we could not isolate [(BIPM^TMS^H)Np^III^(I)_2_(THF)] (**3 Np**), possibly because of the THF-lability issue observed for **3Ce**. However, adding I^Me4^ to **3Np** prepared in situ afforded [(BIPM^TMS^H)Np^III^(I)_2_(I^Me4^)] (**4 Np**) as ruby-red crystals in 16% yield. Likewise, the reaction between benzyl potassium and **3Np** (prepared in situ) in DME afforded [(BIPM^TMS^)Np^III^(I)(DME)] (**5Np**) as orange crystals in 37% isolated yield. Finally, treatment of **4Np** with benzyl potassium and I^Me4^ afforded [(BIPM^TMS^)Np^III^(I)(I^Me4^)_2_] (**6Np**) as red-purple crystals in 32% yield. Though the yields are low, which is attributed to the small scales and quite soluble nature of these complexes, they are reproducible.

Previously, it has been found that U^III^ disproportionates when paired with the (BIPM^TMS^)^2–^ dianion, requiring arene buffers in inverse-sandwich-arene complexes to stabilize this combination via extensive U-arene δ-bonding interactions.^14,61^ We therefore revisited the synthesis of the U-analogues under these new preparative conditions using [U^III^I_3_(THF)_4_], because these small-scale reactions are performed quickly. Although [(BIPM^TMS^H)U^III^(I)_2_(THF)] (**3U**) can be made and isolated as per our previous report,^14^ all attempts to deprotonate and isolate the resulting product either result in disproportionation and/or decomposition or the formation of inverse-sandwich-arene complexes, highlighting the intrinsically less stable nature of U^III^ compared to Np^III^.^68^ Nevertheless, the isolation of **4Np**–**6Np** permits opportunities to make experimentally benchmarked An^III^ vs An^IV^ and An^III^ vs Ln^III^ M=C (M = An, Ln) bonding comparisons that would otherwise remain lacking.^69−72^

### Solid-State Structures

To define the metrical details of **4Np**–**6Np**, their solid-state molecular structures were determined by single-crystal X-ray diffraction. For completeness, and as part of the choreographing scaled-down verification process, the structures of isomorphous (for each metal pair) **3Ce**–**6Ce** were determined (see the Supporting Information for full details), noting that **3Ce** and **5Ce** were structural redeterminations, whereas those of **4Ce** and **6Ce** are reported for the first time. Before we discuss the Np–C interactions in detail, we note that the Np–N distances in **4Np**–**6Np** are either statistically indistinguishable (by the 3σ-criterion) or are only marginally shorter than the corresponding Ce–N distances in **4Ce**–**6Ce**, meaning that clear-cut conclusions cannot be drawn about any M–N bond length trends in these complexes. Where the M–I distances are concerned, the Np–I and Ce–I distances vary consistently as expected for the different coordination environments of **4Np**–**6Np** and **4Ce**–**6Ce**, but we note that for each isomorphous pair the Np–I distances are consistently shorter (∼0.02–0.05 Å) than the corresponding Ce–I distances.

**Figure 2:**
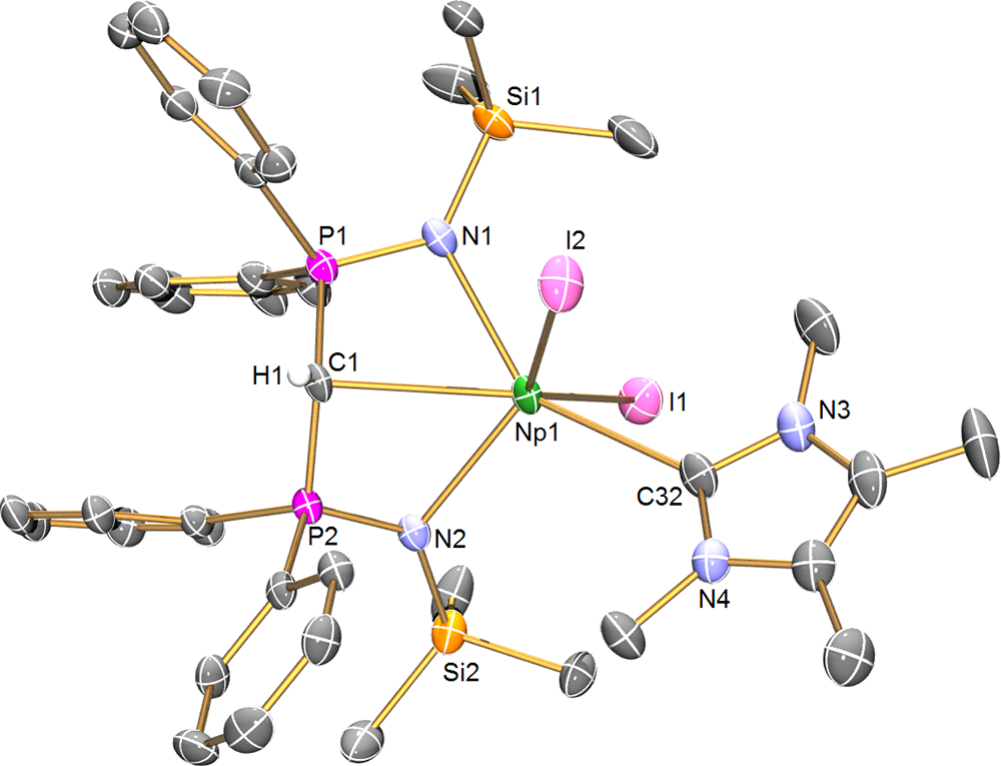
Solid-state molecular structure of complex **4Np** at 100 K. Displacement ellipsoids are set at 50% probability and nonmethanide hydrogen atoms are omitted for clarity. Selected bond lengths [Å] and angles [°]: Np1-I1 3.0727(6), Np1-I2 3.1798(6), Np1-N1 2.423(6), Np1-N2 2.458(6), Np1-C1 2.753(7), Np1-C32 2.676(8), P1-N1 1.612(6), P1-C1 1.749(7), P2-N2 1.618(6), P2-C1 1.723(7), I1-Np1-I2 135.87(2), N1-Np1-I1 93.1(2), N1-Np1-I2 84.7(12), N1-Np1-N2 105.8(2), N1-Np1-C1 63.3(2), N1-Np1-C32 144.1(2), N2-Np1-I1 91.1(2), N2-Np1-I2 131.9(2), N2-Np1-C1 62.0(2), N2-Np1-C32 110.0(2), C1-Np1-I1 133.8(2), C1-Np1-I2 83.8(2), C32-Np1-I1 88.2(2), C32-Np1-I2 70.0(2), C32-Np1-C1 134.6(2), and P1-C1-P2 128.6(4).

The structure of **4Np**, Figure 2, reveals a highly irregular six-coordinate Np ion, where the diphosphoniomethanide ligand adopts an “open-book” geometry.^48^ The two iodide ligands are approximately *trans*, though with quite an acute I-Np-I angle of 135.87(2)°, and the I^Me4^ NHC sits approximately *trans* to the methanide center, although again distorted far from the ideal (HC–Np–C_NHC_ = 134.6(2)°). The Np–CH_BIPM_ and the Np ← C_NHC_ distances are 2.753(7) and 2.678(8) Å, respectively; we note that the former is ∼0.07 Å longer than the latter, despite their respective formal anionic and neutral charge states, likely reflecting the strongly donating nature of I^Me4^ and constraints of the (BIPM^TMS^H)^1–^ anion chelate framework. The Np–CH_BIPM_ distance in **4Np** sits between the Np–C bond lengths of 2.574(4)–2.592(4) Å in [Np^III^{C_6_H_5_C(*H*)NMe_2_}_3_]^39^ and 2.831(4) and 2.838(4) Å in **1**, is slightly shorter than the Ce–CH_BIPM_ distance of 2.806(9) Å in **3Ce**, but is statistically indistinguishable (by the 3σ-criterion) from the Ce–CH_BIPM_ distance of 2.768(6) Å in isostructural **4Ce**; as expected, all are longer than the Np–C bonds (2.440(10) and 2.454(12) Å) in a previously reported Np^IV^ silylamide double cyclometallate complex.^38^ The Np–CH_BIPM_ distance in **4Np** is ∼0.08 Å shorter than the U–CH_BIPM_ distance of 2.827(3) Å in **3 U**. However, we note that the M–CH_BIPM_ distance in **3U** is ∼0.02 Å longer than the corresponding distance in **3Ce**, as expected from Shannon’s revised ionic radii (6-coordinate ions, Ce = 1.01; U = 1.03 Å)^73^ so it seems likely that the different M–CH_BIPM_ distances in **4Np** and **3U** reflect the absence of the NHC ligand in the latter rather than an underlying Np vs U difference. There are no transuranium-NHC distances with which to compare the Np–C_NHC_ distance in **4Np**, but we note that the U ← C_NHC_ distance in [U{N(SiMe_3_)_2_}_3_(I^Me4^)]^56^ is statistically indistinguishable at 2.672(5) Å. The Ce ← C_NHC_ distance of 2.731(8) Å in **4Ce** is significantly (∼0.06 Å) longer than the analogous distance in **4Np** even though according to Shannon’s revised ionic radii, six-coordinate Ce and Np are both 1.01 Å.^62^

**Figure 3:**
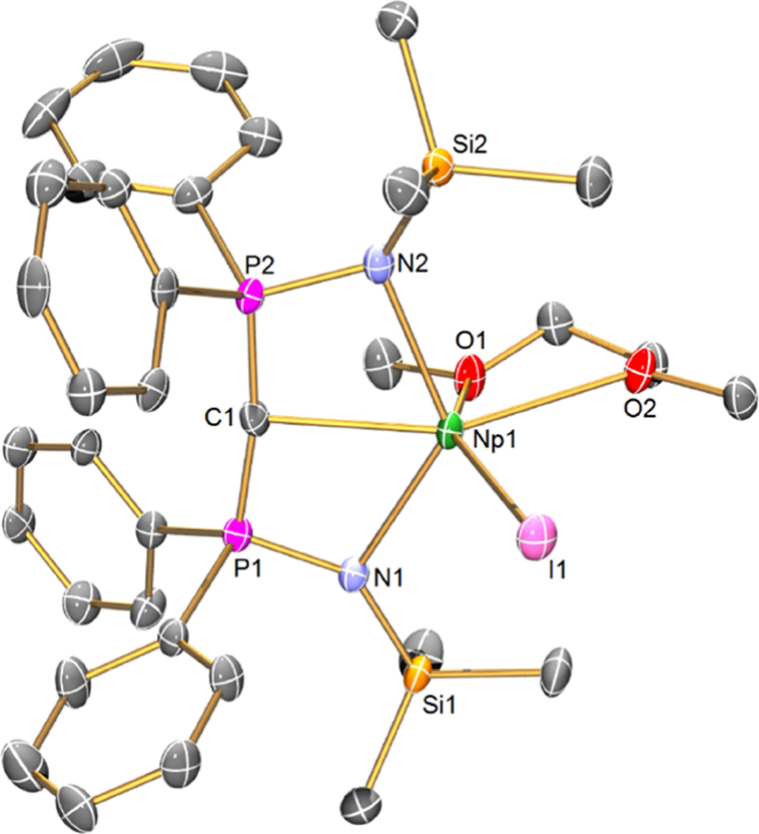
Solid-state molecular structure of complex **5Np** at 100 K. Displacement ellipsoids are set at 50% probability and hydrogen atoms and lattice solvent are omitted for clarity. Selected bond lengths [Å] and angles [°]: Np1-I1 3.1065(5), Np1-O1 2.524(5), Np1-O2 2.636(5), Np1-N1 2.431(6), Np1-N2 2.414(6), Np1-C1 2.425(7), P1-N1 1.602(6), P1-C1 1.627(7), P2-N2 1.631(6), P2-C1 1.652(7), O1-Np1-I1 153.9(2), O1-Np1-O2 61.8(2), O2-Np1-I1 95.3(2), N1-Np1-I1 103.6(2), N1-Np1-O1 81.3(2), N1-Np1-O2 122.4(2), N2-Np1-I1 95.2(2), N2-Np1-O1 101.5(2), N2-Np1-O2 102.2(2), N2-Np1-N1 128.8(2), N2-Np1-C1 65.2(2), C1-Np1-I1 109.3(2), C1-Np1-O1 95.9(2), C1-Np1-O2 152.9(2), C1-Np1-N1 63.6(2), and P1-C1-P2 170.4(5).

The structure of **5Np**, Figure 3, reveals a distorted octahedral Np ion, where the diphosphonio-alkylidene C-center is *trans* to a DME oxygen donor atom, the iodide is *trans* to the other DME oxygen donor atom, and the nitrogen donors can be considered to be *trans* to one another. Notably, the iodide is thus oriented *cis* with respect to the diphosphonio-alkylidene C-atom. The carbene adopts a T-shaped geometry, with a P-C-P angle of 170.4(5)° and a sum of angles at the C_BIPM_ center of 359.9°, which in principle orients it favorably to engage in a double-bonding interaction with Np. The Np–C_BIPM_ distance is found to be 2.425(7) Å. There is no transuranium precedent to compare the Np=C_BIPM_ distance in **5Np** to; however, we note that the Ce=C_BIPM_ distance in isostructural **5Ce** (2.477(2) Å) is longer by 0.052(2) Å. The Np=C_BIPM_ distance in **5Np** fits nicely into the trend established by previous U=C_BIPM_ complexes, with, for example, [U^VI^(BIPM^TMS^)(Cl)_2_(O)], U^VI^=C = 2.184(3) Å; [U^V^(BIPM^TMS^)(Cl)_2_(I)], U^V^=C = 2.268(10) Å; [{U^IV^(BIPM^TMS^)(μ-Cl)(Cl)(THF)}_2_], U^IV^=C = 2.322(4) Å; [{U^III^(BIPM^TMS^)}_3_{U^IV^(BIPM^TMS^)}_3_(μ-I)_3_(μ-η^6^:η^6^-C_7_H_8_)_3_], U^III^=C = 2.413(8) and 2.47(2) Å, U^IV^=C = 2.398(7) and 2.30(3) Å.^60,61^ The P–C distances in **5Np** (av. 1.640 Å) are contracted (∼0.1 Å) compared to the P–C distances in **4Np** (av. 1.736 Å), reflecting the increased charge at the central carbon atom of (BIPM^TMS^)^2–^ in **5Np** instead of (BIPM^TMS^H)^1–^ in **4Np**. This can be rationalized by invoking dipolar electrostatic shortening rather than hyperconjugation or delocalization effects.^74^

**Figure 4:**
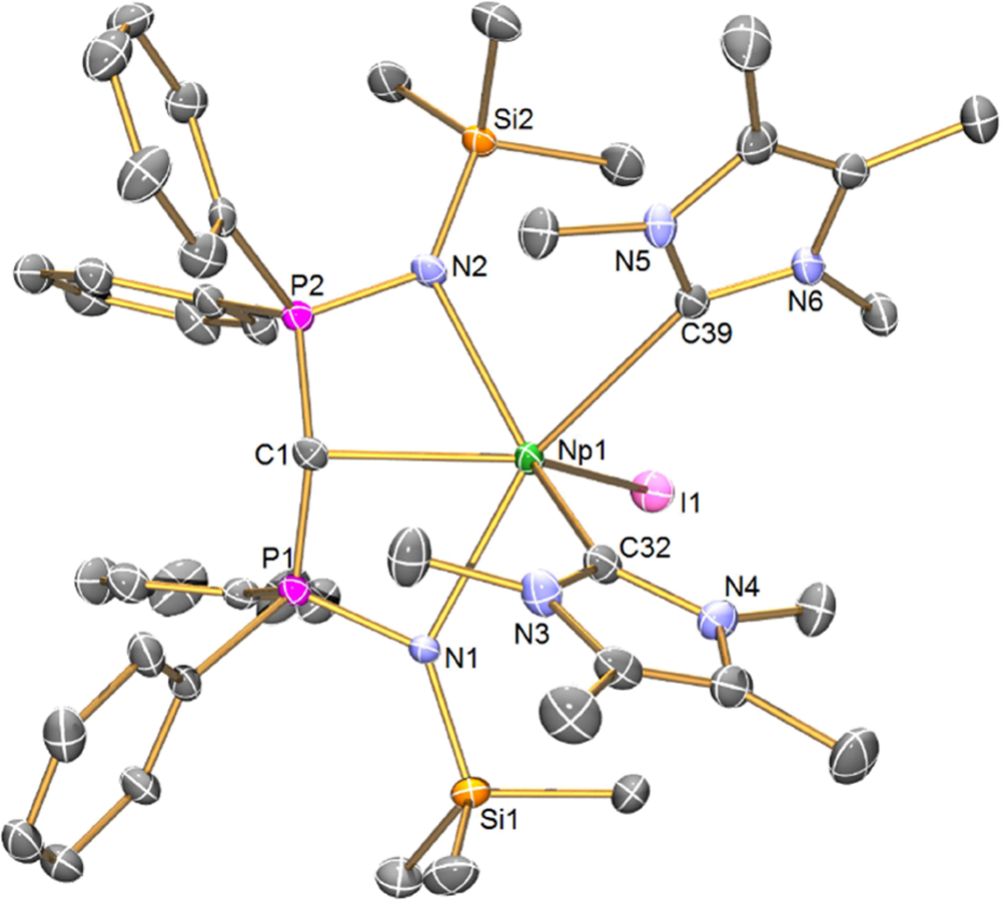
Solid-state molecular structure of complex **6Np** at 120 K. Displacement ellipsoids are set at 50% probability and hydrogen atoms and lattice solvent are omitted for clarity. Selected bond lengths [Å] and angles [°]: Np1-I1 3.1571(4), Np1-N1 2.485(4), Np1-N2 2.492(5), Np1-C1 2.490(6), Np1-C32 2.677(5), Np1-C39 2.751(6), P1-N1 1.620(5), P1-C1 1.675(6), P2-N2 1.614(5), P2-C1 1.671(5), N1-Np1-I1 91.8(2), N1-Np1-N2 120.9(2), N1-Np1-C1 64.0(2), N1-Np1-C32 81.4(2), N1-Np1-C39 154.2(2), N2-Np1-I1 98.1(2), N2-Np1-C32 132.7(2), N2-Np1-C39 81.0(2), C1-Np1-I1 127.5(2), C1-Np1-N2 64.1(2), C1-Np1-C32 98.7(2), C1-Np1-C39 123.6(2), C32-Np1-I1 124.3(2), C32-Np1-C39 73.2(2), C39-Np1-I1 98.7(2), and P1-C1-P2 136.5(3).

The structure of **6Np**, Figure 4, reveals an irregular six-coordinate Np ion, where the diphosphonio-alkylidene adopts an open book geometry,^48^ the two I^Me4^ NHCs are *cis* with respect to each other residing on the more open face presented by the (BIPM^TMS^)^2–^ ligand, and the iodide resides on the opposite, more closed face. The P-C-P angle is 136.5(3)° and the sum of angles at the C_BIPM_ center is 322.5°. This pyramidalization of the C_BIPM_ in principle would be expected to make it a poorer donor center than the one in **5Np**, which is consistent with the Np=C_BIPM_ distance of 2.490(6) Å in **6Np**, which is ∼0.07 Å longer than the corresponding Np=C_BIPM_ distance in **5Np**; the pyramidalization of the C_BIPM_ center may reflect steric congestion and also that with so many strong donors the Np ion in **6Np** may be quite electron-rich, which is consistent with the optical data (*vide infra*). Consistent with these observations, the P–C distances in **6Np** (av. 1.673 Å) are slightly (0.03 Å) longer than the P–C distances in **5Np**. Nevertheless, the Np=C_BIPM_ distance in **6Np** is slightly shorter than the corresponding Ce=C_BIPM_ distance of 2.519(2) Å in isostructural **6Ce**. The two Np ← C_NHC_ distances are 2.677(5) and 2.751(6) Å – clearly longer than the formal Np=C_BIPM_ distance, and one is statistically indistinguishable to the analogous Np ← C_NHC_ distance in **4Np**. One Np ← C_NHC_ distance is indistinguishable from the U ← C_NHC_ distance in [U{N(SiMe_3_)_2_}_3_(I^Me4^)], but the other is ∼0.07 Å longer, likely reflecting steric congestion at the Np ion. This pattern is also found in isostructural **6Ce** with Ce ← C_NHC_ distances of 2.737(3) and 2.806(2) Å, revealing that the Np ← C_NHC_ distances in **6Np** are consistently ∼0.06 Å shorter than the Ce ← C_NHC_ distances in **6Ce** despite the identical Shannon ionic radii of Np and Ce.

Overall, the solid-state molecular structures for **4Np**–**6Np** reveal that there are no substantial differences between Np^III^ and U^III^ where these Np–CH_BIPM_, Np=C_BIPM_, and Np ← C_NHC_ distances are concerned – at least across the small range of comparable molecules. However, a clear trend emerges where Np exhibits consistently shorter Np=C_BIPM_ and Np ← C_NHC_ distances (∼0.03 and ∼0.06 Å, respectively) compared to isostructural Ce=C_BIPM_ and Ce ← C_NHC_ distances. The Np–CH_BIPM_ and Ce–CH_BIPM_ distances do not exhibit any statistically significant differences. While (BIPM^TMS^)^2–^ enjoys considerable conformational flexibility, allowing facile variation in its donor strength to metals, and hence M=C distances, (BIPM^TMS^H)^1–^ is more rigid. Thus, the similarity in Np–CH_BIPM_ and Ce–CH_BIPM_ bond distances likely reflects the constraints of this chelate.

### Spectroscopic Analysis

The ^1^H nuclear magnetic resonance (NMR) spectra of **4Np**–**6Np** are consistent with their Np^III^ 5f^4^ formulations, exhibiting paramagnetically shifted resonances in spectral windows up to 67 ppm. In particular, the CH_BIPM_ resonance for **4Np** is found at −54.6 ppm. The ^31^P NMR spectra are also characteristically paramagnetically shifted, and it is notable that the ^31^P chemical shift for **4Np** (−488 ppm) shifts significantly when converted to **5Np** and **6Np** (−789 and −740 ppm), which for the latter are similar chemical shifts to [BIPM^TMS^U^IV^(X)_*n*_] complexes (X = alkyl, amide, imido; *n* = 2, 2, 1, respectively) which are 5f^2^ congeners that typically span the range −605 to −905 ppm.^75−77^

**Figure 5:**
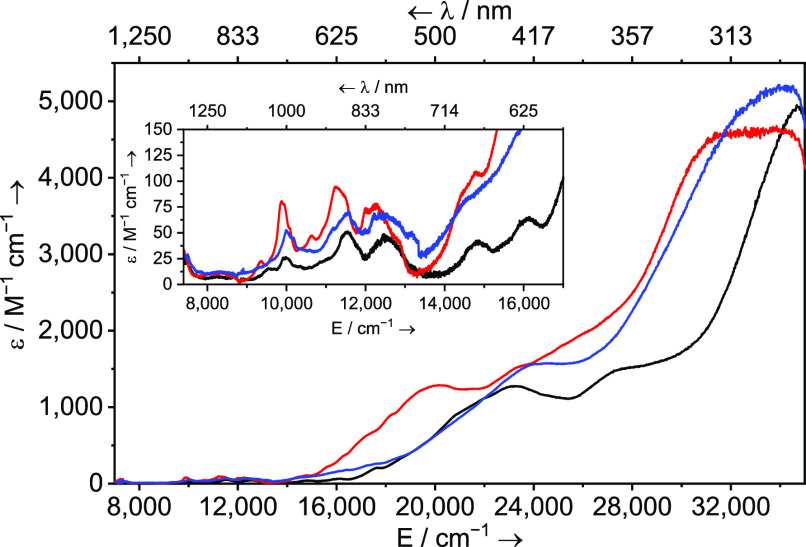
Comparison of solution UV–vis–NIR spectra of **4Np** (black line, 0.49 mM), **5Np** (blue line, 0.51 mM), and **6Np** (red line, 0.58 mM), all in toluene shown between 7000 and 35,000 cm^–1^ (1429–286 nm) at ambient temperature. Inset: Expanded view of ∼8000–16,000 cm^–1^ region.

The UV–vis–NIR spectra of **4Np**–**6Np**, Figure 5, are consistent with those of their Np^III^ formulations.^6,38,64,71,78−80^ In particular, broad pairs of absorptions, presumed to be Laporte allowed f-d transitions, are found in the 16,000–26,000 cm^–1^ region (ε = ∼1500 M^–1^ cm^–1^). As the ligand fields change from **4Np** to **5Np** to **6Np** the pairs of bands shift and can be ordered energetically as **5Np** > **4Np** > **6Np**. The most electron-rich Np would, simplistically, be expected to have the smallest f-d energy gap, and indeed, this is consistent with the ligand field at **6Np** and that the pair of f-d absorptions for **6Np** are lowest in energy of the series. While comparisons using **5Np** are complicated by it being the sole complex with DME in the coordination sphere of Np, **4Np** and **6Np** are more closely related, with BIPM, iodide, and NHC ligands common to both and here the f-d energy ordering of **5Np** > **6Np** is clear, reflecting the presence of the strongly donating diphosphonio-alkylidene in the latter. The NIR regions of the UV–vis–NIR spectra of **4Np**–**6Np**, Figure 5 inset, exhibit multiple weak absorptions assigned as Laporte forbidden f-f transitions whose overall patterns are characteristic of Np^III^.^6,38,64,71,78−80^ The absorption bands at ∼10,000, ∼11,500, and ∼12,500 cm^–1^ can be assigned to the ^5^I_7_, ^5^F_3_, and ^5^G_3_/^5^I_8_/^5^S_2_ transitions of Np^III^,^81^ and we note that the intensities and fwhm values for **5Np** (ε = ∼90 M^–1^ cm^–1^, fwhm = 530, 1000, 1108, av. 879 cm^–1^) and **6Np** (ε = ∼75 M^–1^ cm^–1^, fwhm = 426, 909, 1095, av. 810 cm^–1^) are slightly larger than those for **4Np** (ε = ∼50 M^–1^ cm^–1^, fwhm = 418, 842, 1045, av. 768 cm^–1^). Though the changes are modest, this may reflect the presence of the strong diphosphonio-alkylidene donors in the former pair compared to the diphosphoniomethanide in the latter, which in turn would invoke Np 5f-orbital contributions to the bonding of these complexes, which is indeed supported by density functional theory (DFT) calculations (*vide infra*).

### Quantum Chemical Calculations

To probe the nature of the Np–C interactions in **4Np**–**6Np**, we performed DFT calculations. Because **4Np**–**6Np** are experimentally authenticated, we calculated the experimentally inaccessible, and hence hypothetical, **4U**–**6U** complexes, using structurally validated **4Np**–**6Np** to give confidence in the DFT results and provide 5f^3^ U^III^ vs 5f^4^ Np^III^ comparisons. Given the ionic radii size match of Np^III^ and Ce^III^, and with **4Ce**–**6Ce** structurally authenticated, we also performed DFT calculations on **4Ce**–**6Ce** to provide an isostructural Ln^III^ vs An^III^ comparison. Noting that Ce^III^ is a good size match to Np^III^, but with different f-electron counts of 4f^1^ and 5f^4^, respectively, we also performed DFT calculations on hypothetical **4Pm**–**6Pm** because Pm^III^ is 4f^4^ and isoelectronic to 5f^4^ Np^III^. Confidence in the hypothetical Pm models is afforded by the structurally confirmed **4Ce**–**6Ce**. Given the good agreement between the gas-phase geometry optimized and solid-state metrical data, where comparisons are available, the DFT data can be considered to represent a reliable qualitative model of the electronic structures (Table 1) and hence a representative picture to probe bonding differences suggested by the solid-state metrical data. Note that there is little structural variance in the computed M–N bonding (Δ_max/av._ +0.04/+0.02 Å between Np and Ce structures), in line with the experimental solid-state metrical data, hence we focus discussion on the M–C bond interactions.

**Table 1:** Selected Computed Properties for **4M**–**6M** (M = Np, U, Ce, Pm)

	bond and indices		charges^d^		spin densities^e^		NBO M-C σ-bond component (%)^f^			NBO M-C π-bond component (%)^f^			QTAIM^h^	
Cmpd^a^	bond^b^	BI^c^	M	C	M	C	M^g^	C^g^	M s/p/d/f	M	C	M s/p/d/f	ρ	ε
**4Np**	Np–CH_BIPM_	0.59	1.51	–1.64	4.21	–0.01	9	91	9/0/45/46				0.04	0.05
	Np ← C_NHC_	0.83		–0.43		–0.02	0	100					0.05	0.01
**5Np**	Np=C_BIPM_	1.40	1.54	–1.96	4.36	–0.07	17	83	4/1/32/63	14	86	0/0/38/62	0.08	0.21
**6Np**	Np=C_BIPM_	1.20	1.51	–1.64	4.22	–0.05	15	85	9/1/39/51	10	90	0/1/43/56	0.08	0.18
	Np ← C_NHC_	0.65		–0.44		–0.03	0	100					0.04	0.03
	Np ← C_NHC_	0.69		–0.46		–0.03	0	100					0.05	0.03
														
**4U**	U–CH_BIPM_	0.58	1.58	–1.65	3.09	–0.01	9	91	8/0/47/45				0.04	0.05
	U ← C_NHC_	0.82		–0.45		–0.02	0	100					0.05	0.01
**5U**	U=C_BIPM_	1.28	1.57	–2.00	3.28	–0.04	14	86	4/1/42/53	13	87	0/0/40/60	0.08	0.20
**6U**	U=C_BIPM_	1.17	1.62	–1.67	3.08	–0.04	14	86	10/1/46/43	10	90	0/1/50/49	0.08	0.17
	U ← C_NHC_	0.77		–0.50		–0.03	0	100					0.05	0.03
	U ← C_NHC_	0.81		–0.48		–0.03	0	100					0.05	0.03
														
**4Ce**	Ce–CH_BIPM_	0.46	1.20	–1.54	1.04	–0.01	0	100					0.04	0.05
	Ce ← C_NHC_	0.60		–0.31		–0.01	0	100					0.04	0.01
**5Ce**	Ce=C_BIPM_	1.05	1.32	–1.82	1.07	–0.01	10	90	1/1/61/37	8	92	0/0/65/35	0.07	0.22
**6Ce**	Ce=C_BIPM_	0.96	1.29	–1.53	1.01	–0.01	9	91	7/1/65/27	7	93	2/1/60/37	0.07	0.19
	Ce ← C_NHC_	0.52		–0.38		–0.01	0	100					0.04	0.03
	Ce ← C_NHC_	0.58		–0.36		–0.01	0	100					0.04	0.03
														
**4Pm**	Pm–CH_BIPM_	0.31	1.06	–1.47	4.38	–0.04	10	90	5/0/32/63				0.04	0.05
	Pm ← C_NHC_	0.39		–0.26		–0.05	0	100					0.04	0.05
**5Pm**	Pm=C_BIPM_	0.94	1.26	–1.76	4.40	–0.02	18	82	1/0/24/75	19	81	0/0/20/80	0.07	0.16
**6Pm**	Pm=C_BIPM_	0.76	1.11	–1.48	4.39	–0.02	15	85	5/1/31/63	14	86	1/0/24/75	0.06	0.13
	Pm ← C_NHC_	0.28		–0.31		–0.02	0	100					0.03	0.05
	Pm ← C_NHC_	0.33		–0.29		–0.02	0	100					0.04	0.02

a All compounds geometry optimized without symmetry constraints at the BP86 TZP/ZORA (all-electron) level.

b M–C bond: M–CH_BIPM_ = methanide of (BIPM^TMS^H)^1–^; M ← C_NHC_ = I^Me4^ NHC carbene; M=C_BIPM_ = diphosphonio-alkylidene of (BIPM^TMS^)^2–^.

c Nalewajski–Mrozek bond indices.

d MDC_*q*_ charges.

e MDC_*m*_ spin densities.

f Natural bond orbital (NBO) analysis.

g Values of 0% for the total M contribution to the M–C bond mean that the M contribution is below the cut-off threshold of NBO (5%).

h Quantum Theory of Atoms in Molecules (QTAIM) bond critical point topological electron density (ρ) and ellipticity (ε) analysis.

**Figure 6:**
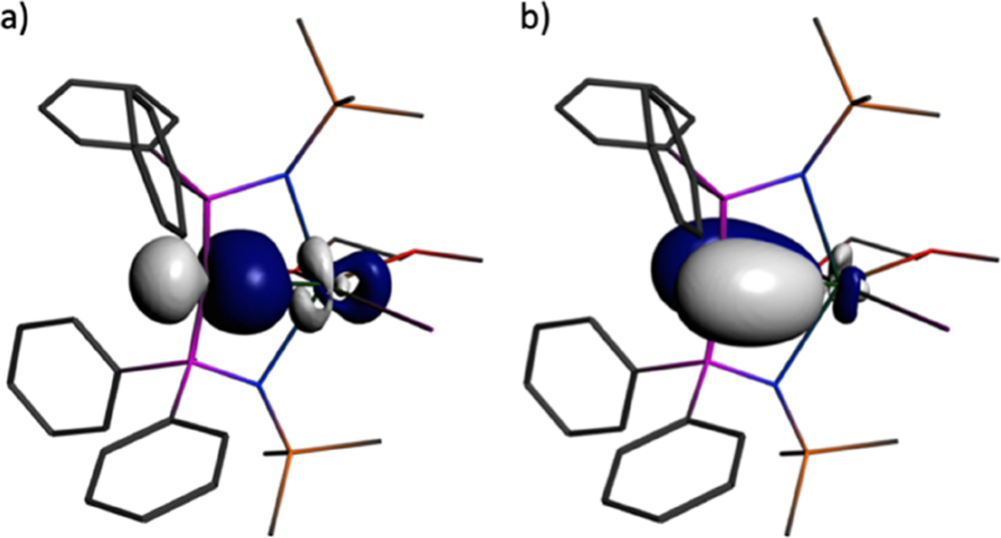
NBO representations of the Np=C_BIPM_ σ- and π-bond interaction in **5Np**. (a) Np=C_BIPM_ σ-bond. (b) Np=C_BIPM_ π-bond. Hydrogen atoms are omitted for clarity.

As expected, the Np–CH_BIPM_ bond order in **4Np** (0.59) reveals a polarized linkage, but conversion of that linkage to Np=C_BIPM_ gives bond indices for **5Np** and **6Np** (1.40 and 1.20) that are at least double that of **4Np**, reflecting their formal polarized-covalent single- and double-bond natures, respectively. The dative Np ← C_NHC_ bond orders are broadly similar to the covalent Np–CH_BIPM_ bond index, reflecting the strong donor nature of I^Me4^, but this metric is noticeably larger for the Np ← C_NHC_ bond in **4Np** (0.83) than **6Np** (av. 0.67) reflecting the weaker donor strength of (BIPM^TMS^H)^1–^ compared to (BIPM^TMS^)^2–^. The computed charges and spin densities are overall consistent with Np^III^ ions (av. 1.52, 4.26, respectively) and charge donation from the ligands to Np centers. Highly electrostatic Np–CH_BIPM_ and Np ← C_NHC_ interactions are returned by NBO analyses, but polarized-covalent Np=C_BIPM_ twofold bonding interactions with Np contributions of 10–17% are confirmed, as exemplified by the Np=C_BIPM_ bond of **5Np**, Figure 6 and Table 1 (and see the Supporting Information). QTAIM analysis of **4Np**–**6Np** confirms the anticipated polarized-covalent nature of the Np=C_BIPM_ bonds in **5Np** and **6Np**, again with more covalent Np=C_BIPM_ than Np ← C_NHC_ bonds. The bond critical point ellipticity values reveal cylindrical single bonds for the Np–CH_BIPM_ and Np ← C_NHC_ interactions (ε values close to zero) and asymmetric double-bond interactions for the Np=C_BIPM_ linkages (ε values that deviate substantially from zero, for example, benzene and ethene have ε values of 0.23 and 0.45, respectively).^82^

Previous work on U^IV^=C_BIPM_ complexes, using the same level of theory for the calculations on **5Np** and **6Np** (BP86, all-electron ZORA TZP), found total U^IV^ contributions averaging ∼15–18%, with bond orders of ∼1.4. Those data are remarkably similar to those found for Np^III^**5Np** and **6Np**. To account for this, two competing effects merit consideration. For equivalent oxidation states, Np has a greater effective nuclear charge than U, so the radial distribution of the 5f- and 6d-orbitals will be smaller for Np than U. However, as the oxidation state is decreased, the 5f- and 6d-orbitals will in principle expand. Therefore, we tentatively suggest when considering Np^III^ in relation to U^III^, and then U^IV^, that 5f- and 6d-orbital contraction from increasing *Z*_eff_ between equivalent oxidation states may be offset when moving to U with an oxidation state one unit higher (at least in the context of these An=C_BIPM_ bonding interactions). The result would be that, due to net equalization of these competing effects, Np^III^ in the An=C_BIPM_ ligand field is roughly equivalent to U^IV^ with a result that total Np contributions to the Np^III^=C_BIPM_ bonds in **5Np** and **6Np** appears to be similar to U^IV^=C_BIPM_ congeners, noting that the 5f orbital character dominates overall. However, the Np^III^=C_BIPM_ σ- and π-bonds of **5Np** and **6Np** (av. 58% 5f character) exhibit a considerably less 5f character than U^IV^=C_BIPM_ complexes (∼70–90% 5f character), which is counterbalanced for Np by increased 7s and 6d orbital participation for the σ-bonds and mainly 6d orbital participation for the π-bonds. An additional difference is the consistently significant 7s contributions (4–9%) from Np to the σ-components of the Np^III^=C_BIPM_ bonds which has not been found for U^IV^=C_BIPM_ bonds.

The above analysis provides a baseline from which Np^3+^-containing **4Np**–**6Np** is compared to hypothetical U^3+^-containing **4U**–**6U** congeners with confidence. The M–CH_BIPM_ bond orders for **4Np** and **4U** are very similar (∼0.59), with **5Np** having a higher bond order (1.40) than **5U** (1.28), but the opposite is found for **6U** (1.17) vs **6Np** (1.20). The NBO data reveal that the U ← C_NHC_ bonds are largely electrostatic and invariant, which is the same as the Np ← C_NHC_ bonds. The U^III^=C_BIPM_ bonds are similar to the Np^III^=C_BIPM_ bonds but slightly more polarized in terms of total metal contributions (9–14 vs 9–17%, respectively). While the π-bonds are little changed from Np^III^ to U^III^, still having dominant 5f character (but reduced compared to U^IV^=C_BIPM_ complexes), the σ-bonds for U have approximately equal 6d vs 5f orbital contributions but, as was found for Np, significant 7s contributions (4–10%) are revealed. Thus, it would seem that An^III^=C_BIPM_ bonds for both An = U and Np consistently exhibit significant (∼4–10%) 7s contributions that are not present in U^IV^=C_BIPM_ complexes. We note that the QTAIM data for **4U**–**6U** are quite similar to those of **4Np**–**6Np** and confirm the presence of U^III^=C_BIPM_ double-bond interactions. Previously, we extrapolated bond orders and U% contributions to the U^III^=C_BIPM_ bonds in the mixed-valent [{U^III^(BIPM^TMS^)}_3_{U^IV^(BIPM^TMS^)}_3_(μ-I)_3_(μ-η^6^:η^6^-C_7_H_8_)_3_] molecule of ∼1.2 and 13%, respectively.^14^ These data certainly fit well into the trends of U over oxidation states of III-VI (U^III^, ∼13; U^IV^, ∼18; U^V^, ∼26; U^VI^, ∼28%) and the data in Table 1. However, the presence of arene bridges and the large number of basis functions for [{U^III^(BIPM^TMS^)}_3_{U^IV^(BIPM^TMS^)}_3_(μ-I)_3_(μ-η^6^:η^6^-C_7_H_8_)_3_] meant that these were extrapolations at best. It is thus notable that those values compare very well to the computed values for **4U**–**6U** (9, 14, and 14% respectively), which has been enabled with confidence by being benchmarked against **4Np**–**6Np**, demonstrating the value of accessing transuranium targets when U congeners are experimentally unavailable.

Next, we turn our attention to comparing the data for **4Np**–**6Np** to **4Ce**–**6Ce** because both are experimentally validated series of complexes containing central metal ions with identical Shannon ionic radii. We noted in the analysis of structural data above that the M–CH_BIPM_ bond distances are largely the same for **4Np** vs **4Ce**, but the M=C_BIPM_ and M ← C_NHC_ bonds tended to be shorter in **5Np** and **6Np** vs **5Ce** and **6Ce**. In line with those data, the computed bond metrics largely follow the same pattern, resulting in lower bond orders on a like-for-like basis for Ce (Ce–C_BIPM_ 0.46, Ce=C_BIPM_ av. 1.00, Ce ← C_NHC_ av. 0.57) vs Np (Np–C_BIPM_ 0.59, Np=C_BIPM_ av. 1.30, Np ← C_NHC_ av. 0.72). This pattern translates through to the NBO analysis, where for each system the Ce contributions (7–10%) to given polarized-covalent bonds are about 60% of the corresponding Np values (9–17%). The Ce=C_BIPM_ σ- and π-bonding components are dominated by 5d character (av. 63%), with an approximate 2:1 ratio of 5d:4f character. Also, analogously to Np, 6s character is found (1–7%) for the Ce=C_BIPM_ σ-bonds, which is significant but slightly lower than the corresponding Np 7s contributions to the Np=C_BIPM_ bonds (4–9%).

Noting the clear differences between the computed electronic structures of 5f^4^**4Np**–**6Np** and 4f^1^**4Ce**–**6Ce**, we finally compare isoelectronic 5f^4^**4Np**–**6Np** vs 4f^4^**4Pm**–**6Pm**, where the experimentally anchored calculations on **4Ce**–**6Ce** provide confidence in extending the models and computational methods to **4Pm**–**6Pm**. Notably, the computed Pm–C distances are, like-for-like, always slightly longer for **4Pm**–**6Pm** vs **4Np**–**6Np**, but, as anticipated, the Pm–C distances are shorter than the corresponding Ce–C distances because of the increased effective nuclear charge and lanthanide contraction. However, while the Np–C bond orders (0.59–1.40) are like-for-like larger than the Pm–C bond orders (0.28–0.94), the latter are also consistently lower than the corresponding Ce–C bond orders (0.46–1.05). Inspection of the NBO data of **4Pm**–**6Pm** reveals two notable points. First, the Pm% contributions to the Pm–CH_BIPM_ (10%) and Pm=C_BIPM_ (14–19%) bonds of **4Pm**–**6Pm** are similar to the Np% of the Np–CH_BIPM_ (9%) and Np=C_BIPM_ (10–17%) bonds of **4Np**–**6Np** and are thus significantly larger than the corresponding Ce data (7–10%). Second, whereas the Ce bonding is dominated by 5d character (av. 63%), for **4Pm**–**6Pm** the bonding is dominated by 4f character (av. 71%), more than the 5f character of the Np congeners (av. 56%), along with 6s contributions (1–5%). The high Pm% contributions to the bonding but low bond orders at first sight may seem contradictory, but we suggest that this is an example of 4f mixing due to a good energy match with the C_BIPM_ orbitals but poor spatial overlap.^83,84^ Though the differences are small, this would appear to be the case based on the QTAIM data, where the Pm values are consistently smaller than the Np values.

The computational results can be summarized as follows: (i) decreased f orbital contributions to bonding with M^III^ ions (relative to M^IV^ ions) can be compensated for by s and d contributions; (ii) the Np^III^=C_BIPM_ systems are overall comparable to U^IV^=C_BIPM_ analogues; (iii) Np is the most covalent (by total metal% contribution to the bonding) of Np, U, Ce, and Pm for these M^III^=C_BIPM_ complexes; (iv) the bonding of the Ce complexes is dominated by 5d character; (v) the bonding of the Pm complexes is dominated by 4f character, but here the covalency is likely due to good energy matching and not spatial overlap; (vi) the M–C_NHC_ bonding is consistently highly electrostatic for all complexes; and (vii) by isolating and characterizing Np^III^=C_BIPM_ complexes it has been possible to complete benchmarking of U contributions in U=C_BIPM_ bonding over U oxidation states of III-VI.

### Preliminary Reactivity Assessment

**Scheme 2:**
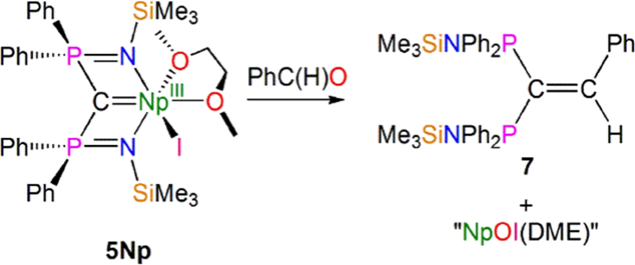
Reaction of **5Np** with Benzaldehyde to Produce the Alkene **7**

A preliminary reactivity study of **5Np** reveals metallo-Wittig reactivity, as anticipated for a species with a formal Np=C double-bond interaction.^47,62,85^ In particular, benzaldehyde reacts with **5Np** to afford the alkene product PhC(H)=C(PPh_2_NSiMe_3_)_2_ (**7**), Scheme 2, as evidenced by ^1^H and ^31^P NMR spectroscopic data.^62^ As far as we are aware, the reaction of **5Np** with benzaldehyde constitutes the first multiple bond metathesis reaction in transuranium chemistry, and indeed An^III^-chemistry more broadly given the dearth of An^III^-ligand multiple bonding. The metallo-Wittig reactivity of **5Np** is complementary to the metallo-Wittig reactivity already established for U-analogues in oxidation states IV-VI,^47,62,85^ showing now that An=C double bonds can execute multiple bond metathesis over the full range of commonly accessible An oxidation states (III-VI). The reactivity of **5Np** also forges a link to the metallo-Wittig reactivity of Ln^III^=C bonds, providing a bridge between trivalent M=C bond metathesis chemistry of Ln and An ions.

## Conclusions

To conclude, we have reported the synthesis and characterization of transuranium-carbon polarized-covalent σ^2^π^2^ multiple and dative σ^2^ single bond interactions. The new diphosphoniomethanide-, diphosphonio-alkylidene-, and *N*-heterocyclic carbene-neptunium(III) derivatives reported here include unambiguous examples of trivalent An-ligand multiple bonds, and their synthesis was facilitated by adopting a scaled-down prechoreographed approach using Ce^III^ synthetic surrogates. The elucidation of periodic trends across the 5f series, and comparisons to the 4f elements, is often grounded in the ability to isolate and characterize homologous series of molecules and driven by the establishment of Th and U chemistry first and subsequently followed by transuranium congeners. Consequently, the work reported here highlights an instance where nonaqueous low-valent transuranium chemistry provides the bonding motif precedent enabling comparison of M=C bonding to Ln^III^ congeners and early An=C bonding in other An oxidation states. A preliminary assessment of reactivity has introduced multiple bond metathesis to transuranium chemistry, together with prior examples of An^IV-VI^ reactivity now extending the range of An metallo-Wittig reactions to encompass oxidation states III-VI overall and providing an An^III^ comparison to Ln^III^ congeners.

## Supporting Information Available

Experimental and computational details and X-ray crystallographic, spectroscopic, magnetic, and quantum chemical calculations (PDF)

## Accession Codes

CCDC 2125323–2125331 contain the supplementary crystallographic data for this paper. These data can be obtained free of charge via www.ccdc.cam.ac.uk/data_request/cif, or by emailing data_request@ccdc.cam.ac.uk, or by contacting The Cambridge Crystallographic Data Centre, 12 Union Road, Cambridge CB2 1EZ, UK; fax: +44 1223 336033. All other data are available from the authors on reasonable request.
