# Supplementary material for: Carbene Complexes of Neptunium
Source: J Am Chem Soc. 2022 May 24;144(22):9764–74. doi: 10.1021/jacs.2c02152 (PMC9490846; doi:10.1021/jacs.2c02152)
Supplement: Supplementary file 1 — ja2c02152_si_001.pdf [file ja2c02152_si_001.pdf]

– *Supporting Information* –

Carbene Complexes of Neptunium

Conrad A. P. Goodwin,[1,2] Ashley J. Wooles,[1] Jesse Murillo,[2] Erli Lu,[1] Josef T. Boronski,[1] Brian L. Scott,[3] Andrew J. Gaunt,[2*] and Stephen T. Liddle[1*]

[1] Department of Chemistry and Centre for Radiochemistry Research, The University of Manchester, Oxford Road, Manchester, M13 9PL, UK.

[2] Chemistry Division, Los Alamos National Laboratory, Los Alamos, New Mexico 87545, USA.

[3] Materials Physics & Applications Division, Los Alamos National Laboratory, Los Alamos, New Mexico, 87545 (USA).

*To whom correspondence should be addressed: gaunt@lanl.gov; steve.liddle@manchester.ac.uk

Table of Contents

S1. Experimental details S2

S2. Selected photographs taken during the syntheses S17

S3. Crystallography S20

S4. Molecular structures S24

S5. UV-vis-NIR spectra S33

S6. NMR spectra S36

S7. ATR-IR spectra........ S49

S8. Magnetic moments determined by NMR spectroscopy (Evans method) S50

S9. Computational studies S50

S10. References S88

S1. Experimental details

General considerations

Caution! *Compounds of ^{237}Np radionuclide decay principally through α-emission (Q_α = 4.958 MeV, $t_{1/2}$ = 2.144(7) × 10^6 years)[1] with a relatively high specific-activity (a = 26.04 MBq g^{-1}) in comparison to ^{238}U and ^{232}Th requiring analyses of hazards and implementation of additional safety controls. ^{237}Np establishes a secular equilibrium (asymptotical concentration at 34.6 ppb) with the potent β-emitter ^{233}Pa ($t_{1/2}$ = 26.975(13) days, a = 777 TBq g^{-1}) and associated = γ-ray emission (most significant γ-branching ratio for ^{233}Pa is 39% for the 312 keV line).* Hence, all studies that involved manipulation of ^{237}Np material were conducted in a specialist transuranium radiological designated area equipped with high efficiency particulate in air (HEPA) filtered hoods and in negative pressure gloveboxes. Safety controls included continuous air monitoring for airborne α-emitting particles and use of hand-held radiation monitoring equipment. Entrance to the laboratory space was controlled with a hand and foot radiation monitoring instrument and a full body personal contamination monitoring station. The handling of free-flowing solids was restricted to be within negative pressure gloveboxes equipped with HEPA filters. In addition to standard laboratory PPE, aqueous solutions were handled using multiple layers of gloves (of a material compatible with the chemicals being handled) combined with DuPont™ Tyvek® 400 sleeves to provide overlapping coverage of the arms. Due to these radiological hazards, elemental analyses were not possible.

Unless otherwise described, all syntheses and manipulations were conducted under UHP argon (AirGas) or UHP helium (AirGas) with rigorous exclusion of oxygen and water using Schlenk line and glove box techniques (employing a negative-pressure, transuranium-capable, MBraun LabMaster, helium atmosphere glovebox where required). 4 Å molecular sieves were activated by heating for 36 hrs at 200 °C, 10^{-4} mbar. Anhydrous DME (Sigma Aldrich) was transferred onto activated 4 Å molecular sieves, stored for 1 week prior to use, and degassed before use. Anhydrous Et_2O containing BHT (100 ppm, Sigma Aldrich) was degassed, distilled from Na_2Ph_2CO, stored over

activated 4 Å molecular sieves for 1 week and degassed again before use. *d*$_8$-THF, *d*$_6$-benzene, anhydrous *n*-hexane and anhydrous toluene (Sigma Aldrich) were stored over activated 4 Å molecular sieves and degassed before use. All solvents were tested with a dilute THF solution of Na_2Ph_2CO (150 mg Ph_2CO in 20 mL of THF with an excess of Na metal) such that ethereal solvents (including *d*$_8$-THF) required 1 drop / mL to retain purple coloration and hydrocarbon solvents (including *d*$_6$-benzene) required 1 drop / 2 mL.

The compounds $[Li_2\{C(PPh_2NSiMe_3)_2\}]_2$ ($[Li_2BIPM^{TMS}]_2$),[2,3] $[Rb(BIPM^{TMS}H)]$,[4] $[Np^{IV}Cl_4(DME)_2]$,[5] $[Np^{III}I_3(THF)_4]$,[6] 1,3,4,5-tetramethylimidazole-2-thione ($I^{Me4}{=}S$),[7] 1,3,4,5-tetramethylimidazol-2-ylidene (I^{Me4}),[8] benzyl potassium,[9] and $[(BIPM^{TMS}H)UI_2(THF)]$[10] were prepared as previously described. $[U^{IV}Cl_4(DME)_2]$ was prepared by dissolving UCl_4 in DME prior to use. $[CeI_3(THF)_4]$ was prepared *in situ* by stirring CeI_3 in THF prior to use.

The glovebox atmosphere was maintained with a standalone Vacuum Atmosphere Genesis™ oxygen and moisture removal system, and atmosphere suitability was verified using a dilute toluene solution of $[Ti(Cp)_2(\mu\text{-}Cl)]_2$ (200 mg of commercial $[Ti(Cp)_2(Cl)_2]$ reduced over an excess of Zn powder in 20 mL of toluene, and filtered) prior to any manipulations, such that the residue dried to a dark green color each time (a color change to yellow or orange indicates decomposition of the Ti test compound and that atmospheric O_2/H_2O levels are too high to be conducive to this chemistry, requiring removal to lower levels before performing reactions/exposing reagents to the glovebox atmosphere). All glassware, and glass-fiber filter discs, was stored in a vacuum oven (>150 °C) for 24 hrs prior to being brought into the glovebox, and FEP (fluorinated ethylene propylene) NMR tube liners were brought into the glovebox *via* overnight or multi-hr vacuum cycles in the antechamber port.

Crystals for single-crystal X-ray diffraction studies were mounted either in Fomblin oil on a micromount (**2**) or in Paratone-N or NVH oil inside 0.5 mm quartz capillaries (Charles Supper) (**1**,

3-6). The quartz capillaries were inserted through silicone stoppers and placed inside test tubes to allow handling inside the transuranium glovebox while mounting crystals without contaminating the exterior surface of the capillary. The capillaries were then cut with nail clippers to appropriate size for mounting on a goniometer. The ends of the cut capillaries were sealed with hot capillary wax before being removed from the glovebox for coating with clear nail varnish (Hard as Nails™) to provide shatter-resilience.[11] During the clipping and wax sealing steps, care must be taken to avoid the capillary touching any contaminated surfaces (this is achieved by the introduction of fresh petri dishes, forceps, clippers, and wax, as needed in conjunction with careful handling techniques to avoid contamination transfer). Following removal from the glovebox, the exterior surfaces of the capillaries were monitored with α-particle detection instruments prior to transport to the X-ray diffraction laboratory.

Solution phase electronic absorption spectra were collected at ambient temperature using a Varian Cary 6000i UV/vis/NIR spectrometer. The solution was contained in a low volume (1 mL) screw-capped quartz cuvette (1 cm path length) that was loaded in a transuranium glovebox using Parafilm™ to protect the exterior surface of the cuvette and cap from radioactive contamination (Parafilm™ is removed in a fume hood and exterior surfaces of the cuvettes were monitored with α-particle detection instruments prior to data acquisition). Data was collected from 40,000 to 6,250 cm^{-1} (250 to 1,600 nm).

For NMR spectroscopy, a solution was loaded into a fresh FEP NMR tube liner that was protected from surface contamination with Parafilm™ while inside a transuranium glovebox. The liner was sealed with two PTFE plugs, brought out of the glovebox and verified to be free of surface contamination after the Parafilm™ was removed (using α-particle detection instruments and a Ludlum 3030E instrumentto detect both α- and β-particles on smear surveys of the exterior surfaces) and the liner loaded into a J. Young tap appended 5 mm NMR tube. The headspace was then

evacuated and refilled with He to provide an inert atmosphere headspace above the sample and exterior NMR tube/cap surfaces surveyed to check for contamination before transport to the NMR laboratory. NMR data collection was performed on a 400 MHz Bruker Advance II at room temperature unless otherwise indicated. The spectra were referenced to internal solvent residuals (^{1}H and ^{13}C) or externally to 10% TMS in $CDCl_3$ (^{31}P and ^{77}Se) *via* Equation S1, which is the IUPAC recommended convention.

$$\Delta\,(Hz) = \frac{SR^{1H}}{SF^{1H}} \times SF^{NUC}$$

Equation S1. Where SR^{1H} is the spectrum reference frequency (in Hz) of a reference ^{1}H NMR spectrum collected with TMS set to 0 ppm collected under the same experimental conditions; SF^{1H} is the spectrometer frequency (in MHz) for the ^{1}H nucleus; SF^{NUC} is the spectrometer frequency (in MHz) of the nucleus in question. The answer is given in Hz.

Where ε values are reported for molecular complexes below there is a modest error due to the small quantities of weighed material, as is nearly always the case when these values are reported from synthetic chemistry (as opposed more rigorous and quality-assured analytical determination methods that are generally unfeasible to apply to non-aqueous synthetic chemistry). Nonetheless, the ε values we determine herein are still useful metrics that we determine based on weight of crystal dissolved and solvent weight, but should not be used as analytically to assay these compunds.

ATR-IR spectra of **4Ce** and **6Ce** were recorded on a Bruker Alpha spectrometer with a Platinum-ATR module in the glovebox.

CHN microanalyses on **4Ce** and **6Ce** were carried out by London Metropolitan University.

Synthesis of [(BIPM^{TMS}H)Np^{III}(Cl)(μ-Cl)$_3$Np^{III}{μ-ClLi(DME)(OEt$_2$)}(BIPM^{TMS}H)] (1)

In a 20 mL glass scintillation vial with a PTFE-coated stirrer bar, solid [$Np^{IV}Cl_4(DME)_2$] (37.0 mg, 66 μmol) was mixed with 0.5 equiv of solid [Li_2(BIPM^{TMS})]$_2$ (37.7 mg, 33 μmol) and cooled to −35 °C in the glovebox freezer. Et_2O (1.5 mL) and toluene (1.5 mL) were mixed together and also cooled to −35 °C. Immediately upon removal from the freezer, the solvent mixture was added to the solid mixture and stirred for 1 minute. No color change was apparent and the mixture was placed back in the freezer at −35 °C for 15 minutes. The yellow suspension with visible chunks of undissolved materials was removed from the freezer and stirred for 2 minutes, then placed back in the freezer at −35 °C for 15 minutes. The yellow/brown suspension was removed from the freezer and stirred for 3 minutes (still with visible undissolved white-ish solid – presumed [Li_2(BIPM^{TMS})]$_2$ or possible LiCl salt elimination). The mixture was placed back in the freezer at −35 °C for 150 minutes. The resultant yellow/orange suspension was removed from the freezer and stirred for 20 minutes resulting in an orange suspension which was then allowed to stir further at room temperature overnight. Volatiles were removed *in vacuo* from the orange solution over a solid yellow/off-white powder. The solids were extracted with toluene (3 mL) to separate from LiCl, filtered through Celite packed on top of a glass fiber filter circle in a glass pipette to afford a deep orange filtrate, that was then concentrated *in vacuo* to ~ 1 mL in volume. The solution was stored in the glovebox freezer at −35 °C overnight. A tiny wisp of powder was visible in the bottom of the vial, so the still deep orange solution was layered with Et_2O (2 mL) and stored in the glovebox freezer at −35 °C for 3 days. No further precipitate and no crystals were evident. Volatiles were removed in vacuo and the resultant solid dissolved in Et_2O (1 mL) to afford a deep orange solution. After storage in the glovebox freezer at −35 °C for 3 days, several orange block-shaped crystals had deposited, which were determined by single-crystal X-ray diffraction to be **1**.

Synthesis of [{(BIPM^{TMS})U^{IV}}$_2$(μ-Cl)$_6${Li(DME)}$_2$] (2)

In a 20 mL glass scintillation vial with a PTFE-coated stirrer bar, Et_2O (1 ml) was added to [$U^{IV}Cl_4(DME)_2$] (30 mg, 54 μmol) and stored at -35 °C for 1 hr. In a separate 20 mL glass scintillation vial with a PTFE-coated stirrer bar, toluene (1ml) was added to [$Li_2(BIPM^{TMS})]_2$ (61.1 mg, 107 μmol) and stored at -35 °C for 1 hr. The toluene solution of [$Li_2(BIPM^{TMS})]_2$ was then added to the Et_2O solution of [$U^{IV}Cl_4(DME)_2$] to afford a pale yellow suspension. This suspension was stood at room temperature without stirring for 72 hrs, after which time the brown crystals of **2** were deposited in the vial. The identity of **2** was confirmed by single-crystal X-ray diffraction studies. Yield: 22 mg, 36%. Since the sole purpose of this synthesis was to scope the analogous Np preparation no data other than the single-crystal X-ray diffraction molecular structure were collected.

Synthesis of [(BIPM^{TMS}H)Ce^{III}(I)$_2$(THF)] (3Ce)

Complex **3Ce** was synthesized as previously reported,[12] with some modifications. In a 20 mL glass scintillation vial with a PTFE-coated stirrer bar, CeI_3 (23 mg, 44 μmol) was stirred with THF (1.5 mL) at room temperature for 5 minutes which gave a turbid colorless solution with white solids. Solid [Rb(BIPM^{TMS}H)] (28.3 mg, 44 μmol, 1 equiv.) was added in a 2 portions which caused the mixture to immediately turn pale yellow with concomitant dissolution of the white solids, followed by rapid precipitation of fine white solids (presumably RbI). The mixture was stirred for 10 minutes and then reduced to dryness *in vacuo*. The yellow solids were suspended in toluene (1 mL) and warmed gently (45 °C on a hot plate). Once cooled to room temperature, the mixture was centrifuged (5 minutes, 5,000 rpm) and filtered into a 4 mL glass vial through two glass microfiber filter discs packed in a glass pipette. The yellow solution was concentrated to the point of incipient crystallization, warmed gently (45 °C on a hot plate) and then stored at room temperature overnight (16 hrs). Several flaky colorless crystals grew and these were inspected by single-crystal X-ray diffraction and found to be **3Ce**. Characterization data on this material matched the previously reported data.[12] The poor quality of the crystals meant that this motif was not extended to studies with Np.

Synthesis of [(BIPM^{TMS}H)Ce^{III}(I)$_2$(I^{Me4})] (4Ce)

In a 20 mL glass scintillation vial with a PTFE-coated stirrer bar, CeI_3 (23 mg, 44 µmol) was stirred with THF (1.5 mL) at room temperature for 5 minutes which gave a turbid colorless solution with white solids. Solid [Rb(BIPM^{TMS}H)] (28.3 mg, 44 µmol, 1 equiv.) was added in a 2 portions which caused the mixture to immediately turn pale yellow with concomitantdissolution of the white solids, followed by rapid precipitation of fine white solids (presumably RbI). The mixture was stirred for 10 minutes and then reduced to dryness *in vacuo*. The yellow solids were suspended in toluene (1.5 mL) and warmed gently (45 °C on a hot plate). Once cooled to room temperature, the mixture was centrifuged (5 minutes, 5,000 rpm) and filtered into a 4 mL glass vial through two glass microfiber filter discs packed in a glass pipette. Solid I^{Me4} (5.2 mg, 42 µmol, 0.95 equiv.) was added to the yellow solution, which upon gentle agitation became more intensely yellow as the I^{Me4} dissolved. Manual agitation for 2 minutes resulted in the precipitation of a substantial quantity of pale yellow solids. The mixture was heated strongly (130 °C on a hot plate) and gently refluxed inside the vial for ~2 minutes which resulted in most of the solids redissolving. The yellow solution was then filtered into a fresh 4 mL glass vial through two glass microfiber filter discs packed in a glass pipette while still warm and then stored at room temperature overnight (16 hrs). Large yellow blocks formed and these were inspected by single-crystal X-ray diffraction and found to be **4Ce**. Anal. Calcd for $C_{38}H_{51}CeI_2N_4P_2Si_2$: C, 42.42; H,4.78 ; N, 5.21 %. Found: C, 42.79; H, 4.94; N, 5.08%. ^{1}H NMR (d_8-THF, 400.13 MHz, 298 K): δ 1.21 (br s, $\nu_{½}$ = 370 Hz), 3.23 (br s, $\nu_{½}$ = 47 Hz), 5.58 (br s, $\nu_{½}$ = 180 Hz), 6.69 (br s, $\nu_{½}$ = 29 Hz), 6.99 (br s, $\nu_{½}$ = 40 Hz). These resonances could not be assigned due to their broad natures and the presence of contaminant BIPM^{TMS}H$_2$ (at –0.15 ppm) and toluene giving overlapping peaks. The low solubility of **4Ce** precluded the measurement of its magnetic moment by the Evans' method and ^{13}C and ^{29}Si NMR measurements. ^{31}P{^{1}H} NMR (d_8-THF, 161.94 MHz, 298 K): δ −17.94 (br) ppm. ATR-IR ν/cm^{-1}: 3057 (w), 2944 (2), 14.36 (s), 1372 (m), 1261 (m), 1143 (s), 1100(m), 1066 (s), 999 (w), 936 (m), 833 (s), 782 (w), 762 (m), 742 (m), 723 (m), 695 (m), 659 (m), 612 (m), 591 (m), 549 (m), 509 (w), 466 (w), 440 (w).

Synthesis of [(BIPM^{TMS})Ce^{III}(I)(DME)] (5Ce)

In a modification of the previously reported procedure,[12] a 20 mL glass scintillation vial with a PTFE-coated stirrer bar and CeI_3 (23 mg, 44 µmol) was stirred with THF (1.5 mL) at room temperature for 5 minutes which gave a turbid colorless solution with white solids. Solid [Rb(BIPM^{TMS}H)] (28.3 mg, 44 µmol, 1 equiv.) was added in a 2 portions which caused the mixture to immediately turn pale yellow with concomittent dissolution of the white solids, followed by rapid precipitation of fine white solids (presumably RbI). The mixture was stirred for 5 minutes and then dried *in vacuo* to a pale yellow powder. DME (1.5 mL) was added, and then solid KBn (5.7 mg, 44 µmol, 1 equiv.) was added to the pale yellow suspension in several portions which caused the mixture to immediately turn from pale yellow to a slightly more intense yellow – the vivid orange color of the KBn discharged rapidly as each portion dissolved. The cloudy mixture was stirred for a further 5 minutes and then reduced to a yellow powder *in vacuo*. Toluene (1.5 mL) and DME (3 drops) were added to the yellow solids, which was then warmed gently (45 °C on a hot plate). Once cooled to room temperature, the mixture was centrifuged (5 minutes, 5,000 rpm) and filtered into a 4 mL glass vial through two glass microfiber filter discs packed in a glass pipette. The yellow solution was concentrated to ~0.5 mL which caused a large quantity of pale yellow solids to form on the vial walls. The mixture was heated strongly (130 °C on a hot plate) and gently refluxed inside the vial for ~2 minutes which resulted in all of the solids redissolving. The yellow solution was stored at room temperature overnight (16 hrs). Large yellow blocks formed and these were inspected by single-crystal X-ray diffraction and found to be **5Ce**. Characterization data on this material matched the previously reported data.[12]

Synthesis of [(BIPM^{TMS})Ce^{III}(I)(I^{Me4})$_2$] (6Ce)

In a 20 mL glass scintillation vial with a PTFE-coated stirrer bar, CeI_3 (23 mg, 44 µmol) was stirred with THF (1.5 mL) at room temperature for 5 minutes which gave a turbid colorless solution with white solids. Solid [Rb(BIPM^{TMS}H)] (28.3 mg, 44 µmol, 1 equiv.) was added in a 2 portions which caused the mixture to immediately turn pale yellow with concomitant dissolution of the white solids,

followed by rapid precipitation of fine white solids (presumably RbI). The mixture was stirred for 10 minutes and then solid KBn (5.7 mg, 44 μmol, 1 equiv.) was added in several portions which caused the mixture to immediately turn from pale yellow to a slightly more intense yellow – the vivid orange color of the KBn discharged instantaneously as each portion dissolved. The cloudy mixture was stirred for a further 15 minutes and then reduced to a yellow powder *in vacuo*. The yellow solids were suspended in toluene (1.5 mL) and warmed gently (45 °C on a hot plate). Once cooled to room temperature, the mixture was centrifuged (5 minutes, 5,000 rpm) and filtered into a 4 mL glass vial through two glass microfiber filter discs packed in a glass pipette. Solid I^{Me4} (10 mg, 78 μmol, 1.78 equiv.) was added to the yellow solution, which upon gentle agitation became more intensely yellow as the I^{Me4} dissolved. Manual agitation for 2 minutes resulted in the precipitation of a substantial quantity of yellow solids. The mixture was heated strongly (130 °C on a hot plate) and gently refluxed inside the vial for ~2 minutes which resulted in all of the solids redissolving. The yellow solution was stored at room temperature overnight (16 hrs). Large yellow blocks formed and these were inspected by single-crystal X-ray diffraction and found to be **6Ce**. Despite multiple attempts, satisfactory elemental analyses data could not be obtained, attributed to incomplete combustion due to the instrument temperature limit. ^{1}H NMR (d_8-THF, 400.13 MHz, 298 K): δ –3.78 (br s, $\nu_{½}$ = 310 Hz, 4 H, BIPM^{TMS} Ar *p*-C<u>*H*</u> × 4), 1.86 (br s, $\nu_{½}$ = 19 Hz, 18 H, BIPM^{TMS} Si(C<u>*H*</u>$_3$)$_3$ × 2), 6.11 (br s, $\nu_{½}$ = 71 Hz, 8 H, BIPM^{TMS} Ar *o/m*-C<u>*H*</u> × 8), 6.66 (br s, $\nu_{½}$ = 26 Hz, 8 H, BIPM^{TMS} Ar *o/m*-C<u>*H*</u> × 4). The low solubility of **6Ce** precluded the measurement of its magnetic moment by the Evans' method and ^{13}C and ^{29}Si NMR measurements. $^{31}P\{^{1}H\}$ NMR (d_8-THF, 161.94 MHz, 298 K): δ −37.04 (br) ppm. ATR-IR ν/cm^{-1}: 3049 (w), 2947 (w), 1434 (m) , 1368 (m), 1236 (s), 1102 (m), 1090 (m), 1062 (s), 826 (s), 763 (m), 750 (m),731 (m),698 (m), 649 (m), 601 (m), 548 (m), 509 (m),467 (m), 435 (m).

Synthesis of [(BIPM^{TMS}H)Np^{III}(I)$_2$(I^{Me4})] (4Np)

In a 20 mL glass scintillation vial with a PTFE-coated stirrer bar, solid [Rb(BIPM^{TMS}H)] (21 mg, 33 μmol, 1 equiv.) was added in 2 portions to a turbid orange suspension of [Np^{III}I$_3$(THF)$_4$] (30 mg, 33

μmol) in THF (1.5 mL) at room temperature, which caused the mixture to immediately turn cloudy yellow with concomitant precipitation of fine white solids (presumably RbI). The mixture was stirred for 30 minutes and then reduced to dryness *in vacuo*. The orange solids were washed with hexane (1 × 4 mL) and dried again to an orange powder. Toluene (3 mL) was added and the mixture was stirred for several minutes then filtered into a 4 mL glass vial through two glass microfiber filter discs packed in a glass pipette to give an orange solution. Solid I^{Me4} (4.1 mg, 33 μmol, 1 equiv.) was added which caused the orange solution to rapidly turn red and somewhat cloudy as the solid I^{Me4} dissolved with manual agitation. The mixture was concentrated to 2 mL which caused some colorless solids to precipitate, and the solution was filtered into a 4 mL glass vial through two glass microfiber filter discs packed in a glass pipette. Storage of this red solution at –35 °C for 48 hrs caused more colorless solids to form, and the solution was filtered again as above into a 4 mL glass vial. The solution was then concentrated to 0.5 mL and layered with hexane (0.5 mL). Storage of the layered solution for 15 minutes at room temperature resulted in large ruby-red planks of **4Np** to form. The crystals were washed with hexane at room temperature (2 × 2 mL) to give **4Np** as flowing red planks. Yield: 6.2 mg, 16%. ^{1}H NMR (d_6-benzene, 400.13 MHz, 298 K): δ −54.61 (br s, $\nu_{½}$ = 17 Hz, 1 H, $BIPM^{TMS}H$ *ipso*-C<u>*H*</u>), −4.79 (s, 18 H, $BIPM^{TMS}H$ Si(C<u>*H*</u>$_3$)$_3$ × 2), 1.82 (s, 6 H, I^{Me4} C(C<u>*H*</u>$_3$) × 2), 10.61 (s, 6 H, I^{Me4} N(C<u>*H*</u>$_3$) × 2). $^{13}C\{^{1}H\}$ NMR (d_6-benzene, 100.62 MHz, 298 K): δ −27.69 (s, $BIPM^{TMS}H$ Si(<u>*C*</u>H$_3$)$_3$ × 2), 4.35 (t, *J* = 1.7 Hz, $BIPM^{TMS}H$ *ipso*-<u>*C*</u>H), 11.80 (s, I^{Me4} N(<u>*C*</u>H$_3$) × 2) ppm. The triplet at 4.35 ppm is only tentatively assigned due to the unusual chemical shift. Peaks attributable to the I^{Me4} C(<u>*C*</u>H$_3$) and <u>*C*</u>(CH$_3$) resonances could not be located, nor could peaks be assigned that were definitively due to complexed $BIPM^{TMS}H$, rather than trace H_2BIPM^{TMS}. $^{31}P\{^{1}H\}$ NMR (d_6-benzene, 161.94 MHz, 298 K): δ −488.05 ppm. UV-vis-NIR (toluene): λ_{max} (cm^{-1}; *ε*) 363 (27,533, *1,490*), 429 (23,310, *1,270*), 568 (17,618, *210*), 619 (16,150, *70*), 674 (14,846, *40*), 802 (12,466, *50*), 868 (11,526, *50*), 922 (10,844, *20*), 946 (10,571, *20*), 965 (10,361, *20*), 1,001 (9,986, 30*),* 1,008 (9,921, *30*), 1,031 (9,699, *20*), 1,046 (9,564, *20*), 1,144 (8,740, *10*), 1,152 (8,678, *10*), 1,210 (8,262, *10*), 1,335 (7,490, *20*), 1,352 (7,398, *30*), 1,412 (7,084, *20*) nm.

Synthesis of [(BIPM^{TMS})Np^{III}(I)(DME)] (5Np)

In a 20 mL glass scintillation vial with a PTFE-coated stirrer bar, solid [Rb(BIPM^{TMS}H)] (28.3 mg, 44 μmol, 1 equiv.) was added in 2 portions to a turbid orange suspension of [Np^{III}I$_3$(THF)$_4$] (40 mg, 44 μmol) in THF (1.5 mL) at room temperature, which caused the mixture to immediately turn orange, and then cloudy and yellow with concomitant precipitation of fine white solids (presumably RbI). The mixture was stirred for 5 minutes and then dried *in vacuo* to an orange powder. DME (1.5 mL) was added, and then solid KBn (5.7 mg, 44 μmol, 1 equiv.) was added to the orange suspension in several portions which caused the mixture to immediately turn from orange to red/purple – the vivid orange color of the KBn discharged instantaneously as each portion dissolved. The cloudy mixture was stirred for a further 5 minutes and then reduced to a red powder *in vacuo*. Toluene (1.5 mL) and DME (3 drops) were added to the orange solids and the mixture was stirred for 1 minute at room temperature. The mixture was filtered into a 4 mL glass vial through two glass microfiber filter discs packed in a glass pipette, then concentrated to ~0.3 mL *in vacuo* without agitation which caused a red/orange seed crystal to form at the solution surface. The solution was then left at room temperature for 20 minutes which caused a single large crop of orange parallelepiped-shaped plates of **5Np** to grow. Yield: 17.4 mg, 37%. ^{1}H NMR (d_6-benzene, 400.13 MHz, 298 K): δ −6.56 (br s, $\nu_{½}$ = 34 Hz, 18 H, BIPM^{TMS} Si(C$\underline{H}_3$)$_3$ × 2), 1.74 (br s, $\nu_{½}$ = 98 Hz, 6 H, DME O(C$\underline{H}_3$) × 2) ppm. Broad features around 6.61 ($\nu_{½}$ = 311 Hz), 9.43 ($\nu_{½}$ = 488 Hz), and 14.56 ($\nu_{½}$ = 575 Hz) ppm could be BIPM^{TMS} Ph peaks or the DME C$_2\underline{H}_4$ peak, but we could not definitively assign these given the significant broadening which affects the reliability of integration. ^{13}C{^{1}H} NMR (d_6-benzene, 100.62 MHz, 298 K): δ −26.59 (s, BIPM^{TMS} Si($\underline{C}$H$_3$)$_3$ × 2) ppm. No other peaks unattributed to BIPM^{TMS}H$_2$ or a BIPM^{TMS}H species could be definitively assigned. ^{31}P{^{1}H} NMR (d_6-benzene, 161.94 MHz, 298 K): δ −789.02 ppm. UV-vis-NIR (toluene): λ_{max} (cm^{-1}; *ε*) 295 (33,921, *5,220*), 409 (24,450, *1,570*), 567 (17,649, *260*), 609 (16,426, *180*), 757 (13,210, *50*), 798 (12,528, *70*), 819 (12,216,70*9*), 834 (11,990, *50*), 852 (11,740, *60*), 865 (11,563, *70*), 893 (11,193, *60*), 982 (10,181, *50*), 1,001 (9,990, *50*), 1,332 (7,506, *30*) nm.

Synthesis of $[(BIPM^{TMS})Np^{III}(I)(I^{Me4})_2]$ (6Np)

In a 20 mL glass scintillation vial with a PTFE-coated stirrer bar, THF (1.5 mL) was added to solid $[Rb(BIPM^{TMS}H)]$ (28.3 mg, 44 µmol, 1 equiv.) and $[Np^{III}I_3(THF)_4]$ (40 mg, 44 µmol), which caused the mixture to immediately turn cloudy cloudy orange/yellow with precipitation of fine white solids (presumably RbI). The mixture was stirred for 15 minutes and then solid KBn (5.7 mg, 44 µmol, 1 equiv.) was added in several portions which caused the mixture to immediately turn from orange to red and then purple/brown. The cloudy mixture was stirred for a further 15 minutes and then reduced to an oily solid *in vacuo*. Toluene (1 mL) was added and the mixture was stirred for 5 minutes then filtered into a 20 mL glass scintillation vial through two glass microfiber filter discs packed in a glass pipette, to give a clear red/purple solution. A PTFE-coated stirrer bar was added, and then solid I^{Me4} (10 mg, 78 µmol, 1.78 equiv.) was added in several portions to the stirred solution which caused a color change from red to purple as the I^{Me4} dissolved. *Note: attempts to isolate* ***6Np*** *have been unsuccessful when 2 equiv. of I^{Me4} have been used. Similar reactions have resulted in the isolation of imidazolinium iodide salts in our hands.* The solution became slightly cloudy, but rapid stirring did not result in further precipitation unlike the synthesis of **6Ce**. The mixture was heated to a gentle reflux (130 °C on a hot plate) for 1 minute and then filtered into a 4 mL glass vial through two glass microfiber filter discs packed in a glass pipette and stored overnight (16 hrs) at room temperature which caused a small crop of red/black blocks to form (see below for yields). The small quantity of red/purple solids that remained on the filter discs was extracted with THF (1 mL) into a fresh 4 mL glass vial and dried *in vacuo* to a red powder. This was then dissolved in toluene (1 mL) after 2 minutes of gentle refluxing (130 °C hot plate) and the hot solution was stored at room temperature overnight (16 hrs) – crystals of **6Np** then grew from this aliquot once it was cooled (–35 °C) in the glovebox freezer and then again when stored again at room temperature for 20 minutes. The supernatants from both of these crops of crystals was decanted and layered with hexane (1 mL) and stored overnight (16 hrs) which caused a third crop of crystals of **6Np** to form. All three crops were washed with hexane (2 × 1 mL) separately and dried *in vacuo* to a red/purple powder. Combined

yield: combined 15.7 mg, 32%. ^{1}H NMR (d_6-benzene, 400.13 MHz, 298 K): δ −9.17 (br s, $\nu_{½}$ = 50 Hz, 18 H, BIPM^{TMS} Si(C$\underline{H}_3$)$_3$ × 2) ppm. We tentatively assign this peak due to its intensity and position which is similar to that of **5Np**. No other peak that could be definitively assigned to this complex rather than BIPM^{TMS}H$_2$, or a BIPM^{TMS}H complex (like **4Np**), could be identified. ^{13}C{^{1}H} NMR (d_6-benzene, 100.62 MHz, 298 K): Only peaks assignable to a BIPM^{TMS}H complex (like **4Np**) could be identified, presumably due to the very large number of scans needed, combined with the limitations of our sample sealing procedure. ^{31}P{^{1}H} NMR (d_6-benzene, 161.94 MHz, 298 K): δ −739.51 ppm. UV-vis-NIR (toluene): λ_{max} (cm^{-1}; ε) 306 (32,680, *4,530*), 429 (23,310, *1,520*), 496 (20,153, *1,290*), 550 (18,188, *890*), 584 (17,123, *610*), 616 (16,234, *350*), 644 (15,538, *210*), 677 (14,775, *110*), 777 (12,877, *40*), 815 (12,273, *80*), 833 (12,008, *70*), 871 (11,478, *90*), 889 (11,244, *90*), 941 (10,632, *50*), 991 (10,089, *50*), 1,006 (9,936, *80*), 1,013 (9,874, *80*), 1,069 (9,353, *20*), 1,152 (8,679, *10*), 1,196 (8,361, *10*), 1,251 (7,994, *10*) nm.

Attempted Synthesis of [(BIPM^{TMS})U^{III}(I)(DME)] (5U)

[(BIPM^{TMS}H)UI$_2$(THF)] was dissolved in DME (5 mL), stirred for several hrs, then all volatiles are removed *in vacuo* to give [(BIPM^{TMS}H)UI$_2$(DME)]. In a 20 mL glass scintillation vial with a PTFE-coated stirrer bar, [(BIPM^{TMS}H)UI$_2$(DME)] (55 mg, 49 μmol) was stirred in DME (5 mL) at room temperature for 5 minutes to give a dark blue solution. KBn (6.5 mg, 50 μmol, 1 equiv.) was added portionwise upon which a color change to brown was observed. The resulting mixture was stirred for 15 minutes after which time all the volatiles were removed *in vacuo* to afford a brown solid, which was extracted into toluene and filtered through a syringe filter to afford a pale brown solution. Attempts to prepare crystalline material was unsuccessful despite several attempts using different solvents and solvent ratios (Toluene, DME).

Attempted Synthesis of [(BIPM^{TMS})U^{III}(I)(I^{Me4})$_2$] (6U)

Attempt 1. In a 20 mL glass scintillation vial with a PTFE-coated stirrer bar, [(BIPM^{TMS}H)UI$_2$(THF)] (36 mg, 33 μmol) was stirred with toluene (3 mL) at room temperature for 5 minutes to give a dark blue solution. Solid I^{Me4} (4.2 mg, 33 μmol, 1 equiv.) was added and the resulting mixture stirred for 5 minutes to afford a dark blue/purple solution. KBn (4.3 mg, 33 μmol, 1 equiv.) was added and the resulting mixture stirred for 5 minutes after which all the KBn appeared to be consumed and the solution changed to a dark brown color with a colorless precipitate. The mixture was filtered through a syringe filter to afford a pale brown solution. Attempts to prepare crystalline material was unsuccessful despite several attempts using different solvents and solvent ratios (Toluene, Et$_2$O, THF).

Attempt 2. In a 20 mL glass scintillation vial with a PTFE-coated stirrer bar, [(BIPM^{TMS}H)UI$_2$(THF)] (36 mg, 33 μmol) was stirred with toluene (5 mL) at room temperature for 5 minutes to give a dark blue solution. Solid I^{Me4} (8.4 mg, 66 μmol, 2 equiv.) was added and the resulting mixture stirred for 30 minutes to afford a dark blue/purple solution. The solution was then stored at −30 °C for 30 minutes. KBn (4.3 mg, 33 μmol, 1 equiv.) was added and the resulting mixture stirred for 20 minutes after which time all the KBn appeared to be consumed, affording a dark blue/ brown solution with precipitation of a pale solid. Volatiles were removed from the solution to afford a sticky brown solid, which was extracted into toluene and filtered through a syringe filter to afford a pale brown solution. Attempts to prepare crystalline material was unsuccessful despite several attempts using different solvents and solvent ratios (Toluene, Et$_2$O, THF).

Reaction of 5Np with PhCHO to give PhC(H)=C(PPh$_2$NSiMe$_3$)$_2$ (7)

In a 5 mL glass scintillation vial with a glass stirrer bar, **5Np** (10.0 mg, 9.36 μmol) was dissolved 1.0 mL of d_6-benzene, forming a red solution. To this, a solution of PhCHO (1.0 mg (9.32 μmol) in 1.0 mL of d_6-benzene) was added dropwise while stirring at room temperature. The reaction was left to stir for 72 hrs. The solution was then filtered through a glass microfiber filter disk packed in a glass pipette to give a clear brown/red filtrate. The filtrate was concentrated to approximately 0.5 mL *in vacuo* and used as-eluted for NMR experiments. ^{1}H NMR (d_6-benzene, 400.13 MHz, 298 K): δ 0.37 (s, 9 H, NSi(C$\underline{H}_3$)$_3$), 0.39 (s, 9 H, NSi(C$\underline{H}_3$)$_3$), 6.78 (br, 2 H, m-Ph-C$\underline{H}$), 6.89 (br, 6 H, p-Ph-C$\underline{H}$ P and o-Ph-C$\underline{H}$), 7.02 (br, 8 H, m-Ph-C$\underline{H}$ P), 7.39 (br, 1 H, p-Ph-C$\underline{H}$), 7.78 (m, 8 H, o-Ph-C$\underline{H}$), 8.04 (dd, $^3J_{PH}$ = 28.4 and 28.3 Hz, 1 H, Ph$\underline{H}$C=CP$_2$). ^{31}P NMR (d_6-benzene, 161.94 MHz, 298 K): δ −6.75 (d, $^2J_{pp}$ = 35.2 Hz), -7.79 (d, $^2J_{pp}$ = 35.3 Hz). Analysis of the unpurified reaction mixture by NMR (^{1}H and ^{31}P) matches that of the previously reported alkene,[13] revealing the formation of PhC(H)=C(PPh$_2$NSiMe$_3$)$_2$ (7), along with minor paramagnetic species that are presumed to be Np byproducts. Further characterization of the reaction products was impractical given the scarce nature of neptunium precursors.

S2. Selected photographs taken during the syntheses

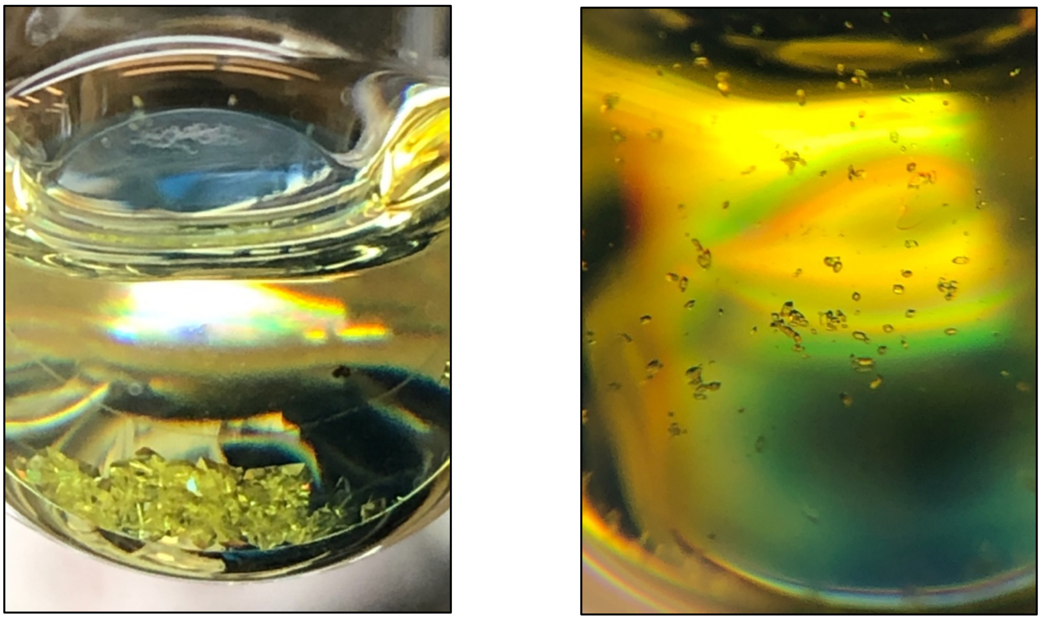

Figure S1. From left to right: 1) Crystals of **5Ce** under toluene; 2) Crystals of **6Ce** under toluene.

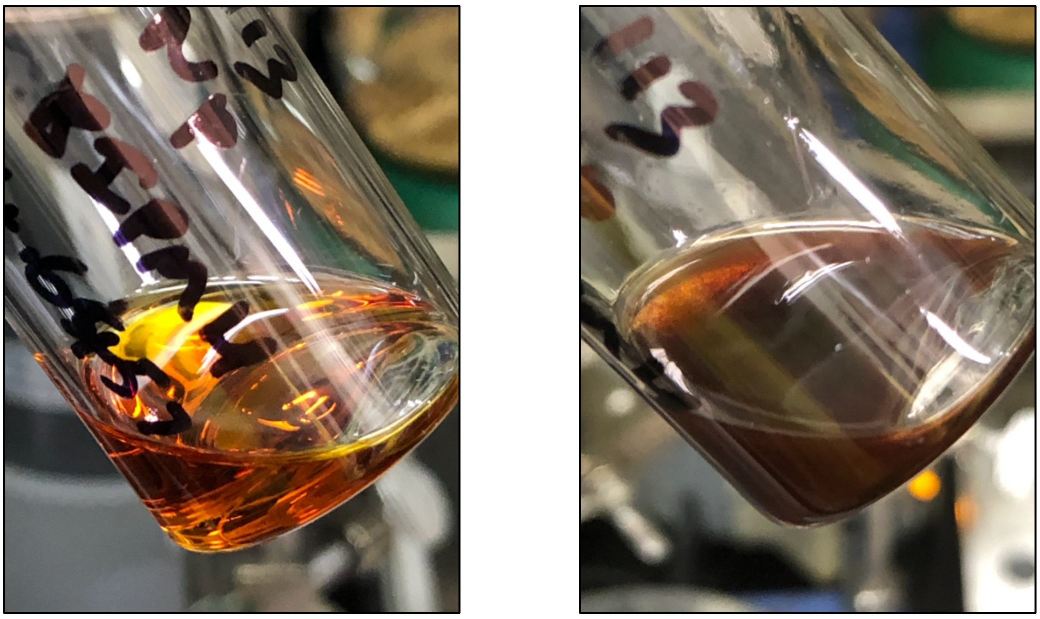

Figure S2. From left to right: 1) Solution of putative **3Np** in toluene, prior to addition of solid I^{Me4}; 2) The same material as on the left, but after addition of I^{Me4}. The pale precipitate can be clearly seen. More of this same material formed after subsequent concentration and storage at –35 °C. Only once all of this material was removed could crystals of **4Np** be isolated.

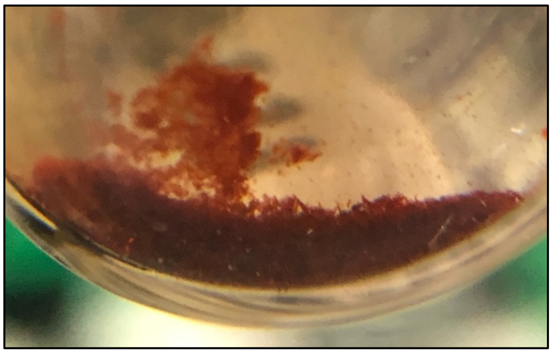

Figure S3. Crystals of **4Np** under hexane during the washing procedure.

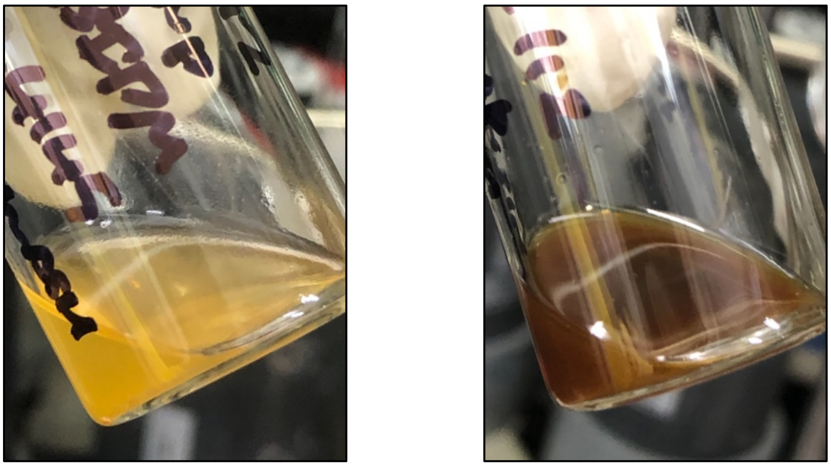

Figure S4. From left to right: 1) Suspension of putative **3Np** in THF, prior to addition of KBn; 2) The same material as on the left, but after addition of KBn.

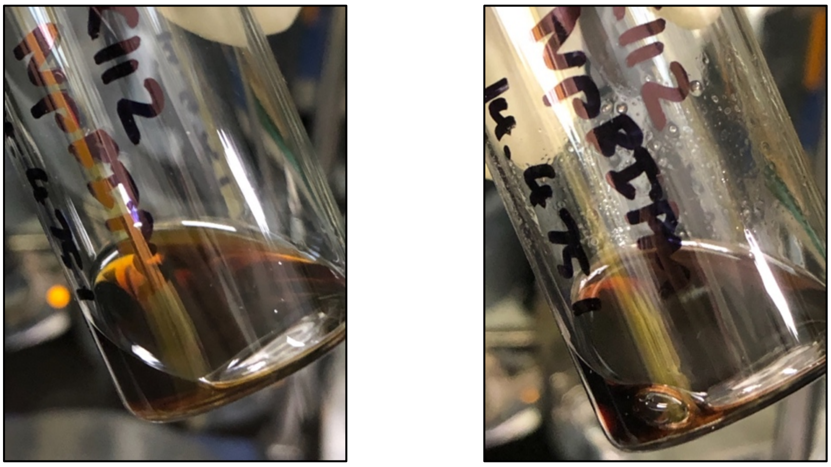

Figure S5. From left to right: 1) Suspension of putative **3Np** (from Figure S4) in toluene, prior to addition of solid I^{Me4} to form **6Np**; 2) The same material as on the left, but after addition of I^{Me4} to form **6Np**. The small quantity of precipitated material can be seen on the vial walls.

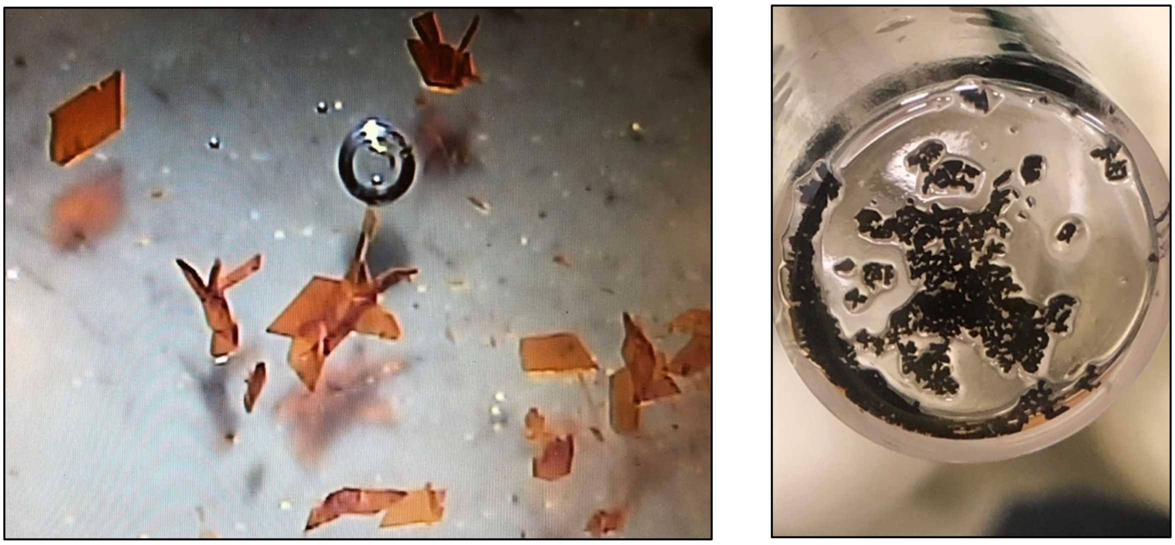

Figure S6. From left to right: 1) Crystals of **5Np** under NVH oil, viewed through a microscope; 2) Crystals of **6Np** prior to washing and drying.

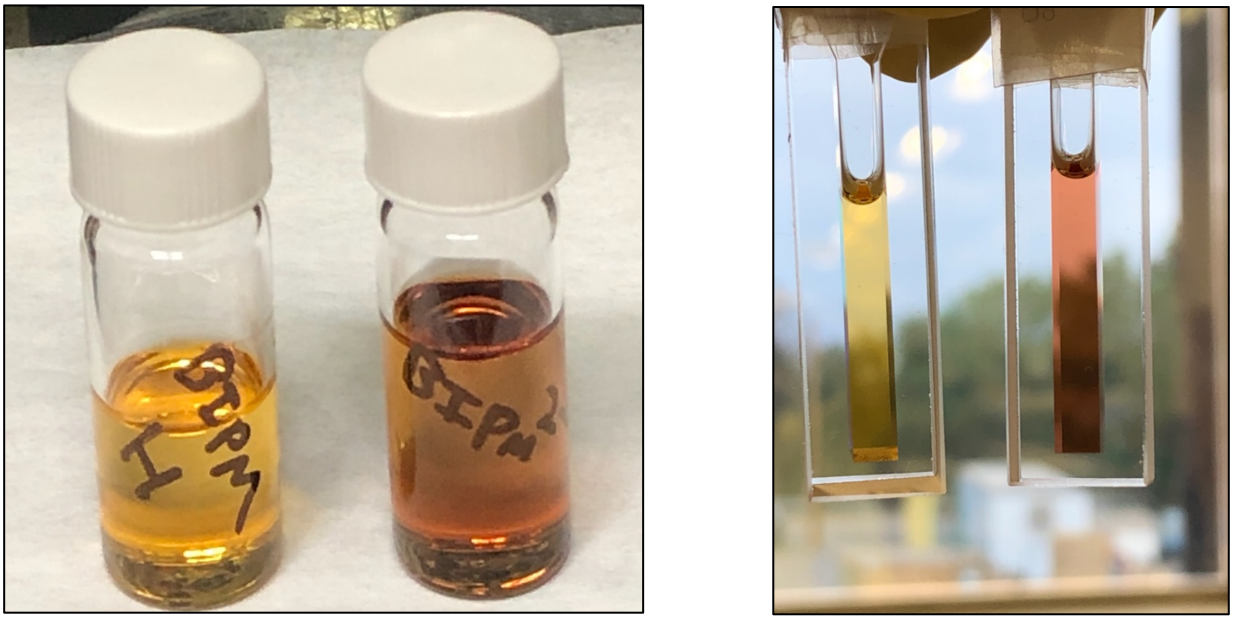

Figure S7. Solutions of **4Np** (orange, left in both images) and **6Np** (orange/red, right in both images) in toluene for UV-vis-NIR spectroscopy.

S3. Crystallography

General considerations

The crystal data for all complexes are compiled in Table S1 to Table S3. All crystals were examined with either a Bruker Apex II diffractometer equipped with an Apex II CCD detector and using mirror-monochromated Mo Kα radiation (λ = 0.71073 Å); a Bruker D8 Quest diffractometer equipped with a Photon II CPAD detector and using mirror-monochromated Mo Kα radiation (λ = 0.71073 Å) operating in shutterless mode; a Bruker microsource diffractometer equipped with a Photon III CPAD detector and using mirror-monochromated Mo Kα radiation (λ = 0.71073 Å); or a Rigaku FR-X diffractometer equipped with a HyPix 6000HE photon counting pixel array detector with mirror-monochromated Mo Kα radiation (λ = 0.71073 Å). APEX II, APEX III or CryAlisPro software were used for control and solving the unit cells prior to data collection.[14,15] Intensities were integrated from data recorded on 0.5° frames by ω rotation with 0.5s (**2**), 4s (**5Ce**), 10s (**4Ce**, **4Np**, **5Np**), 20s (**1**, **6Ce**) or 30s (**3Ce**) exposures; or by both ω and φ rotation (0.5°) with 10s (**6Np**) frame exposures. CrysAlisPro[14] was used for final unit cell determination and parameters were refined from the observed positions of all strong reflections in each data set. Analytical absorption corrections were applied to **1** and **3-6**, and face indexed absorption corrections were applied to **2**.[15] The Olex2[16] GUI was used for structure solution and refinement utilizing the ShelX software packages.[17,18] The structures were solved using ShelXT[17]; the datasets were refined by ShelXL[18] using full-matrix least-squares on all unique F^2 values, with anisotropic displacement parameters for all non-hydrogen atoms, and with constrained riding hydrogen geometries; U_{iso}(H) was set at 1.2 (1.5 for methyl groups if applicable) times Ueq of the parent atom. The largest features in final difference syntheses were close to heavy atoms and were of no chemical significance. Ortep and PovRay were employed for molecular graphics.[19,20] The CCDC deposits contain the supplementary crystal data for this article. 2125323 (**1**), 2125324 (**2**), 2125325 (**3Ce**), 2125326 (**4Ce**), 2125327 (**4Np**), 2125328 (**5Ce**), 2125329 (**5Np**), 2125330 (**6Ce**), and 2125331 (**6Np**). These data can be obtained free of charge from the Cambridge Crystallographic Data Centre via www.ccdc.cam.ac.uk/data_request/cif.

Table S1. Crystallographic data for **1**, **2**, and **3Ce**.

	**1**	**2**	**3Ce**
CCDC ref code	2125323	2125324	2125325
Formula	$C_{78}H_{118}Cl_5LiN_4Np_2O_5P_4Si_4$	$C_{88}H_{122}Cl_6Li_2N_4O_5P_4Si_4U_2$	$C_{38.5}H_{51}CeI_2N_2OP_2Si_2$
Fw	2086.19	2254.77	1069.85
Crystal syst	Monoclinic	Triclinic	Triclinic
Space group	$P2_1/c$	P-1	P-1
a, Å	11.9971(2)	12.4707(2)	9.7913(7)
b, Å	17.7775(3)	14.1981(2)	11.9571(8)
c, Å	43.6609(7)	14.6654(2)	19.1734(12)
α, °		89.2160(10)	77.161(5)
β, °	97.229(2)	73.7920(10)	77.102(5)
γ, °		87.5900(10)	83.627(5)
V, Å^3	9237.9(3)	2491.24(6)	2128.9(3)
Z	4	1	2
ρ_{calcd}, g cm^{-3}	1.500	1.503	1.669
μ, mm^{-1}	2.550	3.568	2.682
F(000)	4176	1126	1052
Cryst size, mm	0.24 × 0.12 × 0.08	0.339 x 0.295 x 0.23	0.10 x 0.10 x 0.10
Temperature, K	100(2)	150(2)	100(2)
no. reflections (unique)	95472 (18839)	35124 (10117)	19776 (8599)
R_{int}	0.054	0.0339	0.0811
R_1(wR_2) (F^2 > 2σ(F^2))[a]	0.0361 (0.0771)	0.0242, 0.0615	0.0642, 0.1307
S[a]	1.01	1.081	1.016
min./max. diff map, Å^{-3}	–0.90, 1.23	-1.153, 1.375	-1.828, 2.219

[a] $R = \sum||F_O| - |F_C||/\sum|F_O|$; $R_W = [\sum w(F_O^2 - F_C^2)^2/\sum w(F_O^2)^2]^{0.5}$; $S = [\sum w(F_O^2 - F_C^2)^2/(\text{no. data} - \text{no. params})]^{0.5}$ for all data.

Table S2. Crystallographic data for **3Np**, **4Ce**, and **4Np**.

	**4Ce**	**4Np**	**5Ce**
CCDC ref code	2125326	2125327	2125328
Formula	$C_{38}H_{51}CeI_2N_4P_2Si_2$	$C_{38}H_{51}I_2N_4NpP_2Si_2$	$C_{38.5}H_{52}CeIN_2O_2P_2Si_2$
Fw	1075.86	1172.74	959.96
Crystal syst	Monoclinic	Monoclinic	Monoclinic
Space group	*C*2/c	*C*2/c	$P2_1$/n
a, Å	19.7305(5)	19.7438(5)	9.6163(5)
b, Å	11.8965(3)	11.8689(3)	22.1127(12)
c, Å	36.6720(9)	36.4644(8)	20.7529(11)
α, °	90	90	90
β, °	98.893(2)	98.841(2)	99.555(5)
γ, °	90	90	90
V, $Å^3$	8504.3(4)	8443.4(4)	4351.7(4)
Z	8	8	4
ρ_{calcd}, g cm^{-3}	1.681	1.845	1.465
μ, mm^{-1}	2.685	4.088	1.916
F(000)	4232	4512	1928
Cryst size, mm	0.20 x 0.05 x 0.05	0.15 x 0.10 x 0.01	0.20 x 0.20 x 0.05
Temperature, K	100(2)	100(2)	120(2)
no. reflections (unique)	38348 (10051)	35797 (8638)	86241 (13220)
R_{int}	0.1183	0.0644	0.0416
$R_1(wR_2)$ ($F^2 > 2\sigma(F^2)$)[a]	0.0636, 0.1457	0.0476, 0.1145	0.0268, 0.0623
S[a]	1.124	1.033	1.048
min./max. diff map, $Å^{-3}$	-1.603, 1.996	-2.290, 1.940	-0.964, 2.380

[a] $R = \sum||F_O| - |F_C||/\sum|F_O|$; $R_W = [\sum w(F_O^2 - F_C^2)^2/\sum w(F_O^2)^2]^{0.5}$; $S = [\sum w(F_O^2 - F_C^2)^2/(\text{no. data} - \text{no. params})]^{0.5}$ for all data.

Table S3. Crystallographic data for **5Ce**, **5Np**, and **6**.

	**5Np**	**6Ce**	**6Np**
CCDC ref code	2125329	2125330	2125331
Formula	$C_{38.5}H_{52}IN_2NpO_2P_2Si_2$	$C_{48.5}H_{66}CeIN_6P_2Si_2$	$C_{48.5}H_{66}IN_6NpP_2Si_2$
Fw	1056.84	1118.21	1215.09
Crystal syst	monoclinic	monoclinic	monoclinic
Space group	$P2_1$/n	$P2_1$/n	$P2_1$/n
a, Å	9.5530(2)	15.7692(2)	15.7683(4)
b, Å	21.8585(6)	20.5022(2)	20.4116(4)
c, Å	20.4993(5)	16.3718(2)	16.3581(4)
α, °	90	90	90
β, °	99.295(2)	100.4870(10)	100.497(2)
γ, °	90	90	90
V, Å^3	4224.34(18)	5204.64(11)	5176.8(2)
Z	4	4	4
ρ_{calcd}, g cm^{-3}	1.662	1.427	1.559
μ, mm^{-1}	3.357	1.613	2.750
F(000)	2068	2272	2412
Cryst size, mm	0.20 x 0.20 x 0.05	0.20 x 0.20 x 0.05	0.20 x 0.20 x 0.05
Temperature, K	100(2)	100(2)	120(2)
no. reflections (unique)	62482 (7686)	68943 (15874)	84005 (10520)
R_{int}	0.1790	0.0978	0.1256
R_1(wR_2) ($F^2 > 2\sigma(F^2)$)[a]	0.0643, 0.1679	0.0426, 0.1025	0.0486, 0.0971
S[a]	1.051	1.040	1.126
min./max. diff map, Å^{-3}	-4.414, 4.772	-1.808, 2.465	-1.029, 2.087

[a] $R = \sum||F_O| - |F_C||/\sum|F_O|$; $R_W = [\sum w(F_O^2 - F_C^2)^2/\sum w(F_O^2)^2]^{0.5}$; $S = [\sum w(F_O^2 - F_C^2)^2/(\text{no. data} - \text{no. params})]^{0.5}$ for all data.

$$esd3 = \sqrt{(esd1)^2 + (esd2)^2}$$

Equation S2. The combined standard uncertainty from two individual metrics that have their own associated uncertainties can be calculated as the root of the sum of the square of each error. This is not strictly appropriate for combining more than two individual errors.[21]

S4. Molecular structures

Complex 1

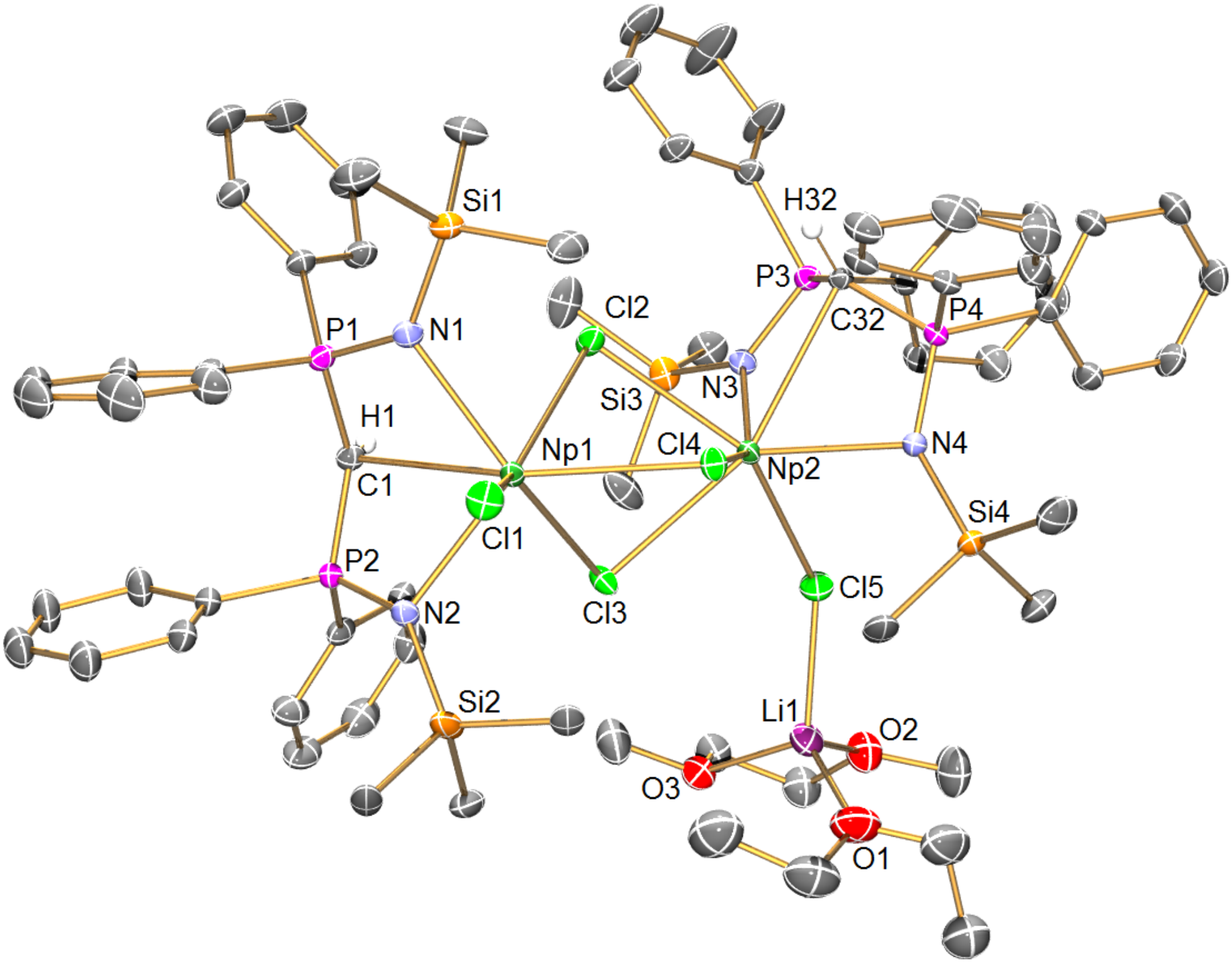

Figure S8. Solid state molecular structure of complex **1** at 100K. Displacement ellipsoids are set at 30% probability and non-methanide hydrogen atoms, disordered components and lattice solvent are omitted for clarity. Selected bond lengths [Å] and angles [°]: Np1-Cl1 2.7325(12), Np1-Cl2 2.8675(11), Np1-Cl3 2.9473(11), Np1-Cl4 2.8423(11), Np1-N1 2.451(4), Np1-N2 2.473(4), Np1-C1 2.831(4), Np2-Cl2 2.8344(12), Np2-Cl3 2.7972(11), Np2-Cl4 2.9223(11), Np2-Cl5 2.8092(13), Np2-N3 2.452(3), Np2-N4 2.467(3), Np2-C32 2.838(4), P1-C1 1.745(5), P2-C1 1.730(4), P3-C32 1.736(4), P4-C32 1.738(4). P1-C1-Np1 85.53(17), P2-C1-Np1 86.40(16), P1-C1-P2 130.2(3), P3-C32-Np2 85.35(16), P3-C32-P4 129.4(3), P4-C32-Np2 86.59(15), N1-Np1-N2 106.45(12), N3-Np2-N4 104.02(12), Np2-Cl2-Np1 92.88(3), Np2-Cl3-Np1 91.95(3), Np1-Cl4-Np2 91.56(3), Li1-Cl5-Np2 140.9(2).

Complex 2

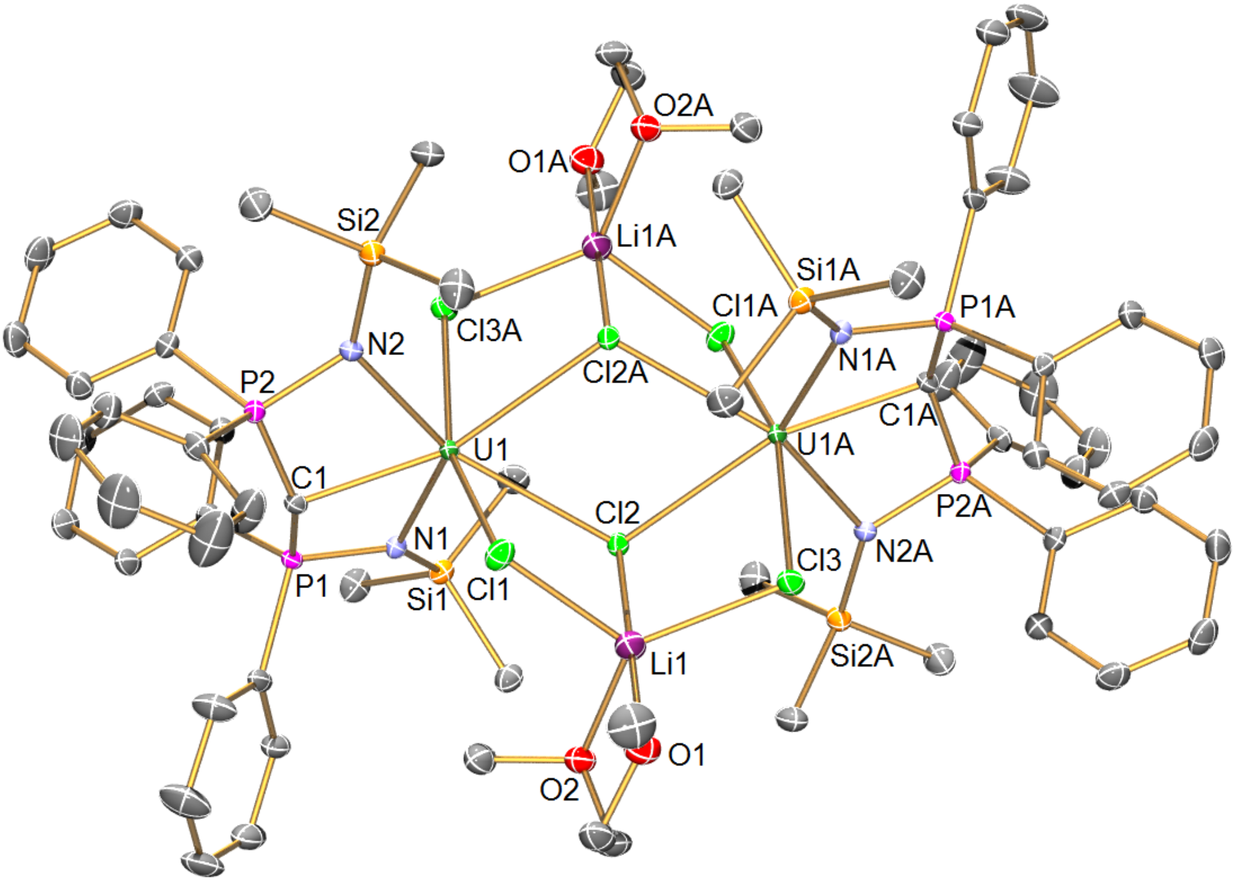

Figure S9. Solid state molecular structure of complex **2** at 150K. Displacement ellipsoids are set at 30% probability and hydrogen atoms and lattice solvent are omitted for clarity. Selected bond lengths [Å] and angles [°]: U1-Cl1 2.7309(7), U1-Cl2 2.8840(6), U1-Cl2 2.8992(6), U1-Cl3 2.7080(6), U1-N1 2.443(2), U1-N2 2.396(2), U1-C1 2.314(3), P1-N1 1.631(2), P1-C1 1.680(3), P2-N2 1.627(2), P2-C1 1.664(3), Cl1-U1-Cl2A 81.36(2), Cl1-U1-Cl2 75.933(19), Cl2-U1-Cl2A 71.85(2), Cl3A-U1-Cl1 158.79(2), Cl3A-U1-Cl2 101.99(2), Cl3A-U1-Cl2 78.015(19), N1-U1-Cl1 112.38(5), N1-U1-Cl2A 142.34(5), N1-U1-Cl2 77.74(5), N1-U1-Cl3A 87.31(5), N2-U1-Cl1 89.45(6), N2-U1-Cl2A 87.00(5), N2-U1-Cl2 155.69(6), N2-U1-Cl3A 84.60(5), N2-U1-N1 126.28(7), C1-U1-Cl1 89.15(7), C1-U1-Cl2A 151.63(7), C1-U1-Cl2 131.60(7), C1-U1-Cl3A 106.81(7), C1-U1-N1 65.88(8), C1-U1-N2 66.12(8).

Complex 3Ce

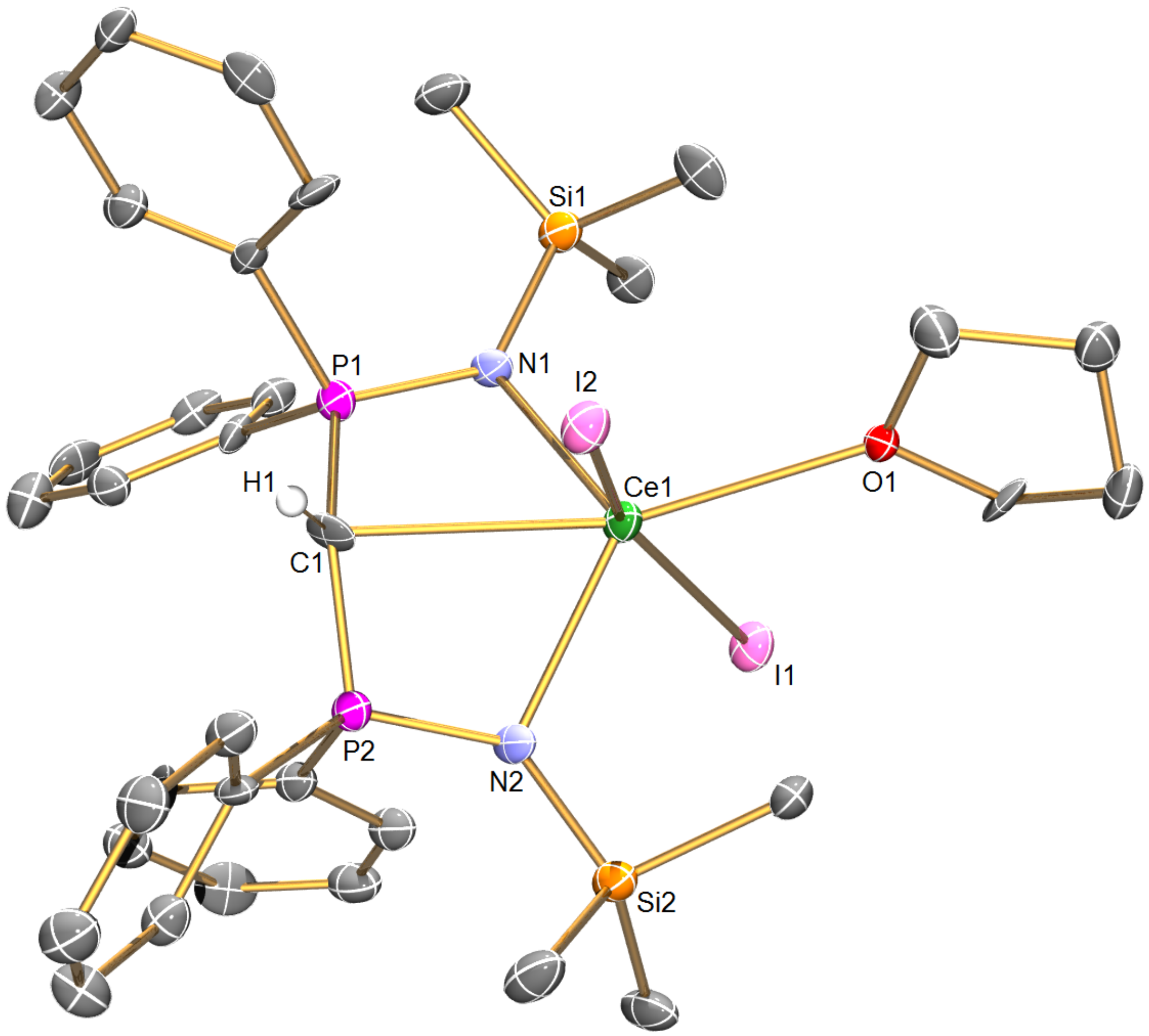

Figure S10. Solid state molecular structure of complex **3Ce** at 100K. Displacement ellipsoids are set at 50% probability and non-methanide hydrogen atoms and lattice solvent are omitted for clarity. Selected bond lengths [Å] and angles [°]: Ce1-I1 3.1504(7), Ce1-I2 3.1714(8), Ce1-O1 2.605(6), Ce1-N1 2.437(7), Ce1-N2 2.383(6), Ce1-C1 2.806(9), P1-N1 1.616(7), P1-C1 1.737(8), P2-N2 1.608(7), P2-C1 1.737(8), C1-Ce1-I1 117.48(17), C1-Ce1-I2 80.40(17), O1-Ce1-C1 159.1(2), P1-C1-P2 135.3(5).

Complex 4Ce

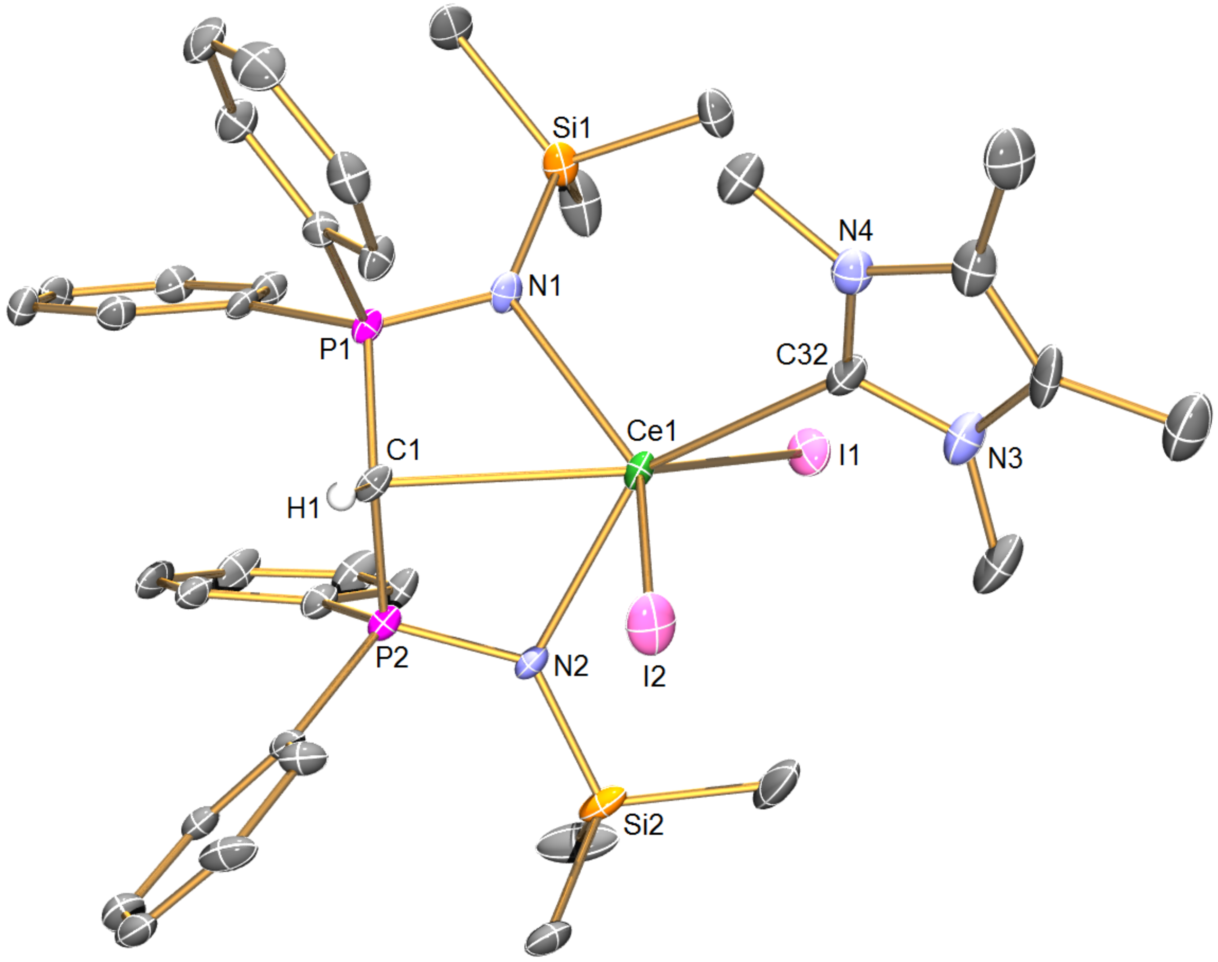

Figure S11. Solid state molecular structure of complex **4Ce** at 100K. Displacement ellipsoids are set at 50% probability and non-methanide hydrogen atoms are omitted for clarity. Selected bond lengths [Å] and angles [°]: Ce1-I1 3.1111(6), Ce1-I2 3.2092(6), Ce1-N1 2.493(6), Ce1-N2 2.448(6), Ce1-C1 2.768(6), Ce1-C32 2.731(8), P1-N1 1.608(6), P1-C1 1.731(7), P2-N2 1.612(6), P2-C1 1.761(7), I1-Ce1-I2 135.964(19), N1-Ce1-I1 91.19(13), N1-Ce1-I2 131.69(13), N1-Ce1-C1 61.26(18), N1-Ce1-C32 110.1(2), N2-Ce1-I1 94.16(13), N2-Ce1-I2 84.80(13), N2-Ce1-N1 104.94(18), N2-Ce1-C1 62.87(19), N2-Ce1-C32 144.9(2), C1-Ce1-I1 134.05(13), C1-Ce1-I2 84.01(13), C32-Ce1-I1 87.70(16), C32-Ce1-I2 70.22(16), C32-Ce1-C1 134.5(2), P1-C1-P2 128.4(4)

Complex 5Ce

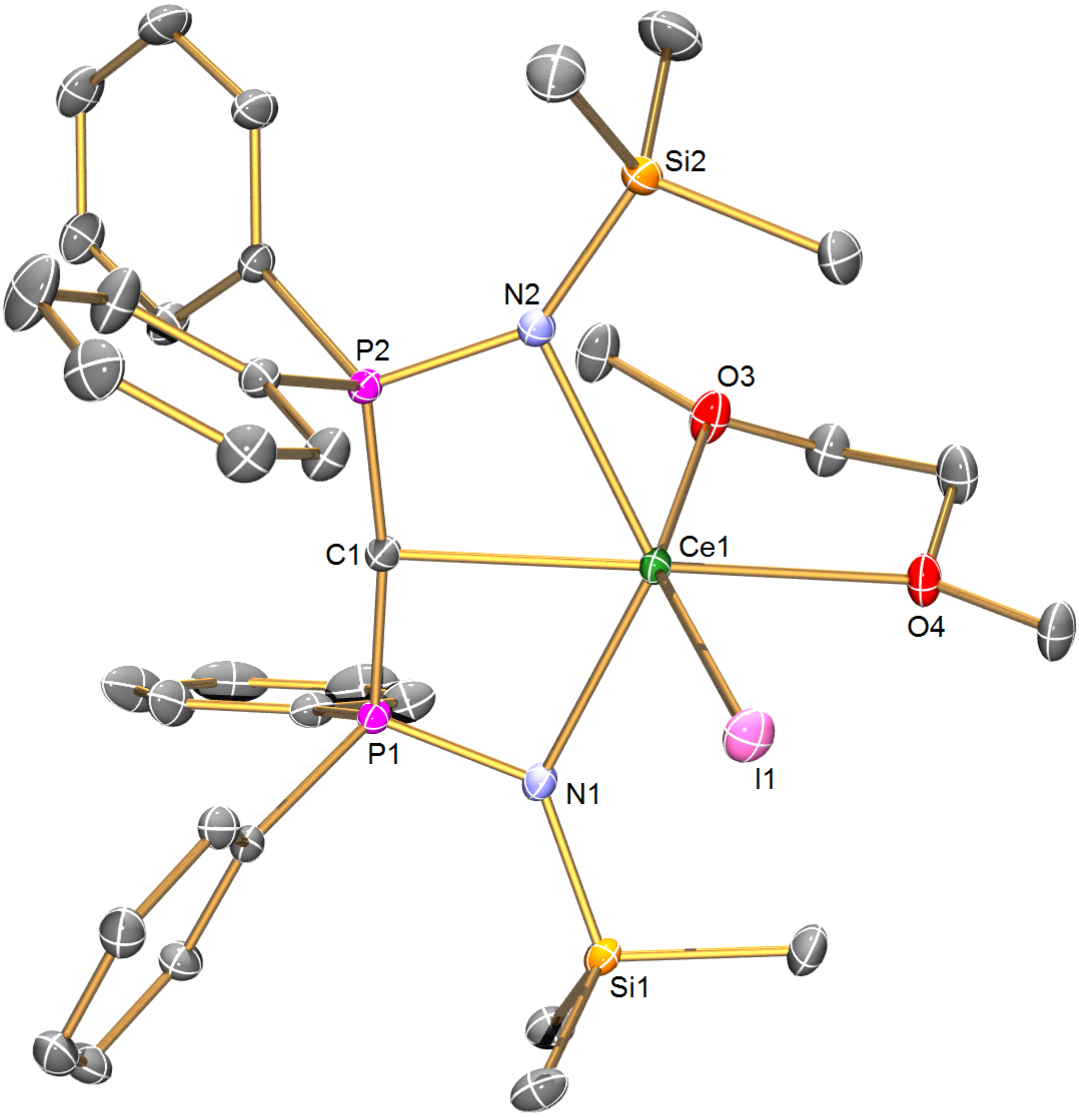

Figure S12. Solid state molecular structure of complex **5Ce** at 120K. Displacement ellipsoids are set at 50% probability and hydrogen atoms and lattice solvent are omitted for clarity. Selected bond lengths [Å] and angles [°]: Ce1-I1 3.1753(2), Ce1-N1 2.4585(17), Ce1-N2 2.4557(16), Ce1-O3 2.5697(15), Ce1-O4 2.6763(15), Ce1-C1 2.4772(19), P1-N1 1.6375(17), P1-C1 1.655(2), P2-N2 1.6244(17), P2-C1 1.657(2), N1-Ce1-I1 94.91(4), N1-Ce1-O3 101.98(6), N1-Ce1-O4 101.90(5), N1-Ce1-C1 64.56(6), N2-Ce1-I1 104.67(4), N2-Ce1-N1 128.35(5), N2-Ce1-O3 80.64(6), N2-Ce1-O4 122.82(5), N2-Ce1-C1 63.87(6), O3-Ce1-I1 153.47(4), O3-Ce1-O4 61.82(5), O4-Ce1-I1 94.92(3), C1-Ce1-I1 110.00(5), C1-Ce1-O3 95.77(6), C1-Ce1-O4 152.05(6), P1-C1-P2 171.57(14).

Complex 6Ce

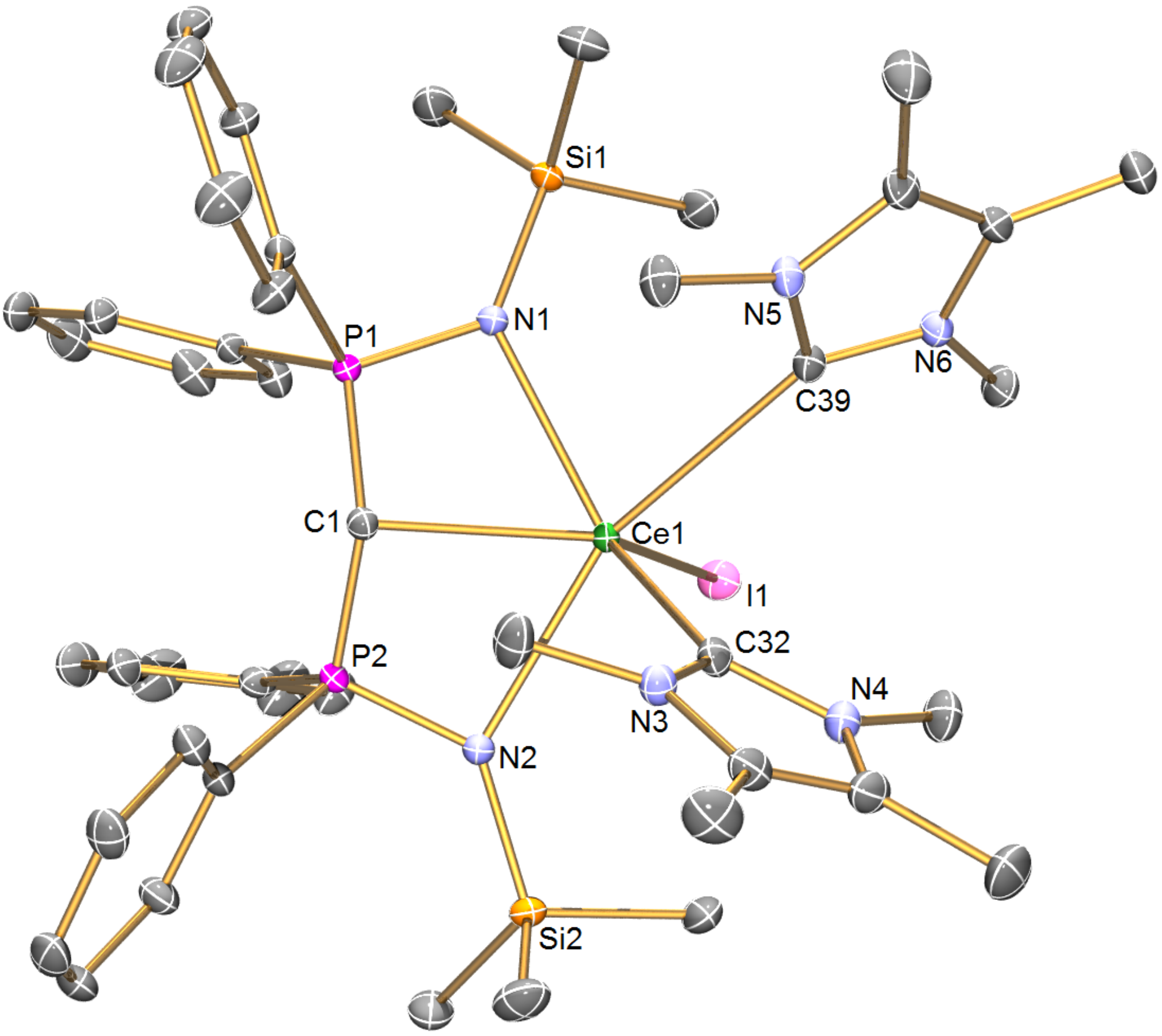

Figure S13. Solid state molecular structure of complex **6Ce** at 100K. Displacement ellipsoids are set at 50% probability and hydrogen atoms and lattice solvent are omitted for clarity. Selected bond lengths [Å] and angles [°]: Ce1-I1 3.2054(2), Ce1-N1 2.510(2), Ce1-N2 2.494(2), Ce1-C1 2.519(2), Ce1-C32 2.737(3), Ce1-C39 2.806(2), P1-N1 1.624(2), P1-C1 1.667(3), P2-N2 1.622(2), P2-C1 1.679(2), N1-Ce1-I1 98.36(5), N1-Ce1-C1 63.75(7), N1-Ce1-C32 132.22(7), N1-Ce1-C39 81.24(7), N2-Ce1-I1 93.16(5), N2-Ce1-N1 120.80(6), N2-Ce1-C1 63.76(7), N2-Ce1-C32 81.69(7), N2-Ce1-C39 154.07(8), C1-Ce1-I1 128.10(6), C1-Ce1-C32 99.50(8), C1-Ce1-C39 124.47(7), C32-Ce1-I1 123.70(5), C32-Ce1-C39 72.82(7), C39-Ce1-I1 97.02(5), P1-C1-P2 137.37(16).

Complex 4Np

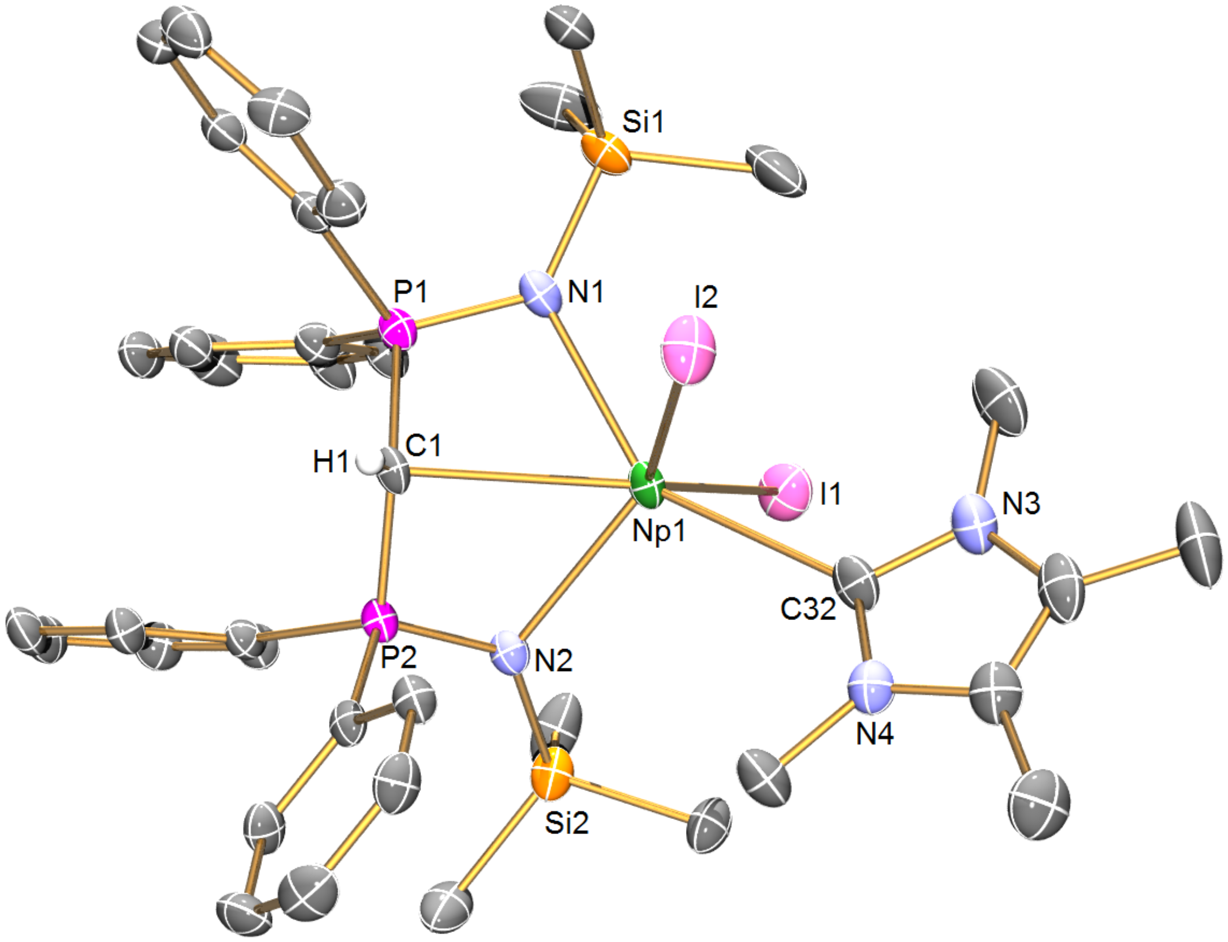

Figure S14. Solid state molecular structure of complex **4Np** at 100K. Displacement ellipsoids are set at 50% probability and non-methanide hydrogen atoms are omitted for clarity. Selected bond lengths [Å] and angles [°]: Np1-I1 3.0727(6), Np1-I2 3.1798(6), Np1-N1 2.423(6), Np1-N2 2.458(6), Np1-C1 2.753(7), Np1-C32 2.676(8), P1-N1 1.612(6), P1-C1 1.749(7), P2-N2 1.618(6), P2-C1 1.723(7), I1-Np1-I2 135.874(19), N1-Np1-I1 93.13(14), N1-Np1-I2 84.69(14), N1-Np1-N2 105.8(2), N1-Np1-C1 63.3(2), N1-Np1-C32 144.1(2), N2-Np1-I1 91.10(13), N2-Np1-I2 131.94(13), N2-Np1-C1 62.0(2), N2-Np1-C32 110.0(2), C1-Np1-I1 133.83(14), C1-Np1-I2 83.80(14), C32-Np1-I1 88.22(16), C32-Np1-I2 70.04(17), C32-Np1-C1 134.6(2), P1-C1-P2 128.6(4).

Complex 5Np

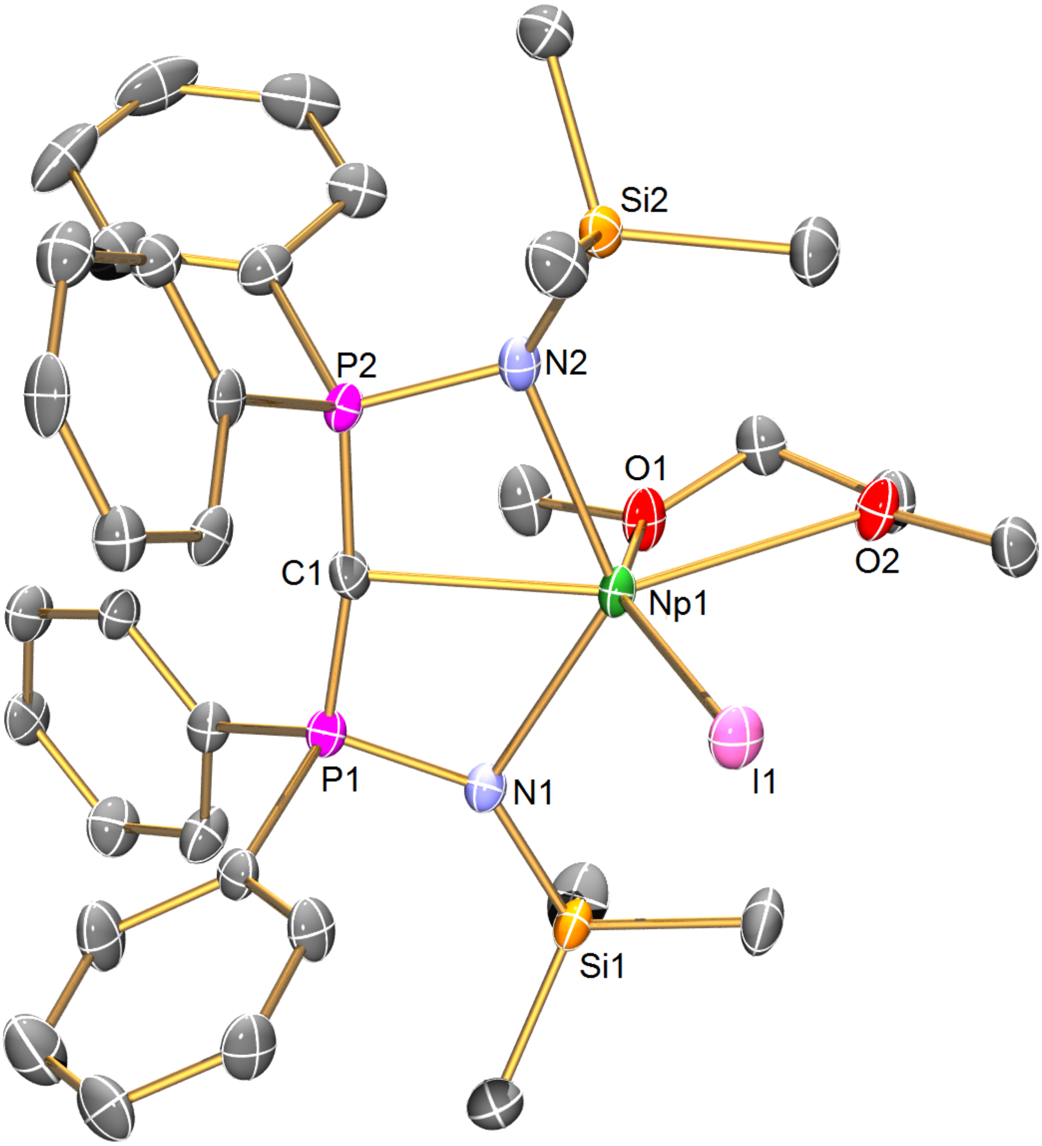

Figure S15. Solid state molecular structure of complex **5Np** at 100K. Displacement ellipsoids are set at 50% probability and hydrogen atoms and lattice solvent are omitted for clarity. Selected bond lengths [Å] and angles [°]: Np1-I1 3.1065(5), Np1-O1 2.524(5), Np1-O2 2.636(5), Np1-N1 2.431(6), Np1-N2 2.414(6), Np1-C1 2.425(7), P1-N1 1.602(6), P1-C1 1.627(7), P2-N2 1.631(6), P2-C1 1.652(7), O1-Np1-I1 153.90(12), O1-Np1-O2 61.84(16), O2-Np1-I1 95.27(10), N1-Np1-I1 103.57(13), N1-Np1-O1 81.28(18), N1-Np1-O2 122.42(17), N2-Np1-I1 95.23(12), N2-Np1-O1 101.53(18), N2-Np1-O2 102.19(17), N2-Np1-N1 128.81(18), N2-Np1-C1 65.2(2), C1-Np1-I1 109.31(17), C1-Np1-O1 95.9(2), C1-Np1-O2 152.9(2), C1-Np1-N1 63.6(2), P1-C1-P2 170.4(5).

Complex 6Np

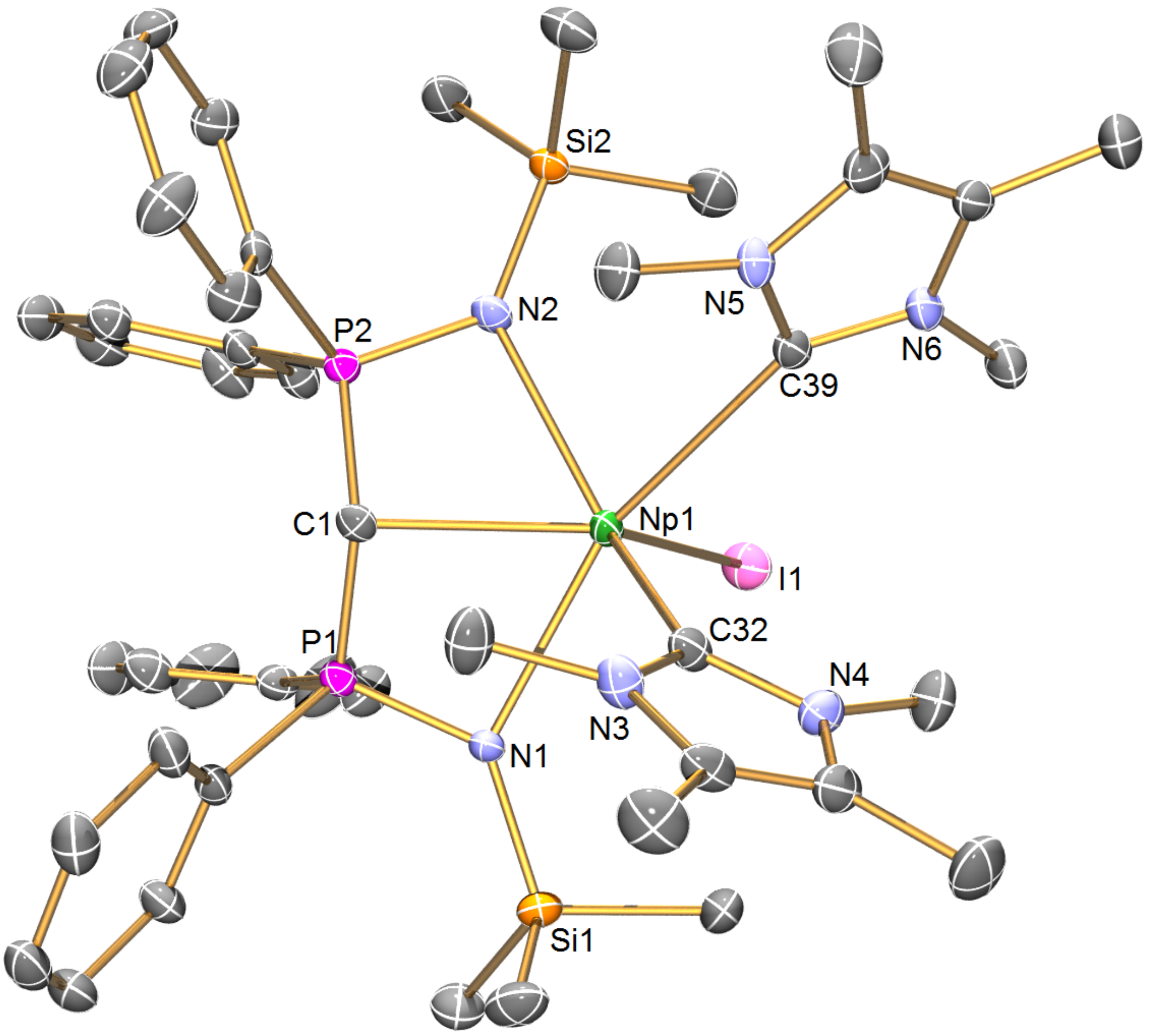

Figure S16. Solid state molecular structure of complex **6Np** at 120K. Displacement ellipsoids are set at 50% probability and hydrogen atoms and lattice solvent are omitted for clarity. Selected bond lengths [Å] and angles [°]: Np1-I1 3.1571(4), Np1-N1 2.485(4), Np1-N2 2.492(5), Np1-C1 2.490(6), Np1-C32 2.677(5), Np1-C39 2.751(6), P1-N1 1.620(5), P1-C1 1.675(6), P2-N2 1.614(5), P2-C1 1.671(5), N1-Np1-I1 91.83(10), N1-Np1-N2 120.88(15), N1-Np1-C1 64.04(17), N1-Np1-C32 81.36(16), N1-Np1-C39 154.19(15), N2-Np1-I1 98.08(10), N2-Np1-C32 132.67(17), N2-Np1-C39 81.04(16), C1-Np1-I1 127.54(12), C1-Np1-N2 64.08(16), C1-Np1-C32 98.69(17), C1-Np1-C39 123.63(17), C32-Np1-I1 124.26(13), C32-Np1-C39 73.21(17), C39-Np1-I1 98.69(11), P1-C1-P2 136.5(3).

S5. UV-vis-NIR spectra

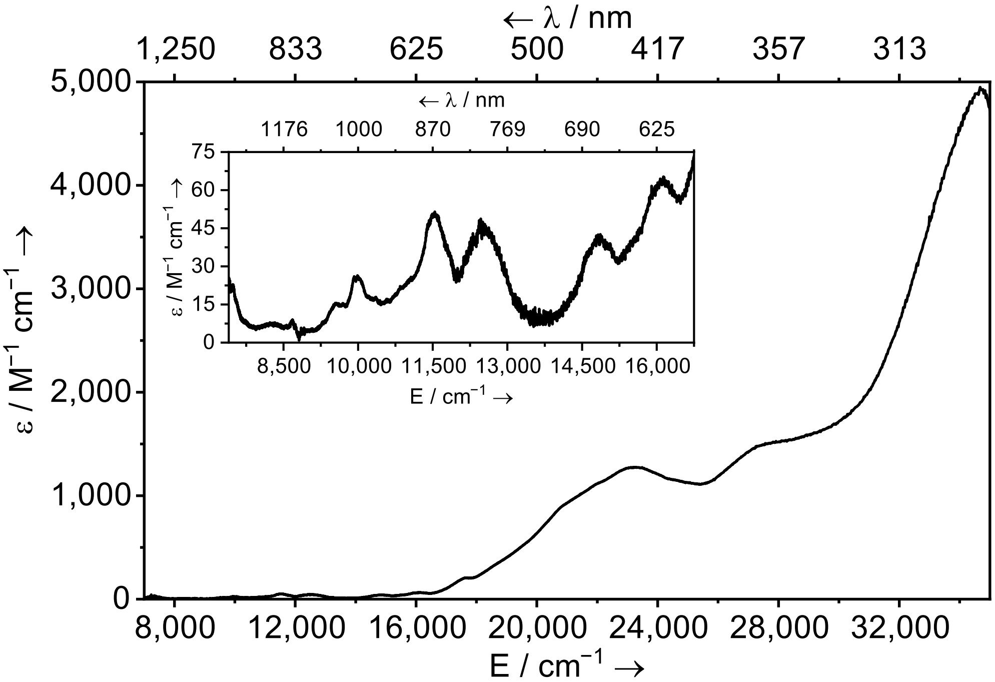

Figure S17. Solution UV-vis-NIR spectrum of **4Np** (0.49 mM) in toluene shown between 7,000–35,000 cm^{-1} (1,429–286 nm) at ambient temperature.

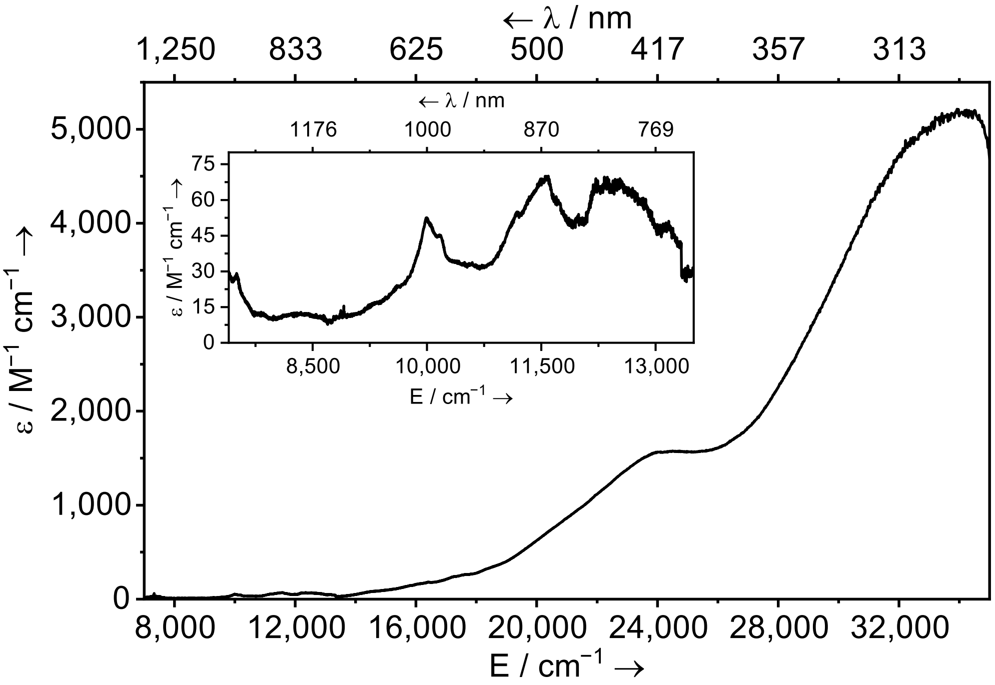

Figure S18. Solution UV-vis-NIR spectrum of **5Np** (0.51 mM) in toluene shown between 7,000–35,000 cm^{-1} (1,429–286 nm) at ambient temperature.

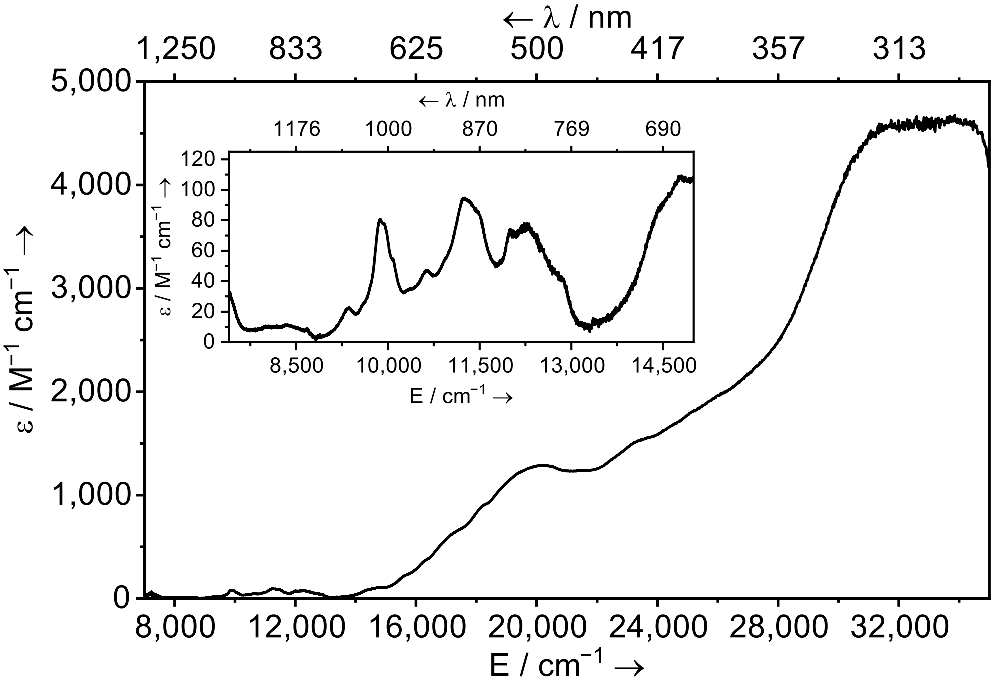

Figure S19. Solution UV-vis-NIR spectrum of **6Np** (0.58 mM) in toluene shown between 7,000–35,000 cm^{-1} (1,429–286 nm) at ambient temperature.

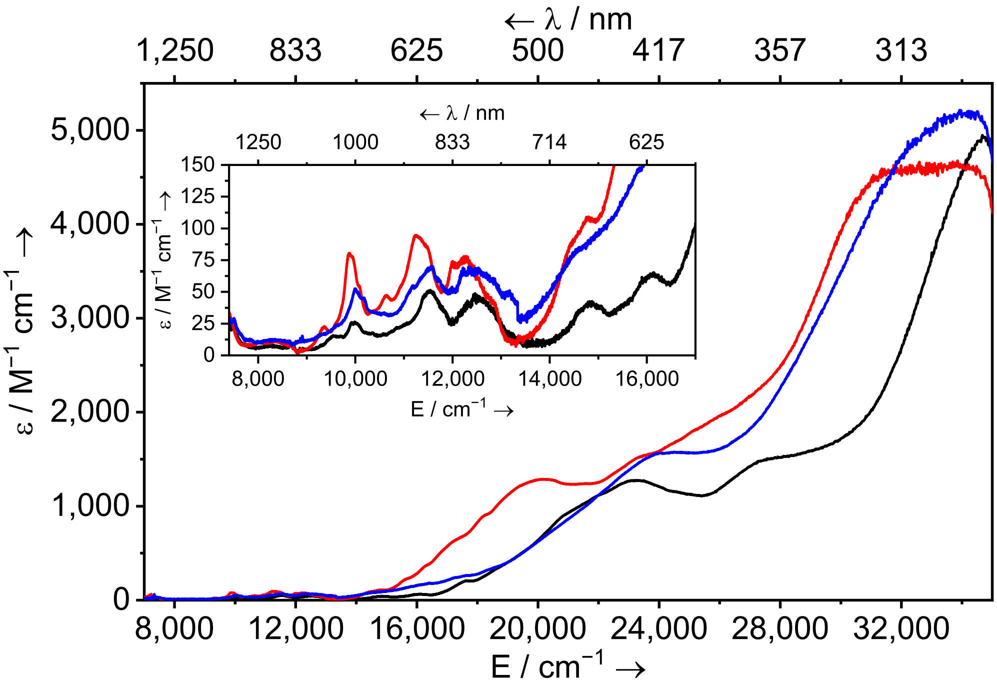

Figure S20. Comparison of solution UV-vis-NIR spectra of **4Np** (black line, 0.49 mM), **5Np** (blue line, 0.51 mM), and **6Np** (red line, 0.58 mM), all in toluene shown between 7,000–35,000 cm^{-1} (1,429–286 nm) at ambient temperature.

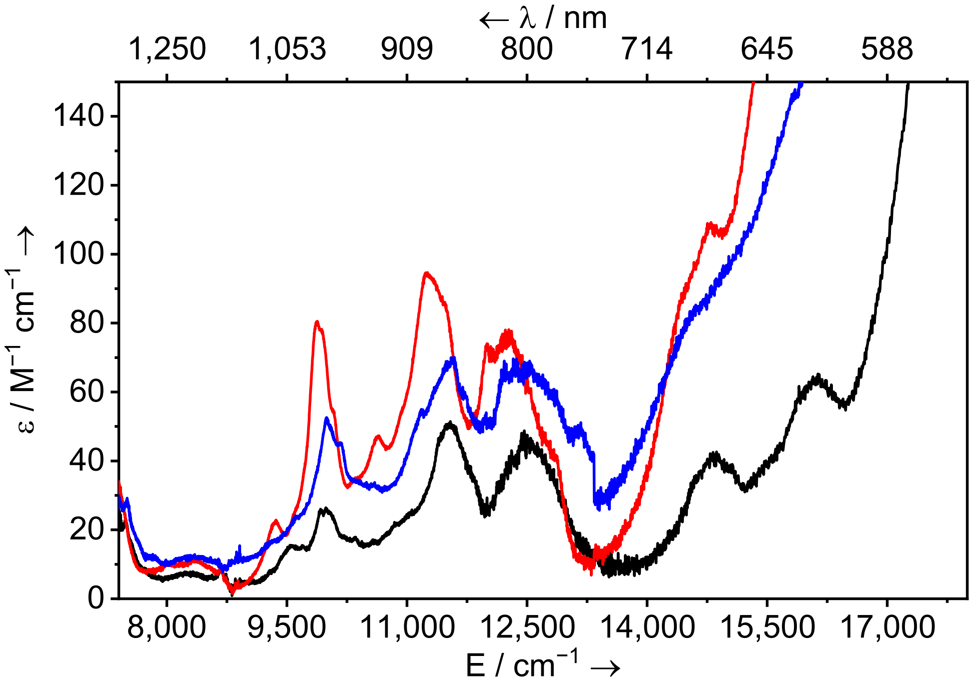

Figure S21. The same spectra as Figure S20 (**4Np** black line, **5Np** blue line, and **6Np** red line), but shown in the region between 7,400–18,000 cm^{-1} (1,351–556 nm) – the region typically associated with *f*→*f* transitions (5*f*→5*f* here).

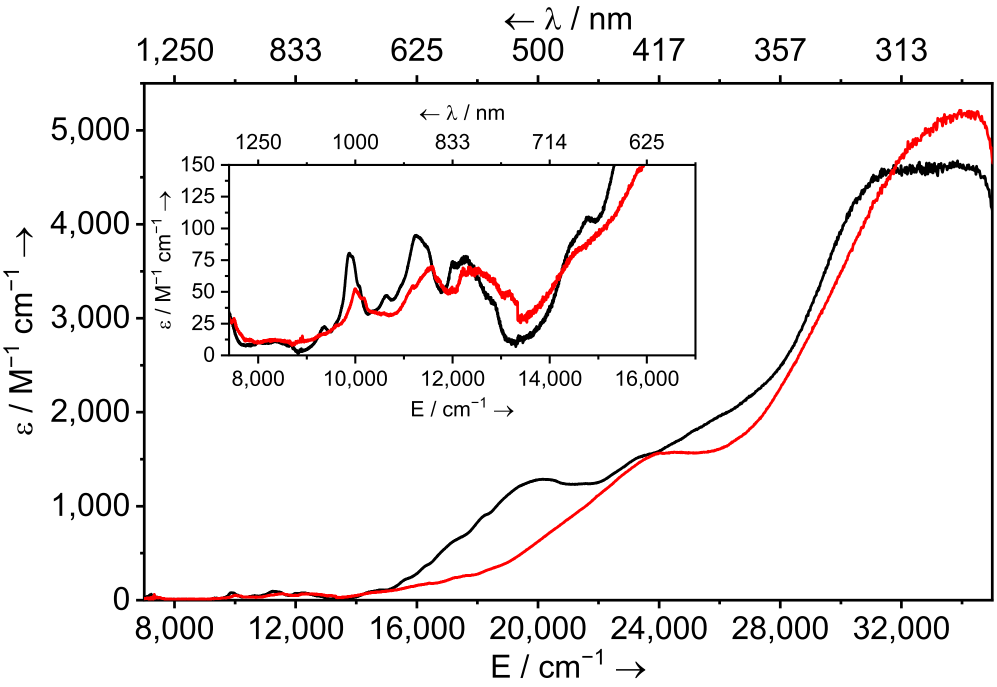

Figure S22. Comprison of solution UV-vis-NIR spectra of **5Np** (black line, 0.51 mM) and **6Np** (red line, 0.58 mM) – the two $(BIPM^{TMS})^{2-}$ complexes, all in toluene shown between 7,000–35,000 cm^{-1} (1,429–286 nm) at ambient temperature.

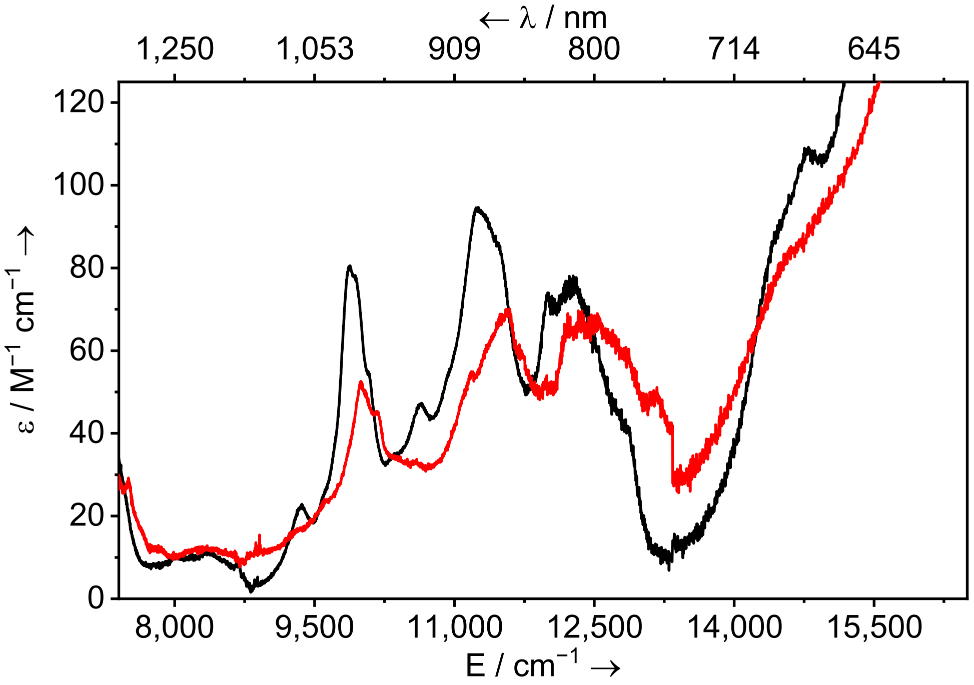

Figure S23. The same spectra as Figure S22 (**5Np** black line and **6Np** red line), but shown in the region between 7,400–16,500 cm^{-1} (1,351–606 nm) – the region typically associated with *f*→*f* transitions (5*f*→5*f* here).

S6. NMR spectra

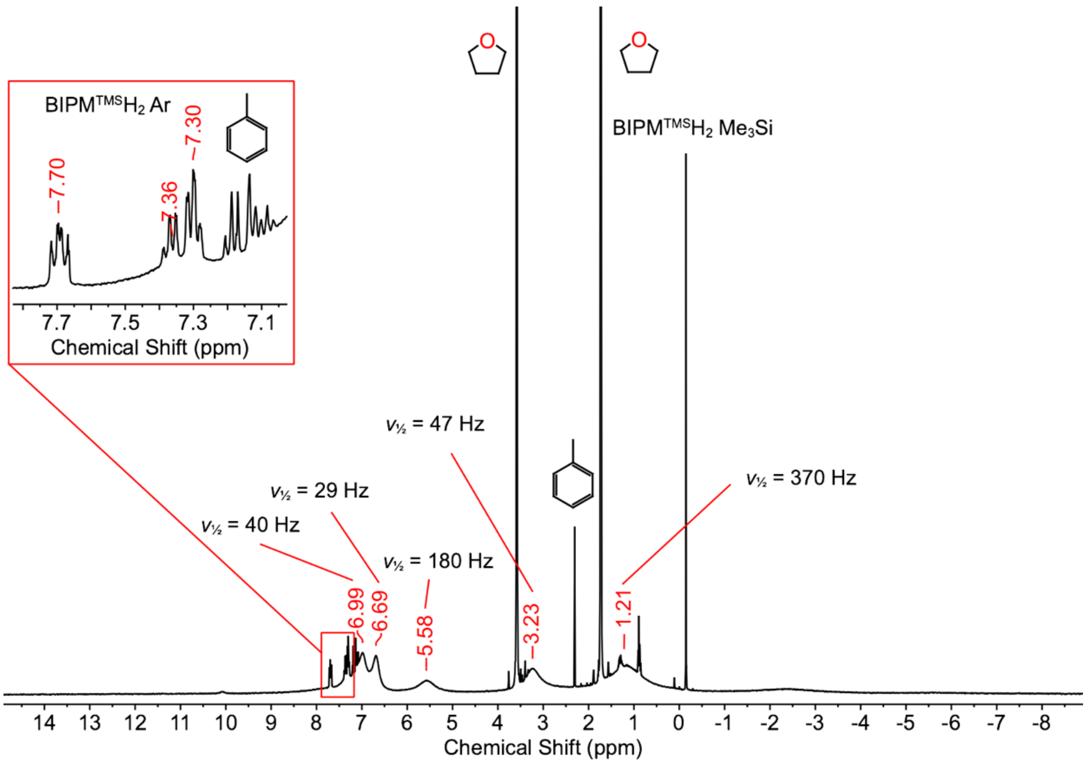

Figure S24. ^{1}H NMR spectrum of **4Ce** in d_8-THF.

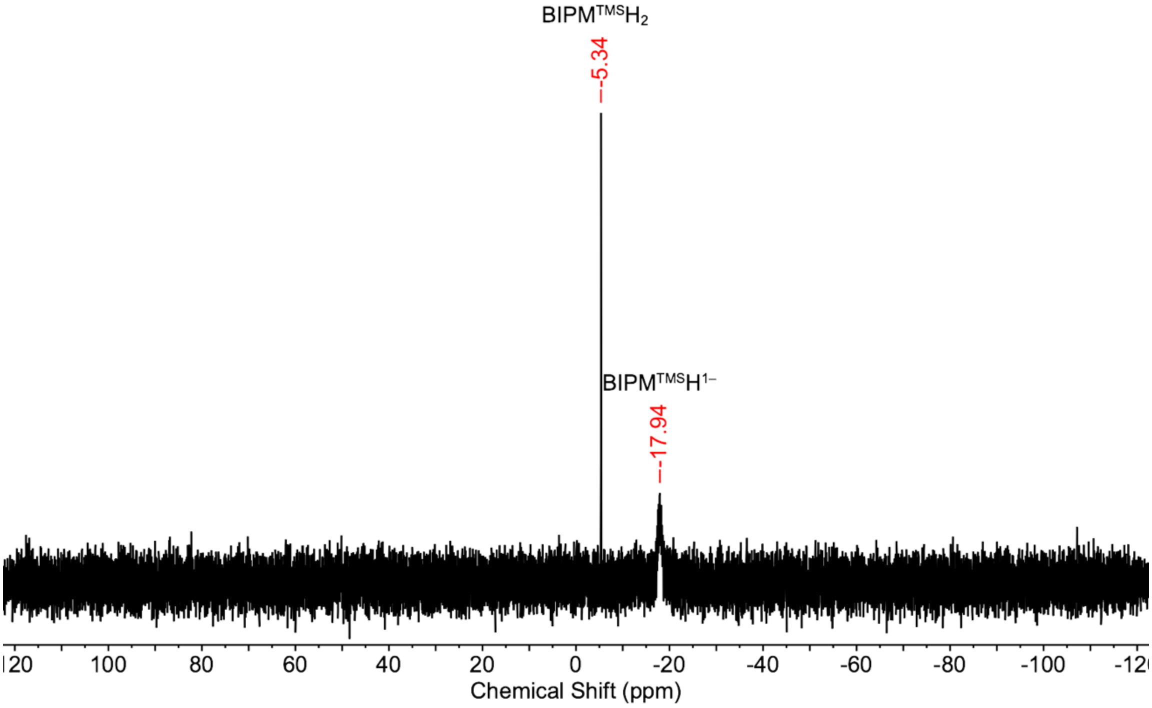

Figure S25. $^{31}P\{^1H\}$ NMR spectrum of **4Ce** in d_8-THF. The BIPM^{TMS}H$_2$ peak is from a small amount of decomposition during sample preparation.

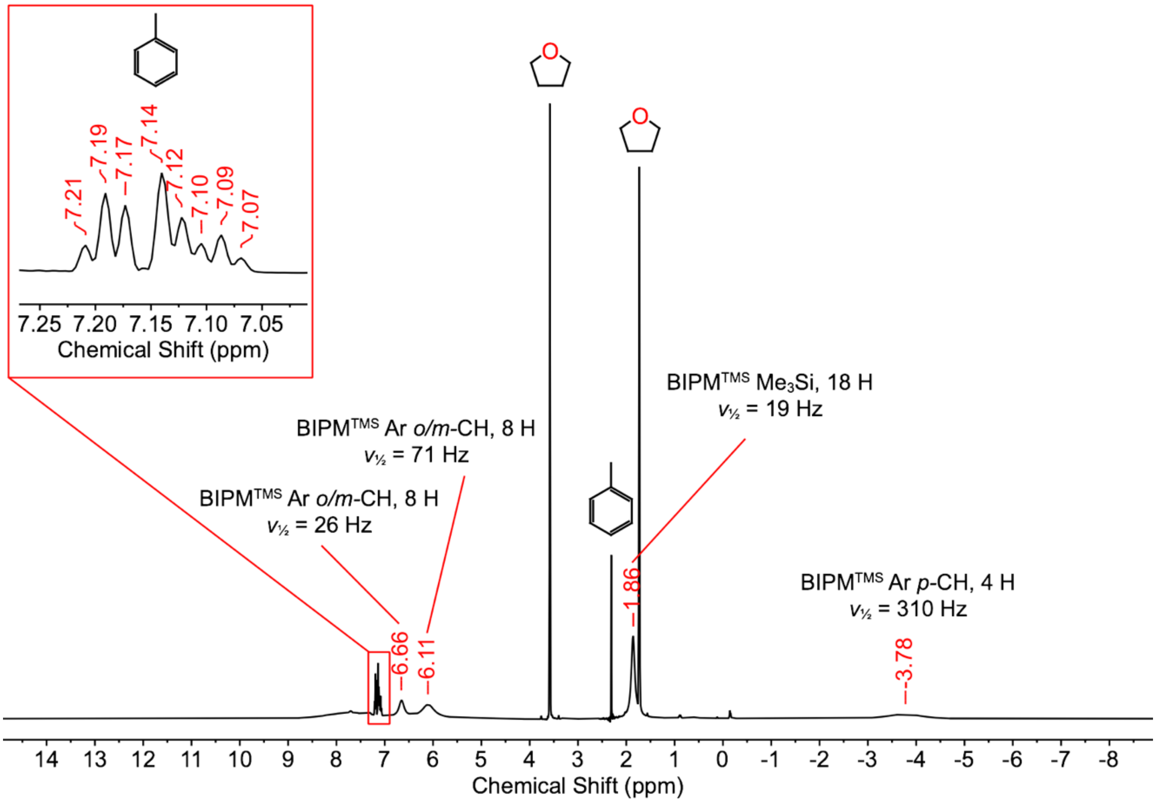

Figure S26. ^1H NMR spectrum of **6Ce** in d_8-THF.

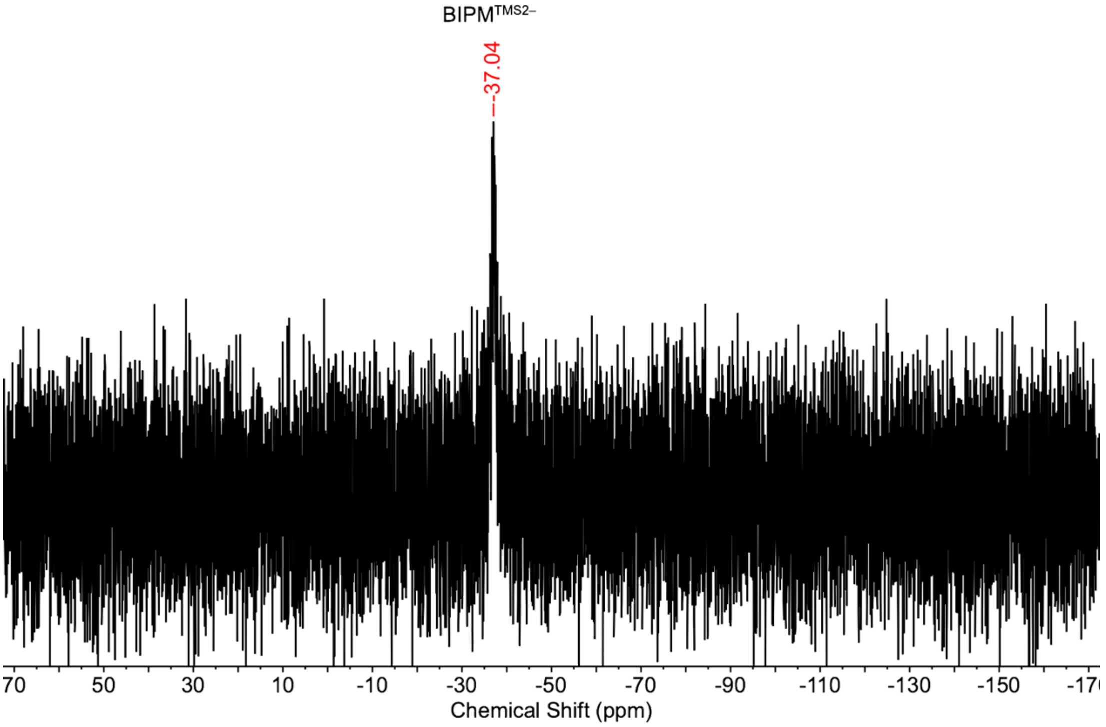

Figure S27. $^{31}P\{^1H\}$ NMR spectrum of **6Ce** in d_8-THF.

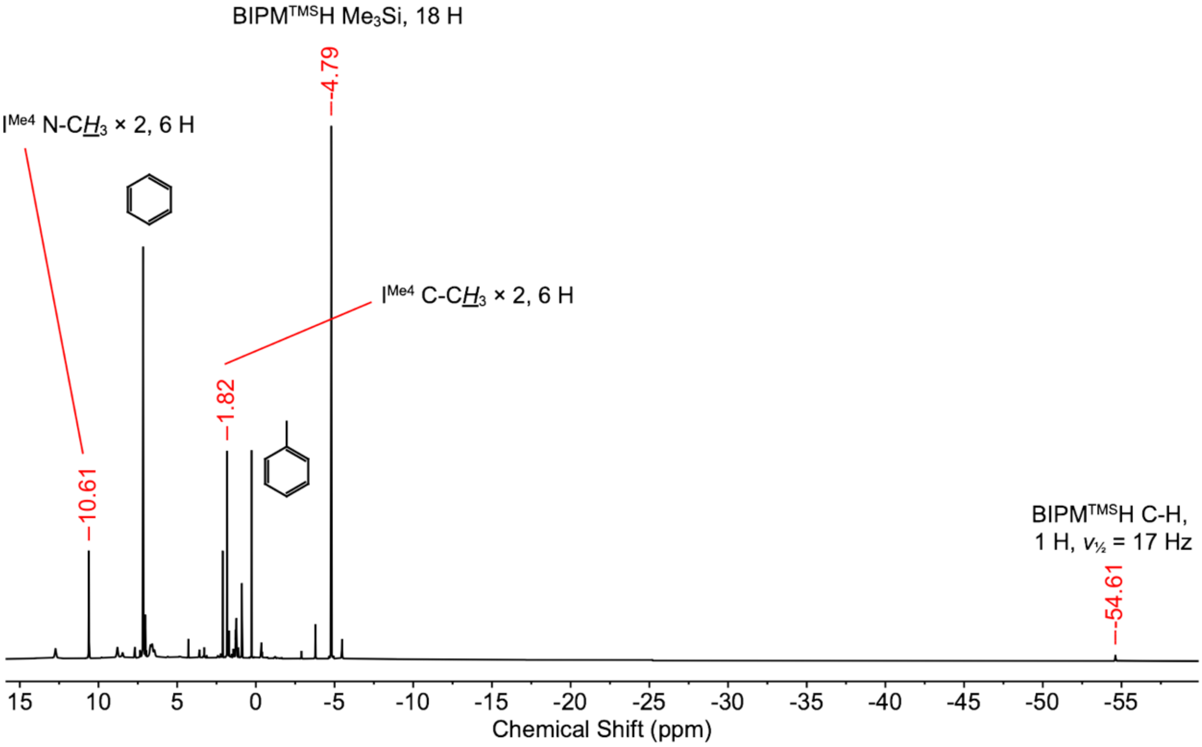

Figure S28. ^1H NMR spectrum of **4Np** in d_6-benzene.

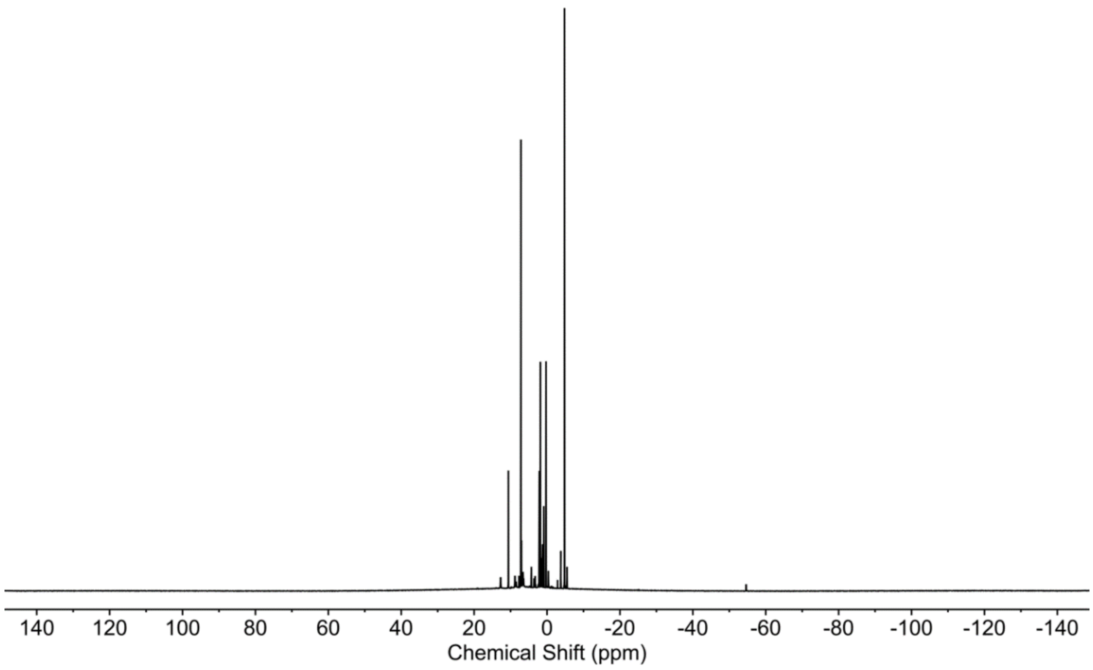

Figure S29. ^{1}H NMR spectrum of **4Np** in d_6-benzene, showing the full spectral window collected.

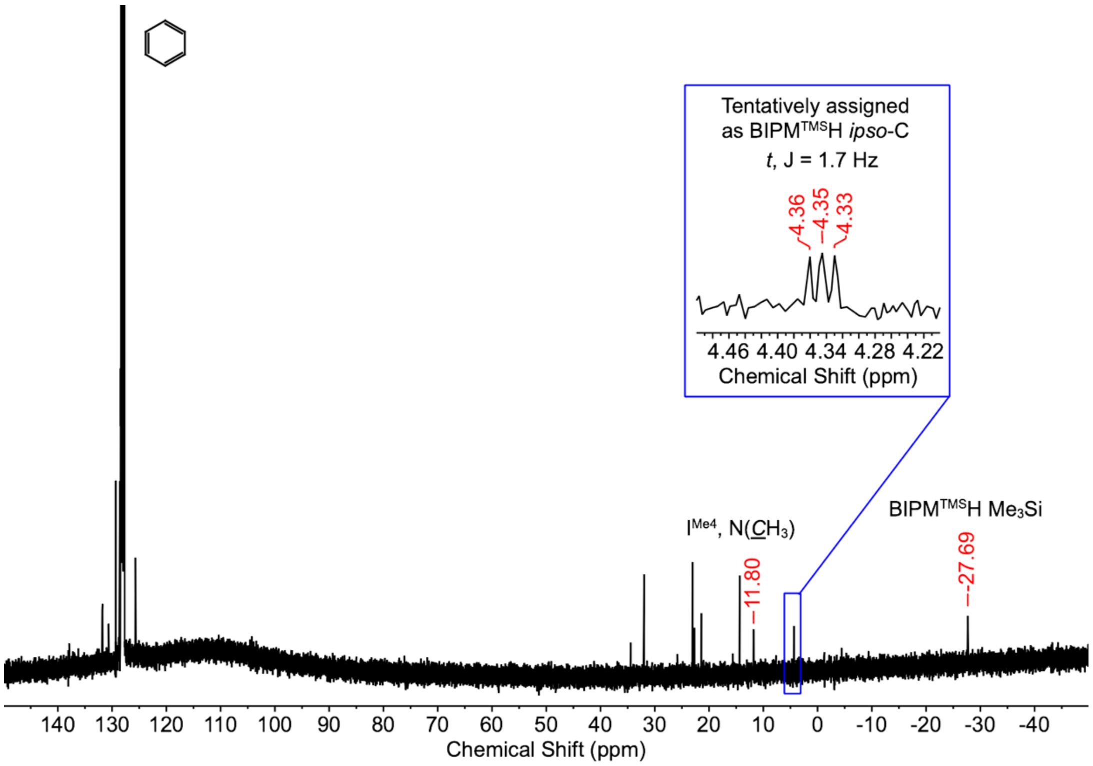

Figure S30. ^{13}C{^{1}H} NMR spectrum of **4Np** in d_6-benzene.

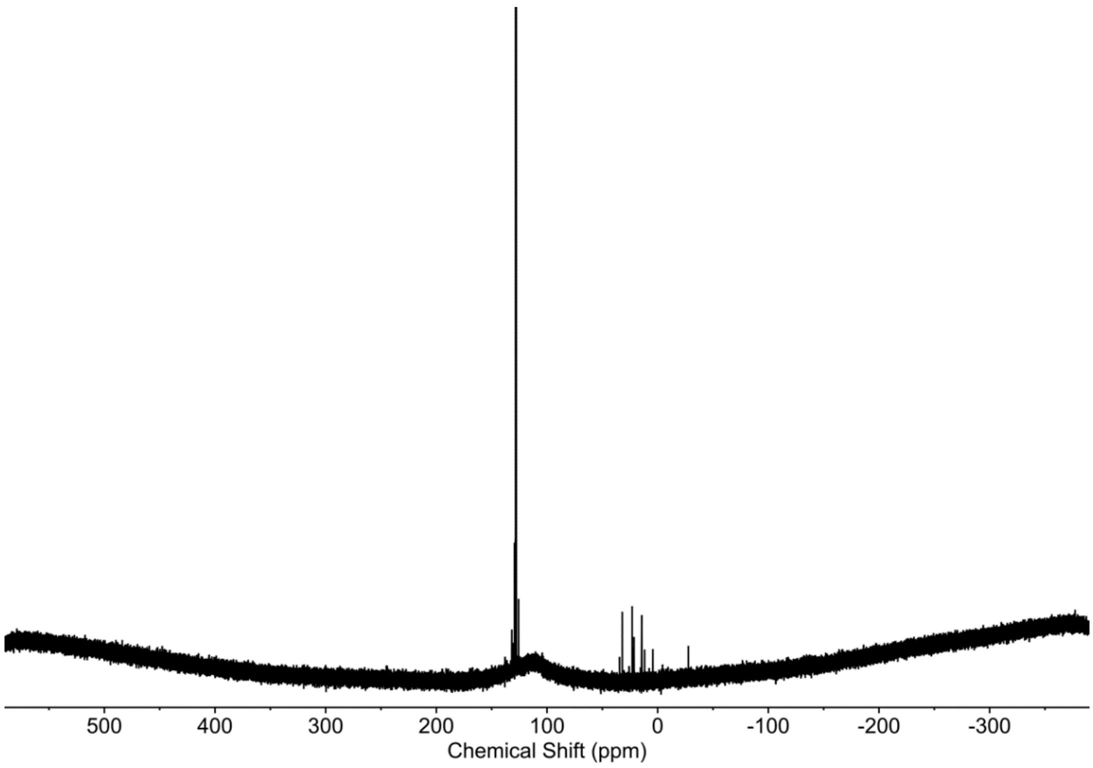

Figure S31. $^{13}C\{^{1}H\}$ NMR spectrum of **4Np** in d_6-benzene, showing the full spectral window collected.

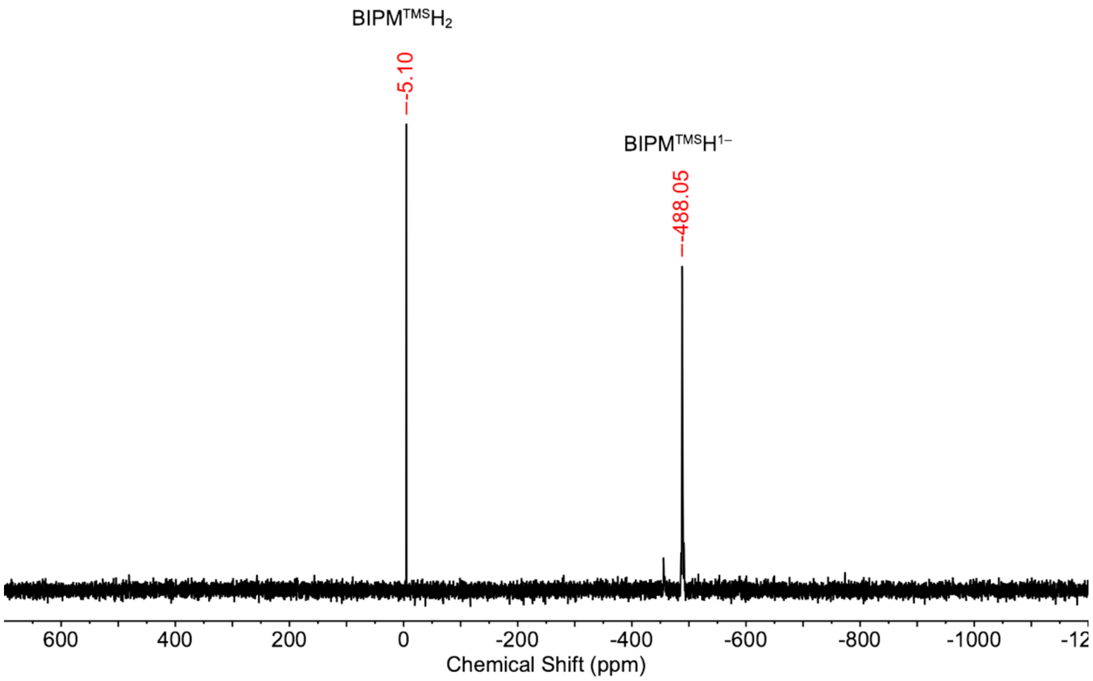

Figure S32. $^{31}P\{^{1}H\}$ NMR spectrum of **4Np** in d_6-benzene. The BIPM^{TMS}H$_2$ peak is likely from a small amount of decomposition given the limitations of our sample containment procedure.

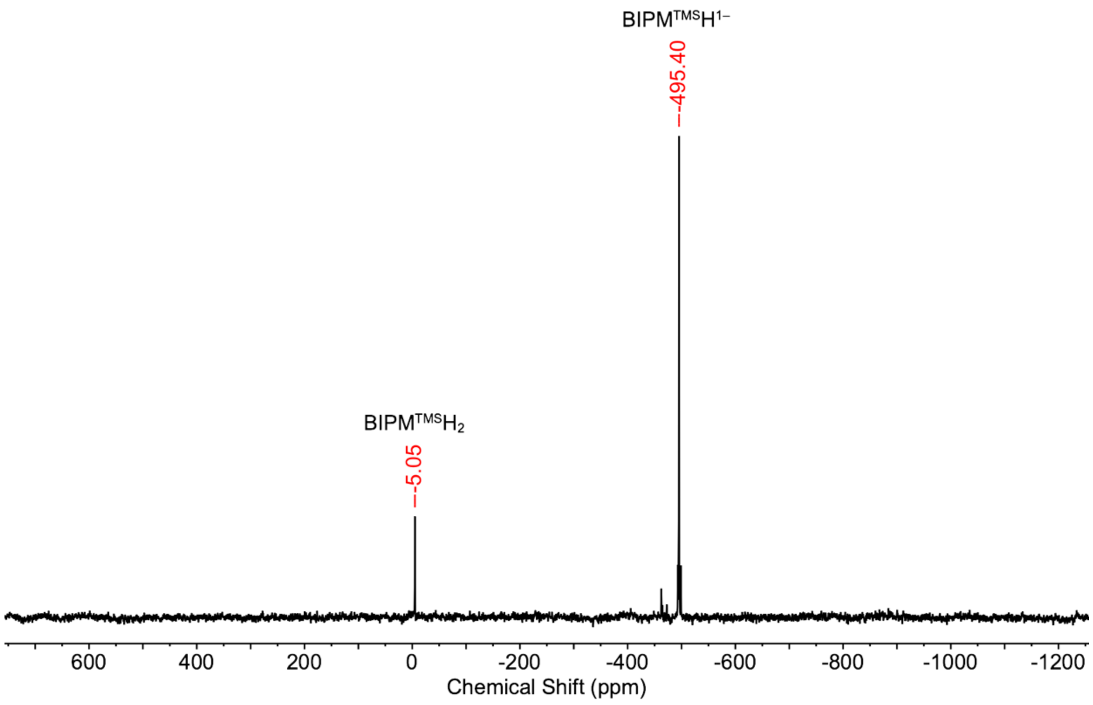

Figure S33. ^{31}P NMR spectrum of **4Np** in d_6-benzene. The BIPM^{TMS}H$_2$ peak is likely from a small amount of decomposition given the limitations of our sample containment procedure.

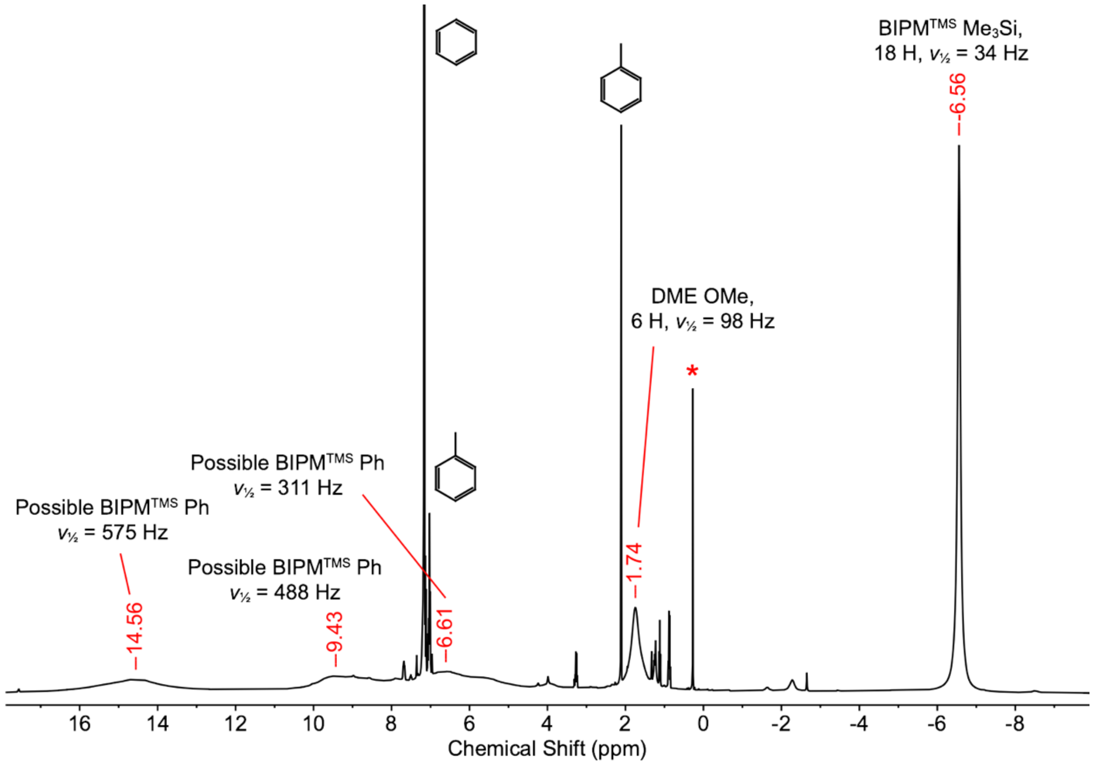

Figure S34. ^{1}H NMR spectrum of **5Np** in d_6-benzene. The BIPM^{TMS}H$_2$ peak (*) is likely from a small amount of decomposition given the limitations of our sample containment procedure.

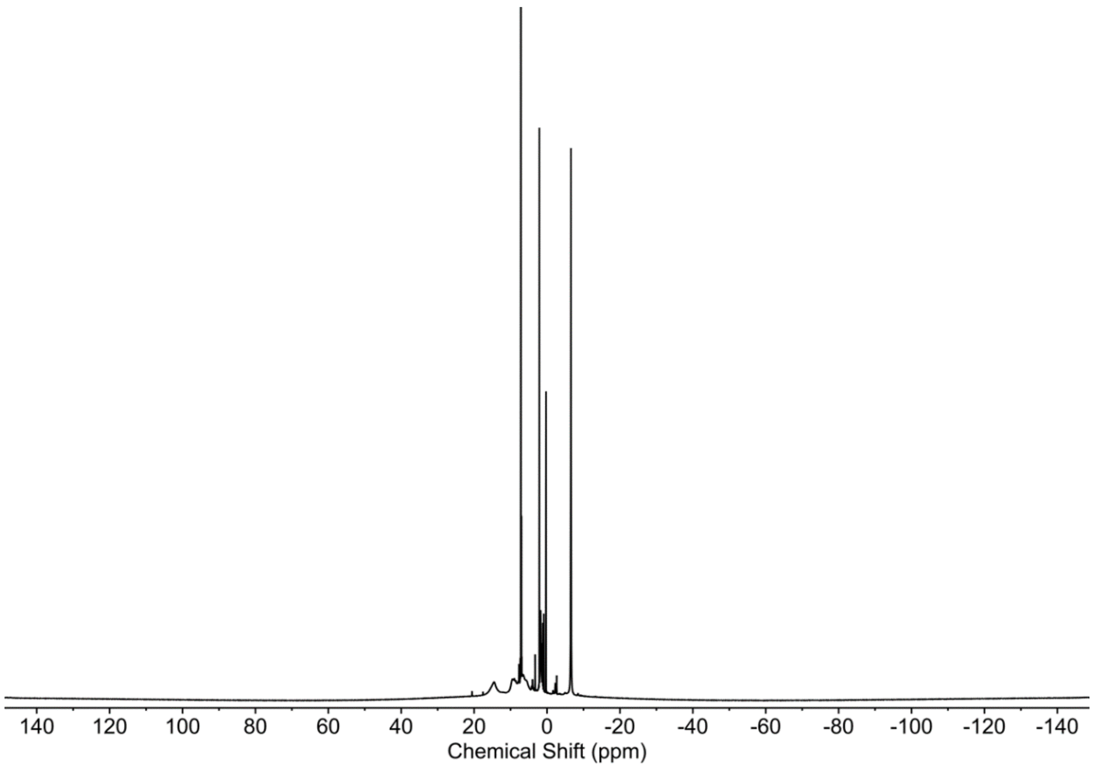

Figure S35. ^{1}H NMR spectrum of **5Np** in d_6-benzene, showing the full spectral window collected.

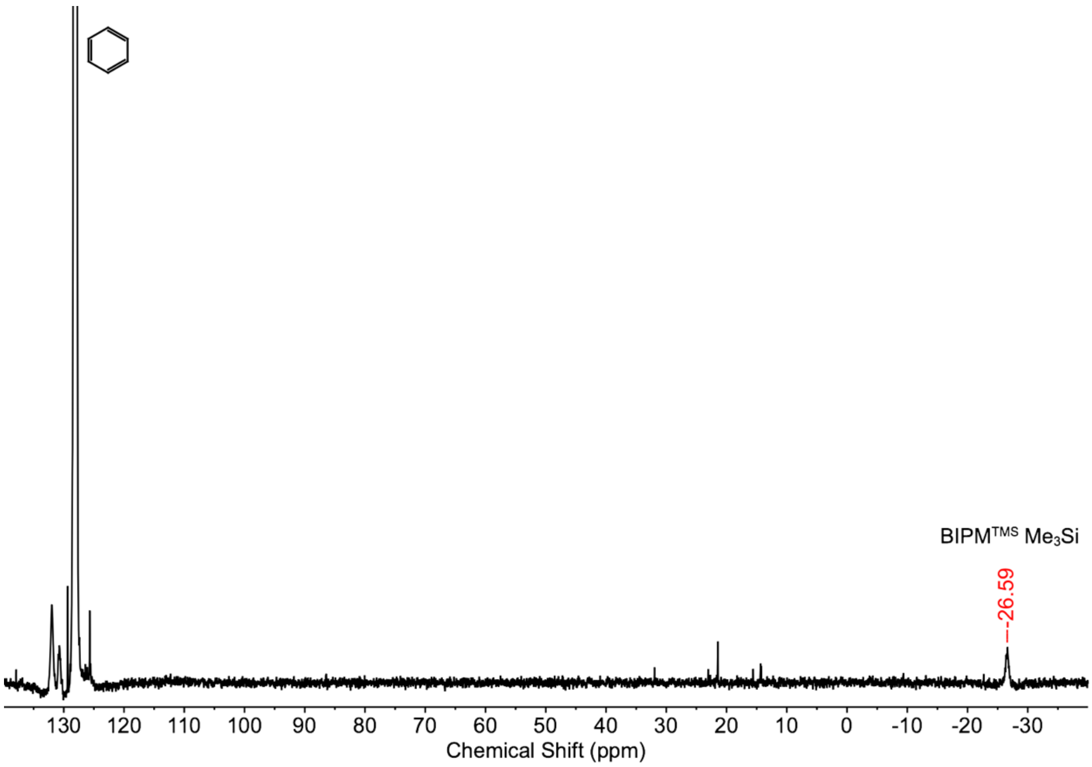

Figure S36. $^{13}C\{^{1}H\}$ NMR spectrum of **5Np** in d_6-benzene. No peaks readily attributable to the *ipso*-C on the $\{BIPM^{TMS}\}^{2-}$ ligand could be located, or any other peaks not due to solvents or $BIPM^{TMS}H_2$.

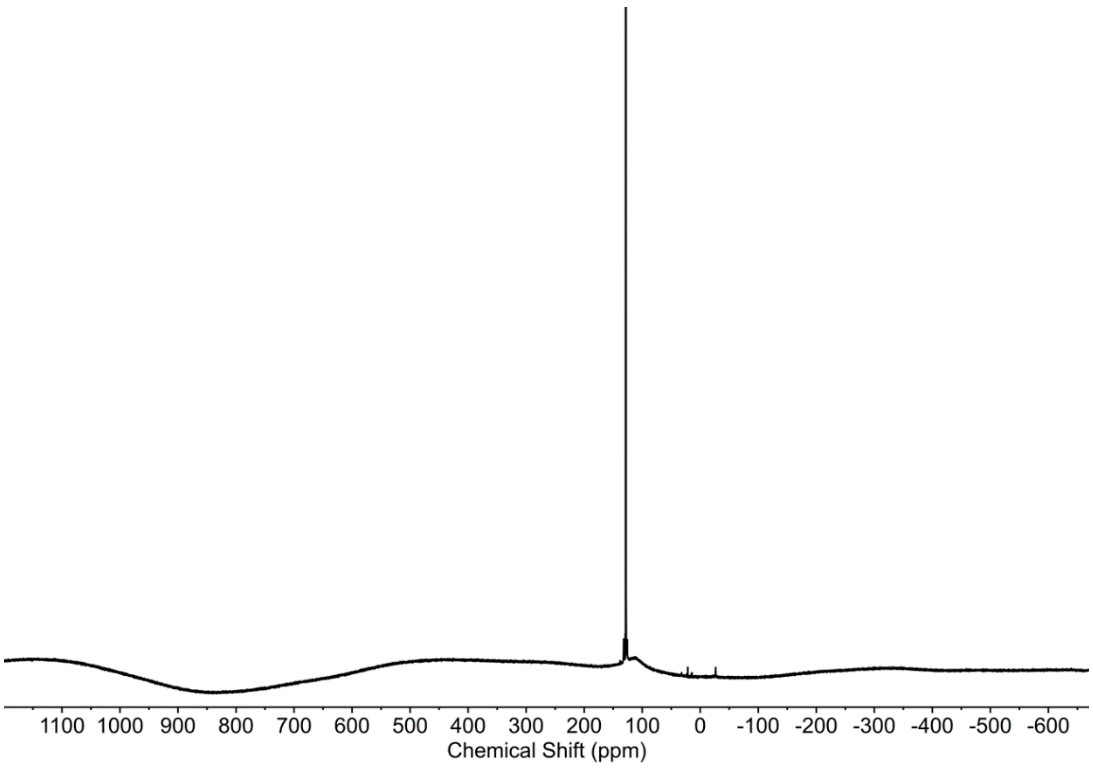

Figure S37. $^{13}C\{^1H\}$ NMR spectrum of **5Np** in d_6-benzene, showing the full spectral window collected.

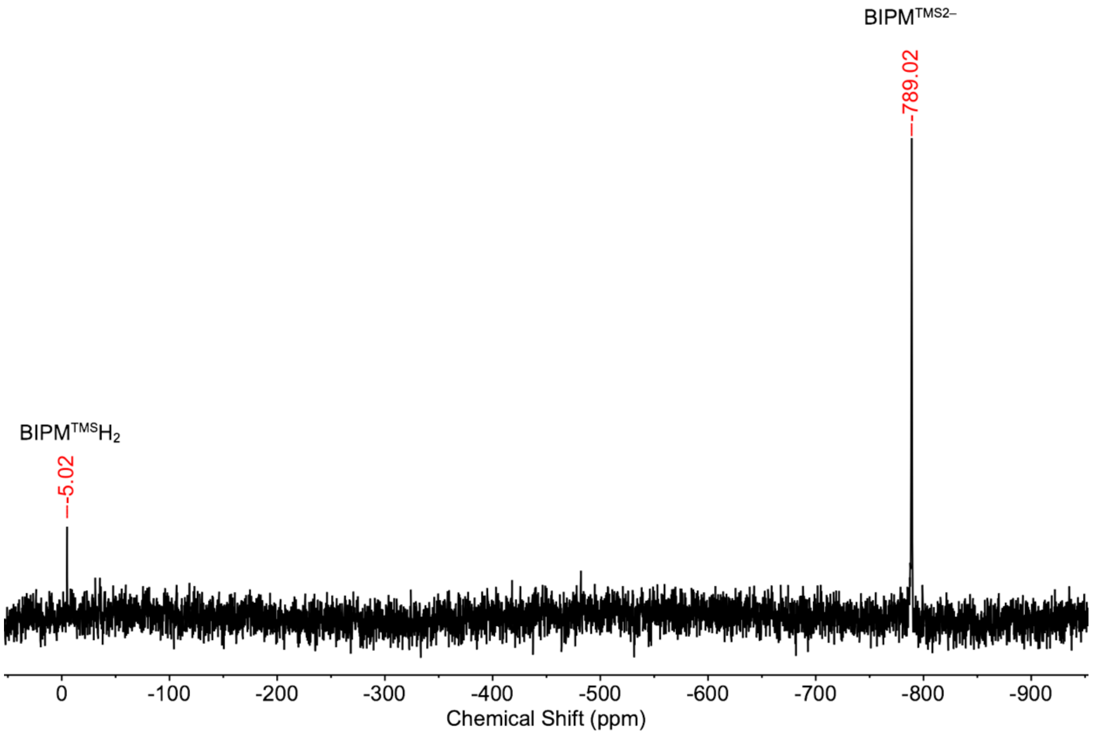

Figure S38. $^{31}P\{^1H\}$ NMR spectrum of **5Np** in d_6-benzene. The H_2BIPM^{TMS} peak is likely from a small amount of decomposition given the limitations of our sample containment procedure.

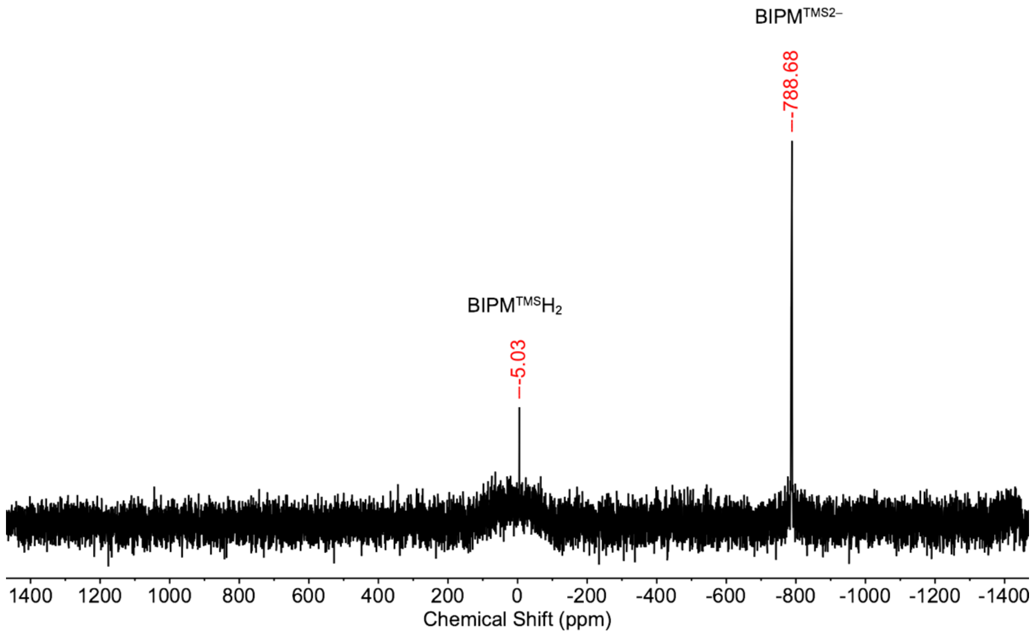

Figure S39. ^{31}P NMR spectrum of **5Np** in d_6-benzene. The BIPM$^{TMS}H_2$ peak is likely from a small amount of decomposition given the limitations of our sample containment procedure.

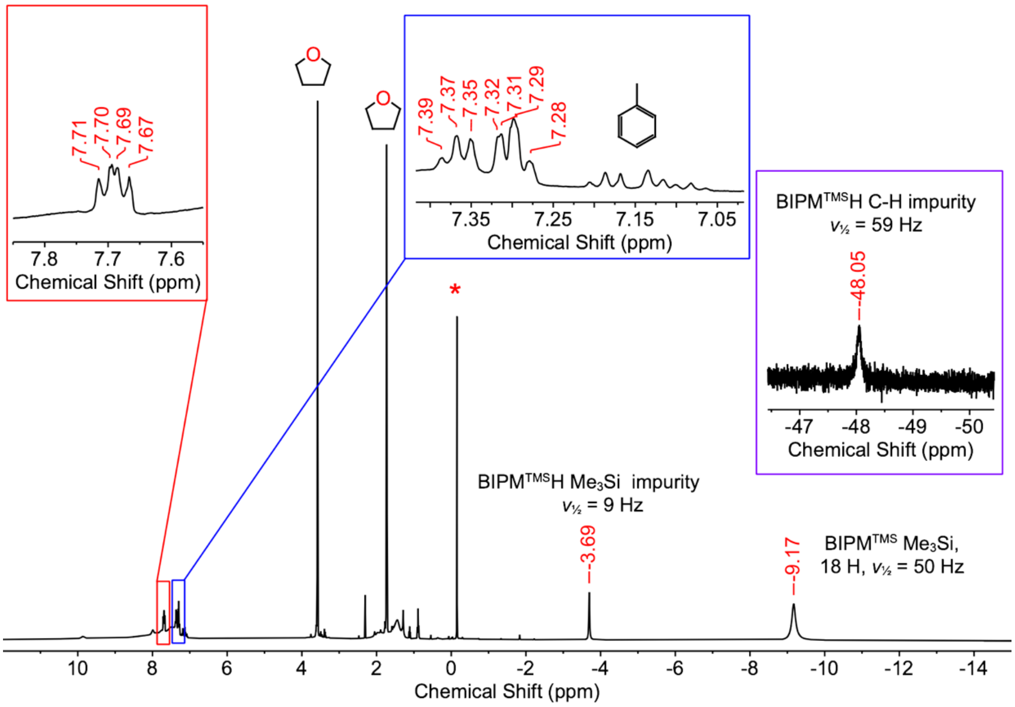

Figure S40. ^{1}H NMR spectrum of **6Np** in d_8-THF. The BIPM$^{TMS}H_2$ and BIPM^{TMS}H peaks (see below) are likely from a small amount of decomposition given the limitations of our sample containment procedure combined with the need to heat the sample of **6Np** strongly to dissolve it in d_8-THF – the complex is essentially insoluble in d_6-benzene.

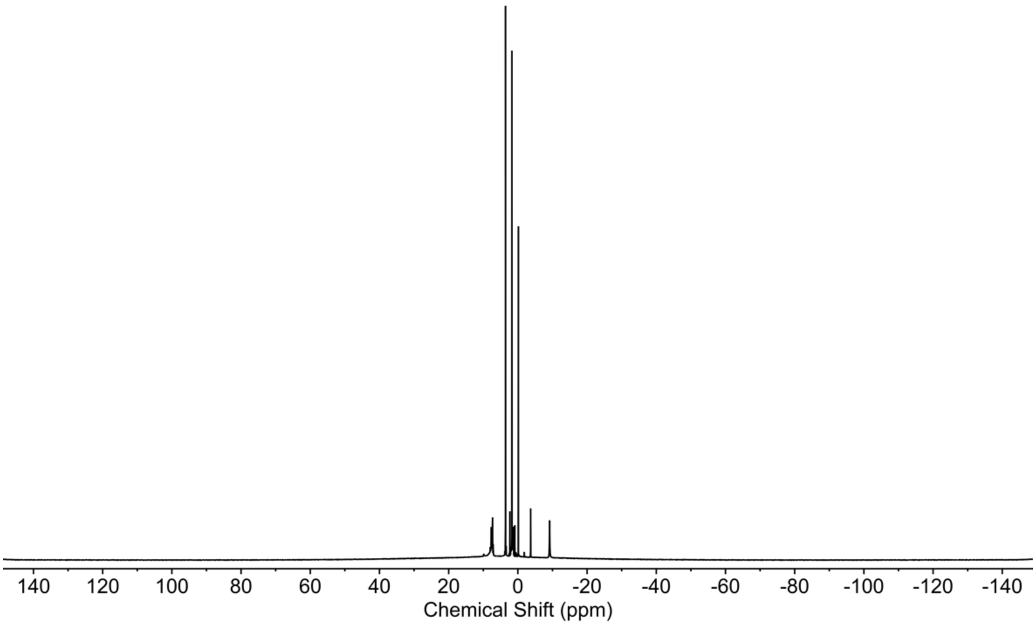

Figure S41. ^{1}H NMR spectrum of **6Np** in *d*$_8$-THF, showing the full spectral window collected.

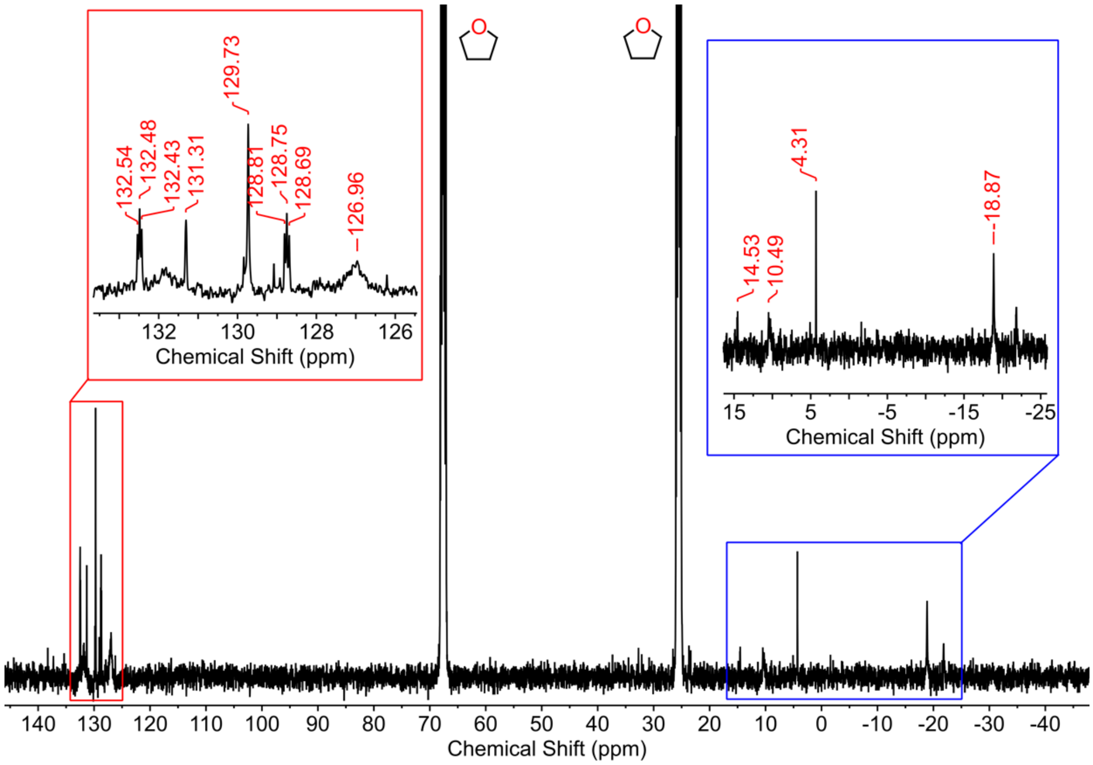

Figure S42. ^{13}C{^{1}H} NMR spectrum of **6Np** in *d*$_8$-THF. No peaks definitively assignable to **6Np** could be identified.

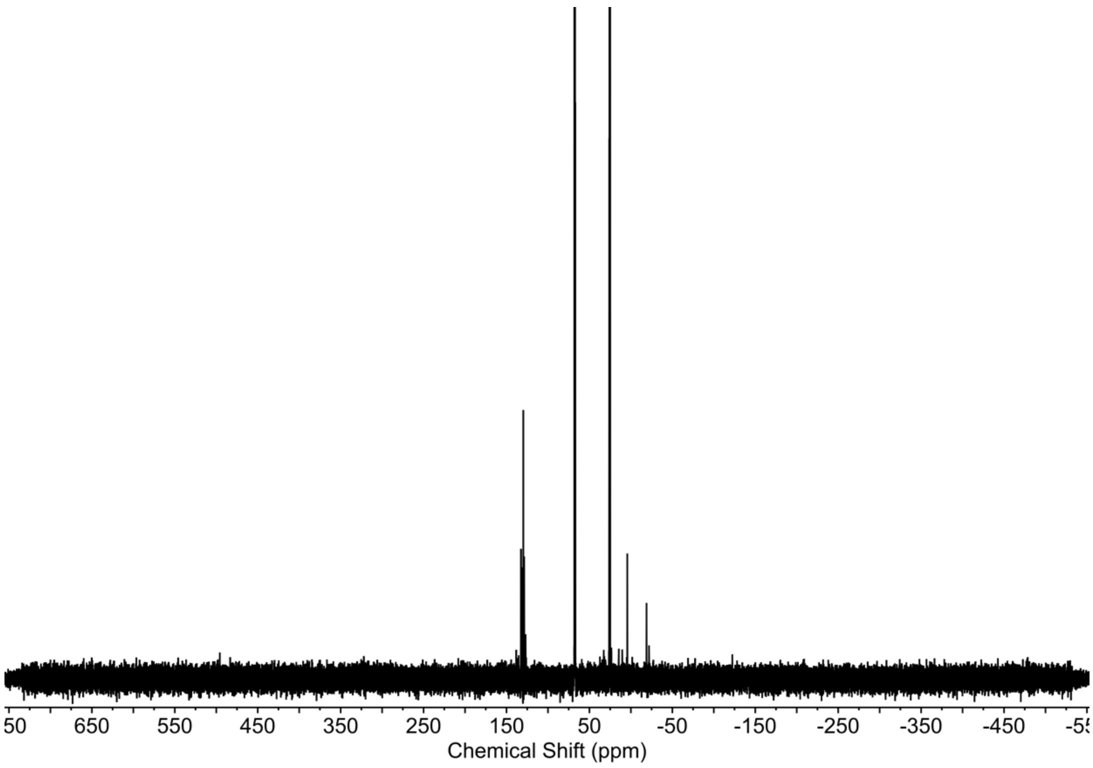

Figure S43. $^{13}C\{^1H\}$ NMR spectrum of **6Np** in d_8-THF, showing the full spectral window collected.

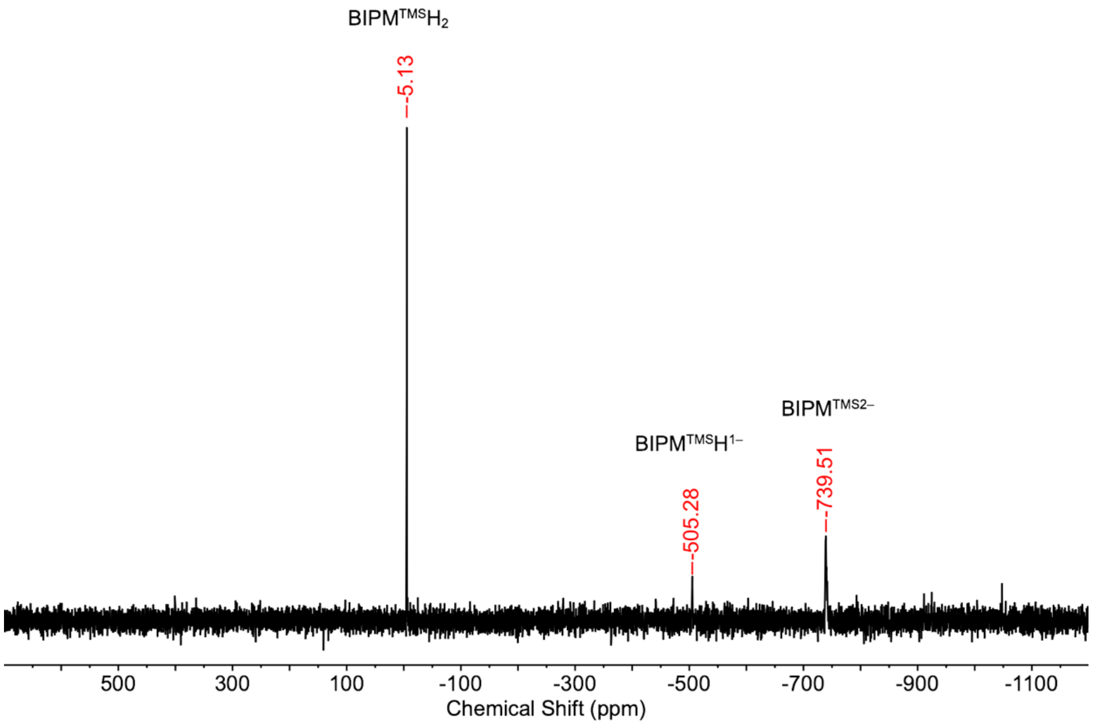

Figure S44. $^{31}P\{^1H\}$ NMR spectrum of **6Np** in d_8-THF.

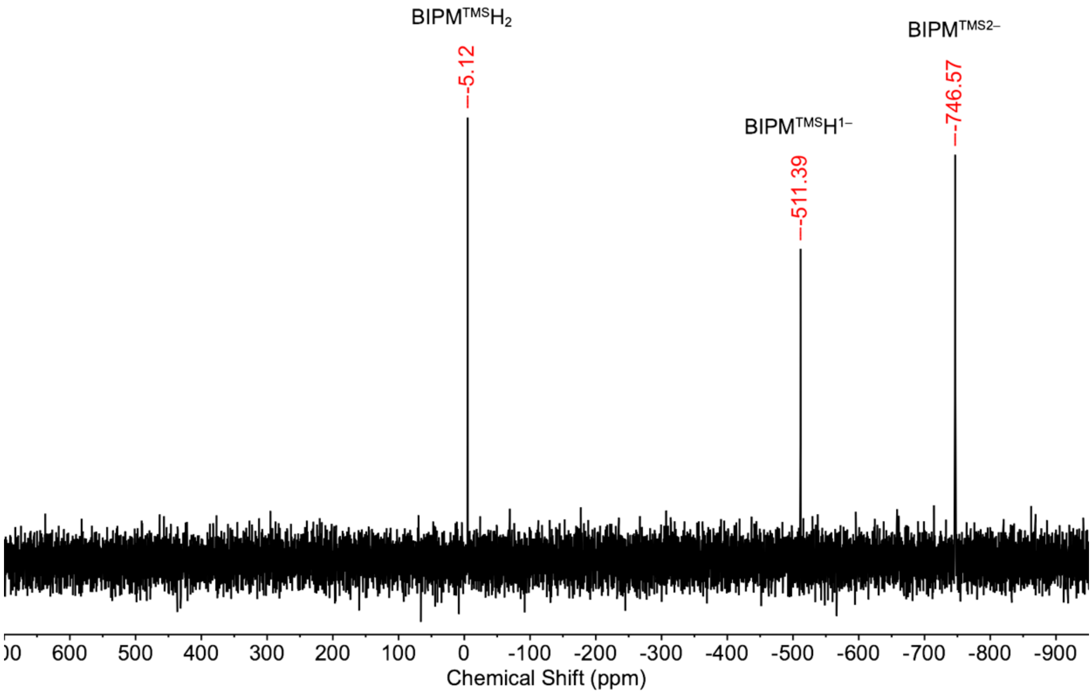

Figure S45. ^{31}P NMR spectrum of **6Np** in d_8-THF.

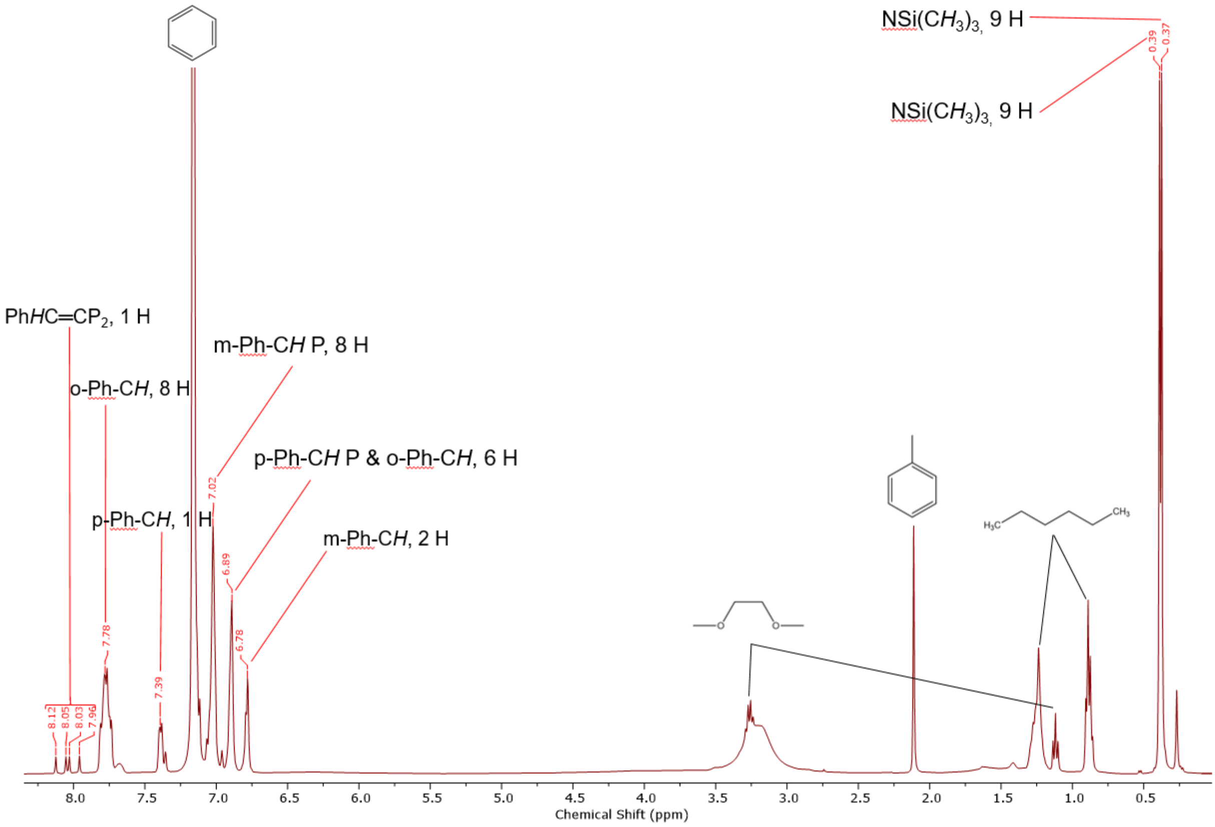

Figure S46. ^{1}H NMR spectrum of *in situ* generated **7** in d_6-benzene.

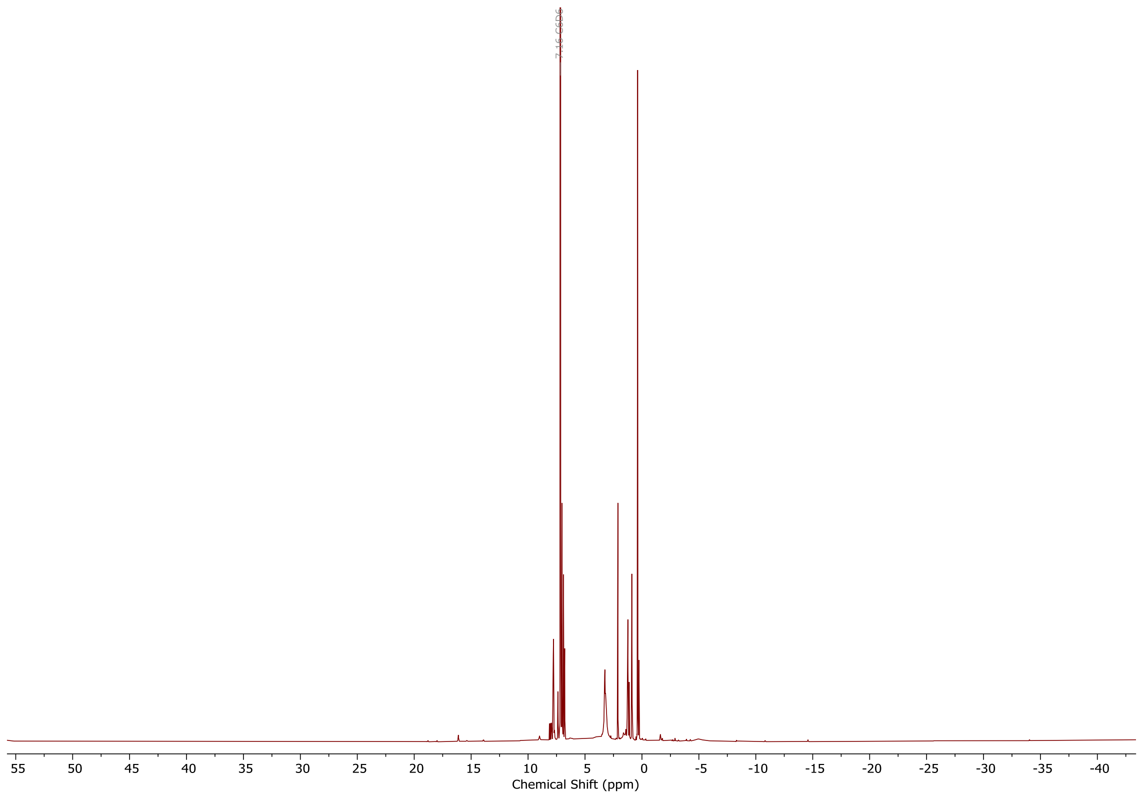

Figure S47. ^{1}H NMR spectrum of *in situ* generated **7** in d_6-benzene, showing the full spectral window collected.

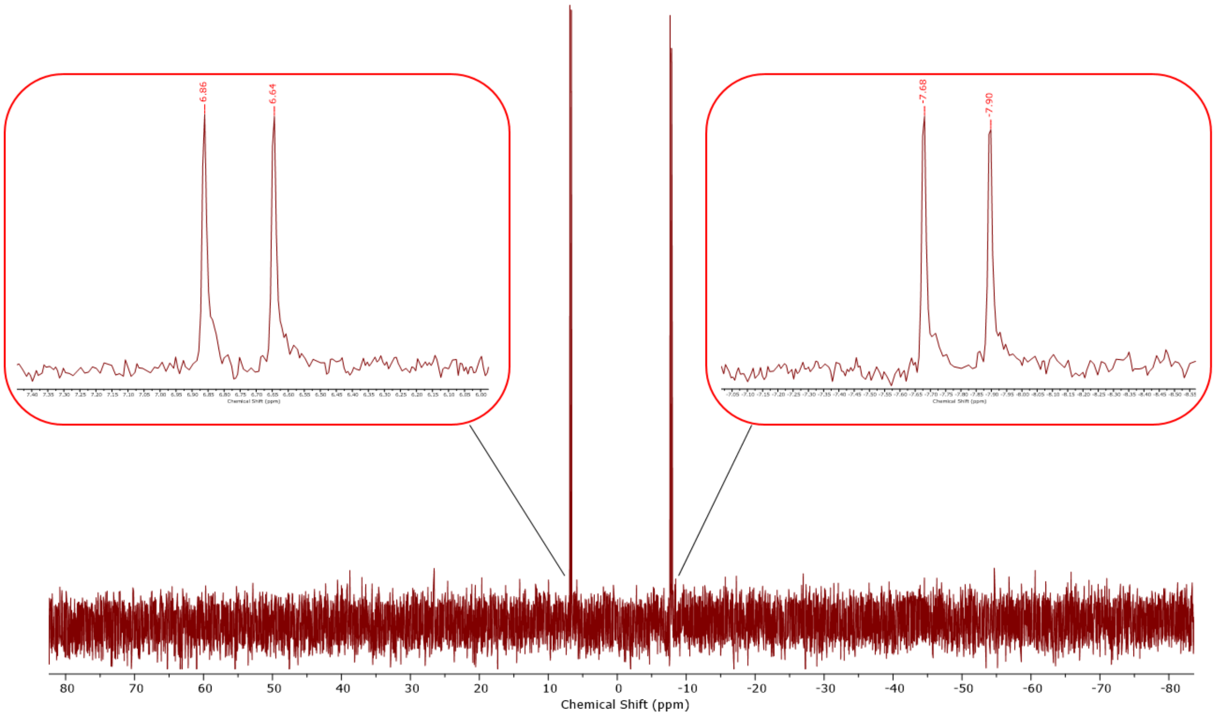

Figure S48. ^{31}P NMR spectrum of *in situ* generated **7** in d_6-benzene.

S7. ATR-IR Spectra

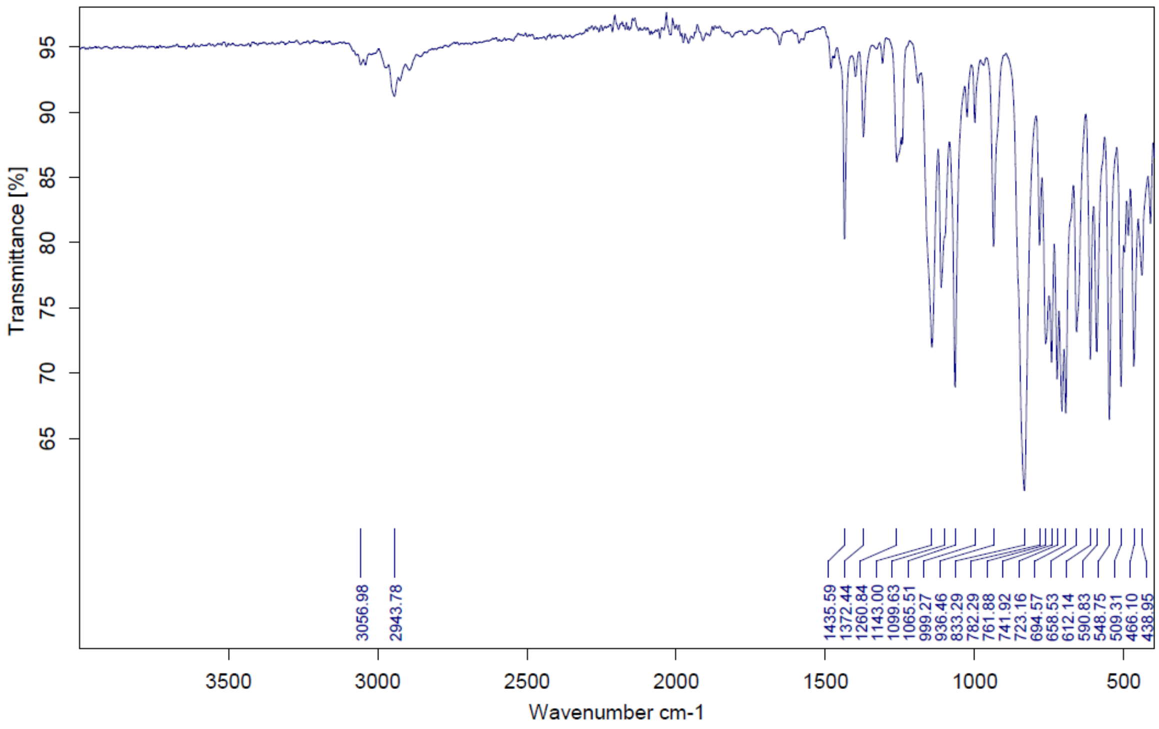

Figure S49. ATR-IR spectrum of **4Ce**.

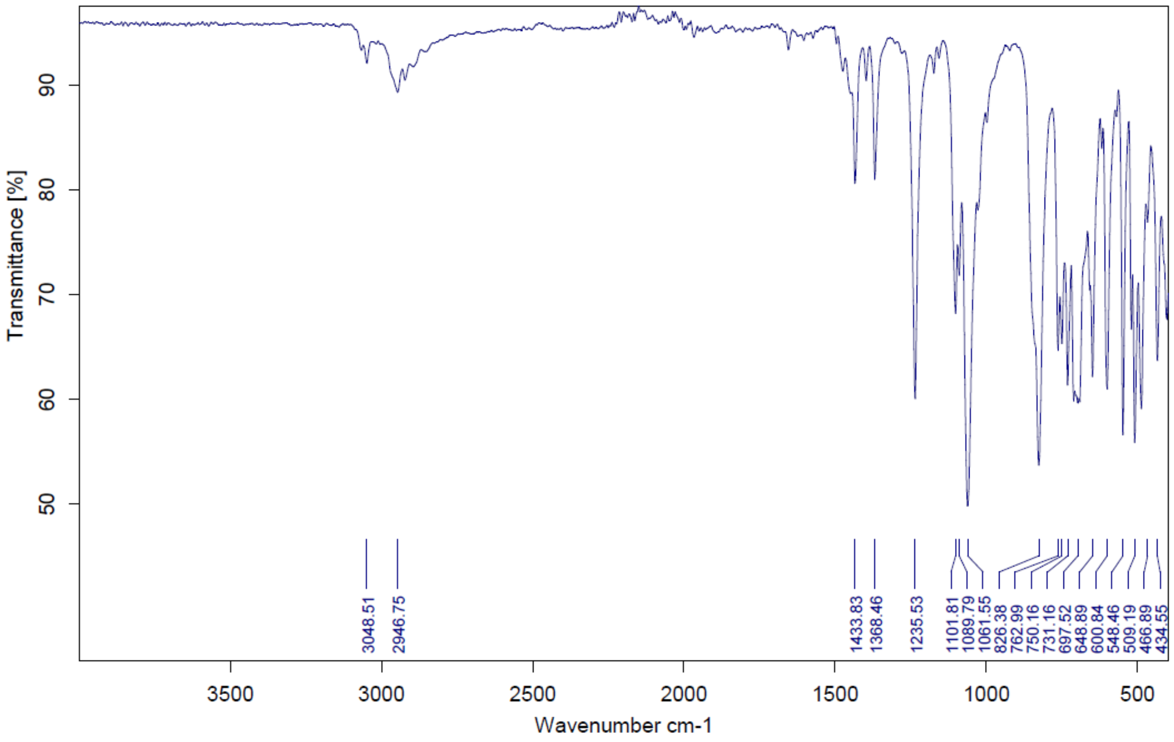

Figure S50. ATR-IR spectrum of **6Ce**.

S8. Magnetic moments determined by NMR spectroscopy (Evans method)

Table S4. Data for the determination of the magnetic moments of complexes **5Np** and **6Np**.

Sample / peak	μ_{eff} / B.M mol^{-1}	[a] sample mass/ g	solvent mass / g	M_r / g mol^{-1}	[b] Δ peak / Hz
**5Np**	1.36	0.0097	0.3704	718.203	98.16
**6Np**	1.46	0.0060	0.4019	908.20	2.84

[a] The small masses engender large errors in this methodology, the results should be cautiously interpreted along with other data. [b] Spectrometer frequency 400.130 MHz. Simple diamagnetic correction of M_r / −2,000,000 applied. ρd_6-benzene = 0.950 g mL^{-1}; ρd_8-THF = 0.985 g mL^{-1}.

We were unable to obtain magnetic susceptibility measurements for complex **4Np** due to the very limited sample quantity combined with difficulty fully solubilizing the sample in d_6-benene at room temperature (for example, it crystallizes overnight from d_6-benzene solutions in NMR tubes). As our sample sealing procedure makes it impractical to safely bring the sample back into the glovebox without decomposition, we were unable to add additional solvent (*e.g.* some d_8-THF) to ensure complete dissolution of the sample, and we did not have enough material to gather new NMR spectra in a different solvent.

S9. Computational studies

Geometry optimizations for **4Np-6Np** were performed using coordinates derived from their crystal structures as the starting points. No constraints were imposed on the structures during the geometry optimizations. The calculations were performed using the Amsterdam Density Functional (ADF) suite version 2017 with standard convergence criteria, Tables S5-S19.[22,23] The DFT geometry optimizations employed Slater type orbital (STO) triple-ζ-plus polarization all-electron basis sets (from the Dirac and ZORA/TZP database of the ADF suite). Scalar relativistic approaches (spin-orbit neglected) were used within the ZORA Hamiltonian[24-26] for the inclusion of relativistic effects and the local density approximation (LDA) with the correlation potential due to Vosko *et al* was used in

all of the calculations.[27] Generalized gradient approximation corrections were performed using the functionals of Becke and Perdew.[28,29] Natural Bond Order (NBO) analyses were carried out with NBO 6.0.19.[30] The Quantum Theory of Atoms in Molecules analysis[31,32] was carried out within the ADF program, and those data were checked by comparing to values computed with Xaim-1.0[33] using WFN files generated by ADF. The ADF-GUI (ADFview) was used to prepare the three-dimensional plots of the electron density. In all cases, Aubau formulations were found with the appropriate spin formulations ($5f^4$ Np(III), quintet; $5f^3$ U(III), quarted, $4f^1$ Ce(III), doublet, $4f^4$ Pm(III), quintet).

Table S5. Computed bond, indices, charges, and spin densities of **4M-6M** (M = Np, U, Ce, Pm).

**Cmpd**	**Bond**	**M-C bond lengths and indices**			**MDC$_q$ charges**		**MDC$_m$ spin density**	
		**Expt.**	**Calc.**	**BI**[a]	**M**	**C**	**M**	**C**
**4Np**	Np-CH$_{BIPM}$	2.753(7)	2.7437	0.59	1.51	−1.64	4.21	−0.01
	Np←C$_{NHC}$	2.676(8)	2.6805	0.83		−0.43		−0.02
**5Np**	Np=C$_{BIPM}$	2.425(7)	2.3899	1.40	1.54	−1.96	4.36	−0.07
**6Np**	Np=C$_{BIPM}$	2.490(6)	2.4700	1.20	1.51	−1.64	4.22	−0.05
	Np←C$_{NHC}$	2.751(6)	2.7321	0.65		−0.44		−0.03
	Np←C$_{NHC}$	2.677(5)	2.6719	0.69		−0.46		−0.03
**4U**	U-CH$_{BIPM}$	-	2.7492	0.58	1.58	−1.65	3.09	−0.01
	U←C$_{NHC}$	-	2.6738	0.82		−0.45		−0.02
**5U**	U=C$_{BIPM}$	-	2.4232	1.28	1.57	−2.00	3.28	−0.04
**6U**	U=C$_{BIPM}$	-	2.4618	1.17	1.62	−1.67	3.08	−0.04
	U←C$_{NHC}$	-	2.7153	0.77		−0.50		−0.03
	U←C$_{NHC}$	-	2.6538	0.81		−0.48		−0.03
**4Ce**	Ce-CH$_{BIPM}$	2.768(6)	2.7717	0.46	1.20	−1.54	1.04	−0.01
	Ce←C$_{NHC}$	2.731(8)	2.7492	0.60		−0.31		−0.01
**5Ce**	Ce=C$_{BIPM}$	2.4772(19)	2.4402	1.05	1.32	−1.82	1.07	−0.01
**6Ce**	Ce=C$_{BIPM}$	2.519(2)	2.4880	0.96	1.29	−1.53	1.01	−0.01
	Ce←C$_{NHC}$	2.737(3)	2.7576	0.52		−0.38		−0.01
	Ce←C$_{NHC}$	2.806(2)	2.8207	0.58		−0.36		−0.01
**4Pm**	Pm-CH$_{BIPM}$	-	2.7674	0.31	1.06	−1.47	4.38	−0.04
	Pm←C$_{NHC}$	-	2.7061	0.39		−0.26		−0.05
**5Pm**	Pm=C$_{BIPM}$	-	2.4077	0.94	1.26	−1.76	4.40	−0.02
**6Pm**	Pm=C$_{BIPM}$	-	2.4809	0.76	1.11	−1.48	4.39	−0.02
	Pm←C$_{NHC}$	-	2.8097	0.28		−0.31		−0.02
	Pm←C$_{NHC}$	-	2.7385	0.33		−0.29		−0.02

[a] Nalewajski-Mrozek bond indices.

Table S6. Computed NBO data for **4M-6M** (M = Np, U, Ce, Pm).

Cmpd	Bond	M-C σ-bond (%)				M-C π-bond (%)			
		M[a]	C	M s/p/d/f	C s/p	M	C	M s/p/d/f	C s/p
**4Np**	Np-CH_{BIPM}	9	91	9/0/45/46	9/91				
	Np←C_{NHC}	0	100		45/55				
**5Np**	Np=C_{BIPM}	17	83	4/1/32/63	13/87	14	86	0/0/38/62	0/100
**6Np**	Np=C_{BIPM}	15	85	9/1/39/51	22/78	10	90	0/1/43/56	1/99
	Np←C_{NHC}	0	100		46/54				
	Np←C_{NHC}	0	100		46/54				
**4U**	U-CH_{BIPM}	9	91	8/0/47/45	8/92				
	U←C_{NHC}	0	100		44/56				
**5U**	U=C_{BIPM}	14	86	4/1/42/53	14/86	13	87	0/0/40/60	0/100
**6U**	U=C_{BIPM}	14	86	10/1/46/43	24/76	10	90	0/1/50/49	1/99
	U←C_{NHC}	0	100		46/54				
	U←C_{NHC}	0	100		46/54				
**4Ce**	Ce-CH_{BIPM}	0	100		6/94				
	Ce←C_{NHC}	0	100		45/55				
**5Ce**	Ce=C_{BIPM}	10	90	1/1/61/37	8/92	8	92	0/0/65/35	0/100
**6Ce**	Ce=C_{BIPM}	9	91	7/1/65/27	20/80	7	93	2/1/60/37	2/98
	Ce←C_{NHC}	0	100		46/54				
	Ce←C_{NHC}	0	100		46/54				
**4Pm**	Pm-CH_{BIPM}	10	90	5/0/32/63	6/97				
	Pm←C_{NHC}	0	100		45/55				
**5Pm**	Pm=C_{BIPM}	18	82	1/0/24/75	7/93	19	81	0/0/20/80	0/100
**6Pm**	Pm=C_{BIPM}	15	85	5/1/31/63	17/83	14	86	1/0/24/75	2/98
	Pm←C_{NHC}	0	100		44/56				
	Pm←C_{NHC}	0	100		43/57				

[a] The NBO cut-off is 5%, so M% = 0 means only that the M contribution to that bond is <5%.

Table S7. Computed QTAIM data for **4M-6M** (M = Np, U, Ce, Pm).

Cmpd	Bond	ρ^a	$\nabla^2\rho^b$	H^c	ε^d
**4Np**	Np-CH_{BIPM}	0.04	0.09	-0.07	0.05
	Np←C_{NHC}	0.05	0.11	-0.01	0.01
**5Np**	Np=C_{BIPM}	0.08	0.13	-0.04	0.21
**6Np**	Np=C_{BIPM}	0.08	0.12	-0.03	0.18
	Np←C_{NHC}	0.04	0.11	-0.01	0.03
	Np←C_{NHC}	0.05	0.11	-0.01	0.03
**4U**	U-CH_{BIPM}	0.04	0.09	-0.01	0.05
	U←C_{NHC}	0.05	0.01	-0.01	0.01
**5U**	U=C_{BIPM}	0.08	0.12	-0.03	0.20
**6U**	U=C_{BIPM}	0.08	0.11	-0.03	0.17
	U←C_{NHC}	0.05	0.10	-0.01	0.03
	U←C_{NHC}	0.05	0.11	-0.01	0.03
**4Ce**	Ce-CH_{BIPM}	0.04	0.08	-0.01	0.05
	Ce←C_{NHC}	0.04	0.08	-0.01	0.01
**5Ce**	Ce=C_{BIPM}	0.07	0.12	-0.03	0.22
**6Ce**	Ce=C_{BIPM}	0.07	0.10	-0.02	0.19
	Ce←C_{NHC}	0.04	0.08	-0.01	0.03
	Ce←C_{NHC}	0.04	0.08	-0.01	0.03
**4Pm**	Pm-CH_{BIPM}	0.04	0.08	-0.01	0.05
	Pm←C_{NHC}	0.04	0.08	-0.01	0.05
**5Pm**	Pm=C_{BIPM}	0.07	0.12	-0.03	0.16
**6Pm**	Pm=C_{BIPM}	0.06	0.11	-0.02	0.13
	Pm←C_{NHC}	0.03	0.07	-0.01	0.05
	Pm←C_{NHC}	0.04	0.08	-0.01	0.02

[a] Topological electron density. [b] Laplacian. [c] Electronic energy density. [d] Bond ellipticity.

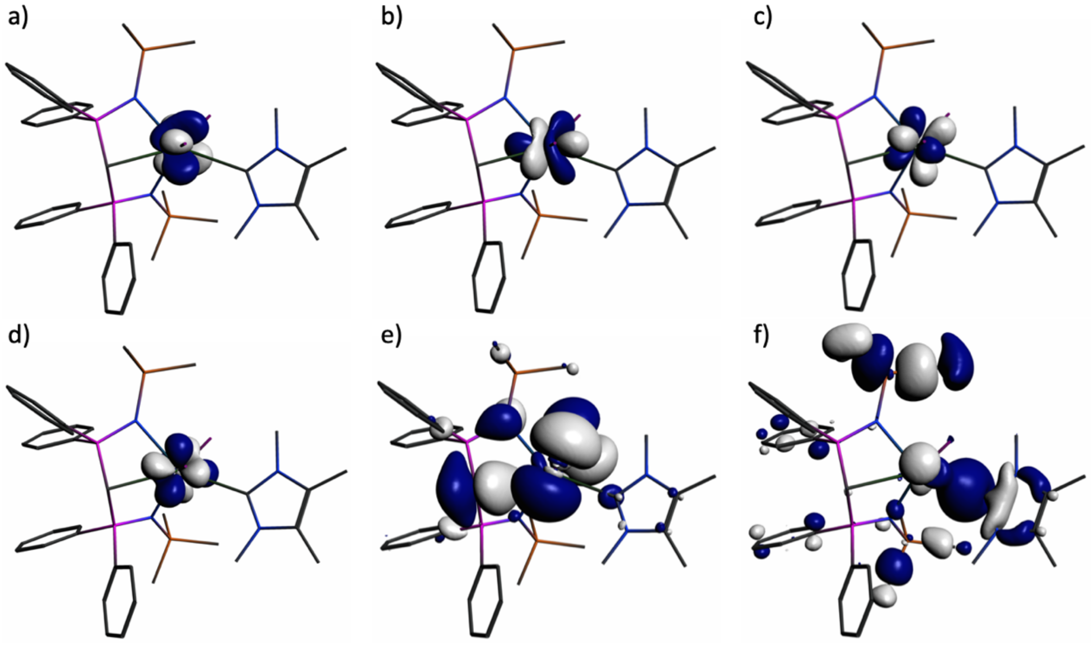

Figure S51. Selected frontier Kohn Sham molecular orbitals of **4Np**. a) HOMO (284a, −2.792 eV), b) HOMO−1 (283a, −2.802 eV), c) HOMO−2 (282a, −2.848 eV), d) HOMO−3 (281a, −2.864 eV), e) HOMO−5 (279a, −5.060 eV), f) HOMO−23 (261a, −6.663 eV). Hydrogen atoms are omitted for clarity.

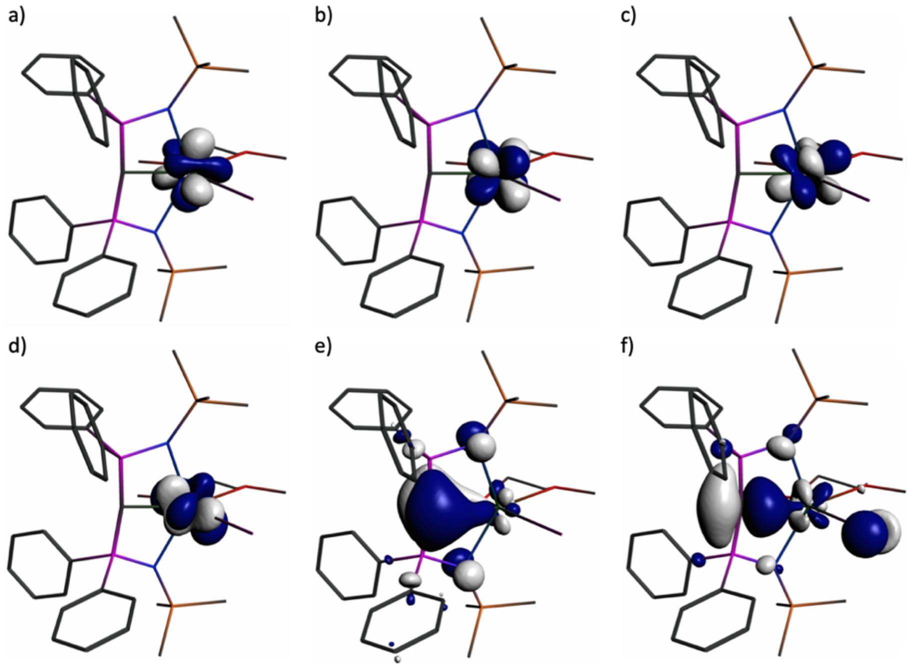

Figure S52. Selected frontier Kohn Sham molecular orbitals of **5Np**. a) HOMO (248a, −2.363 eV), b) HOMO−1 (247a, −2.503 eV), c) HOMO−2 (246a, −2.533 eV), d) HOMO−3 (245a, −2.565 eV), e) HOMO−4 (244a, −4.515 eV), f) HOMO−5 (243a, −4.951 eV). Hydrogen atoms are omitted for clarity.

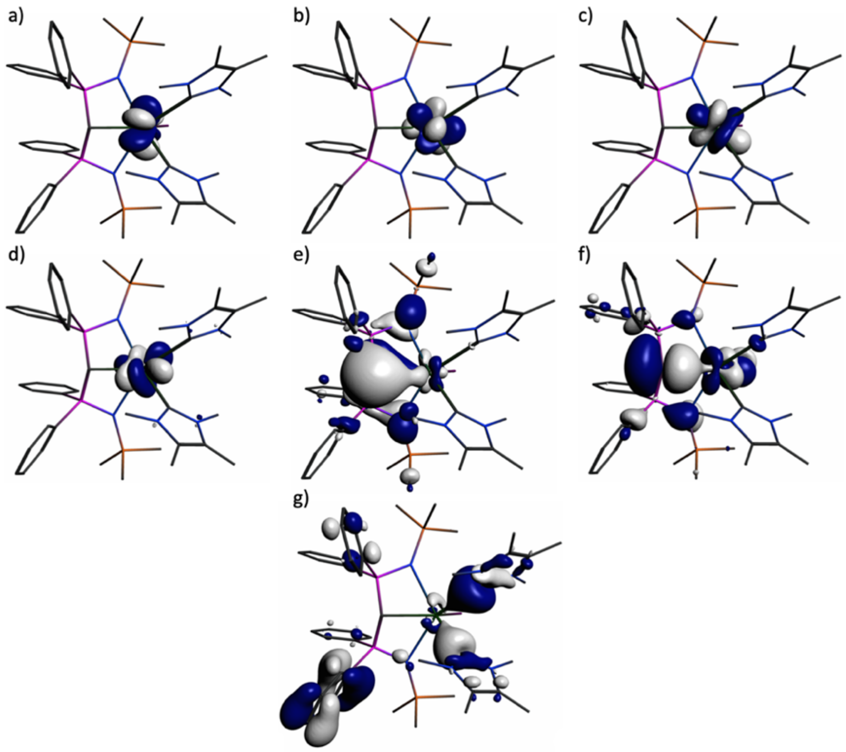

Figure S53. Selected frontier Kohn Sham molecular orbitals of **6Np**. a) HOMO (291a, –2.195 eV), b) HOMO−1 (290a, −2.243 eV), c) HOMO−2 (289a, −2.319 eV), d) HOMO−3 (288a, −2.392 eV), e) HOMO−4 (287a, –4.297 eV), f) HOMO−5 (286a, –4.521 eV), g) HOMO−19 (272a, –6.141 eV). Hydrogen atoms are omitted for clarity.

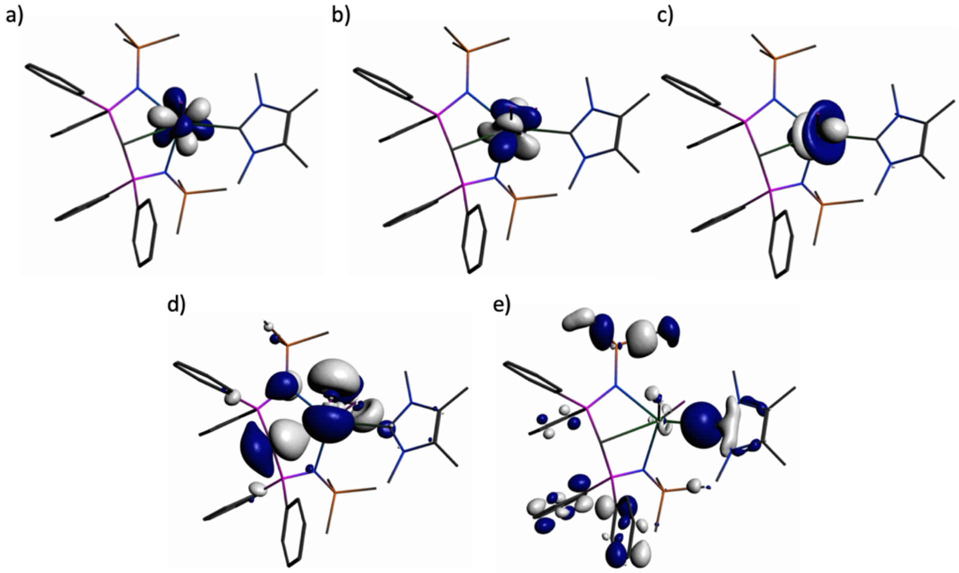

Figure S54. Selected frontier Kohn Sham molecular orbitals of **4U**. a) HOMO (283a, –2.251 eV), b) HOMO–1 (282a, –2.268 eV), c) HOMO–2 (281a, –2.299 eV), d) HOMO–4 (279, –5.121 eV), e) HOMO–22 (261a, –6.424 eV). Hydrogen atoms are omitted for clarity.

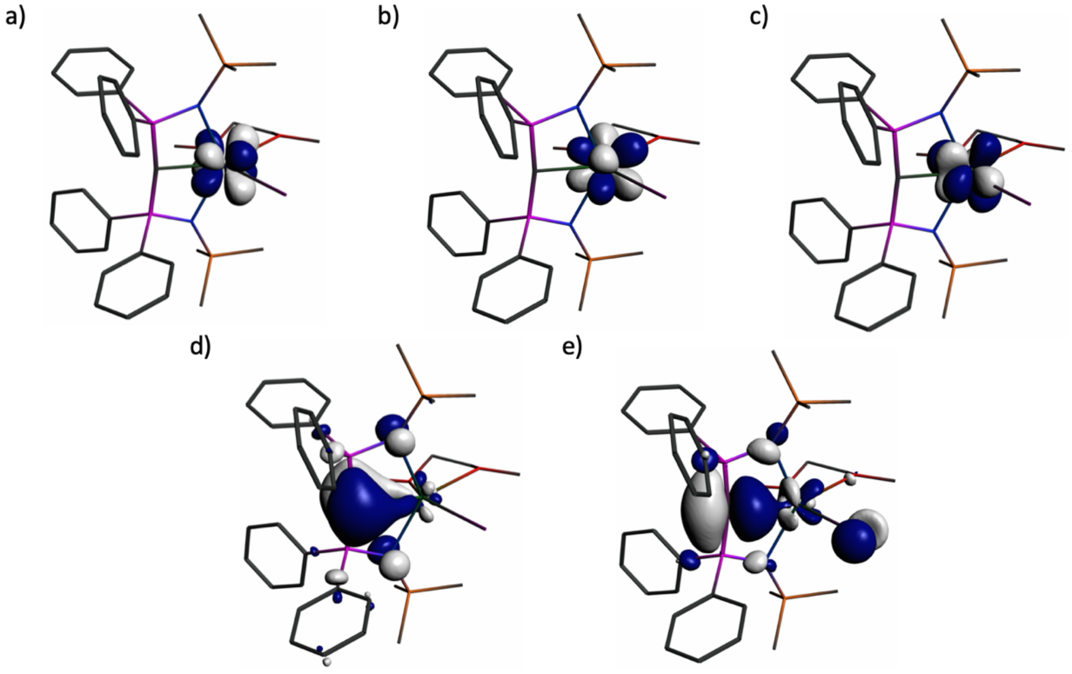

Figure S55. Selected frontier Kohn Sham molecular orbitals of **5U**. a) HOMO (247a, −1.941 eV), b) HOMO−1 (246a, −1.969 eV), c) HOMO−2 (245a, −1.999 eV), d) HOMO−3 (244a, −4.538 eV), e) HOMO−4 (243a, −4.983 eV). Hydrogen atoms are omitted for clarity.

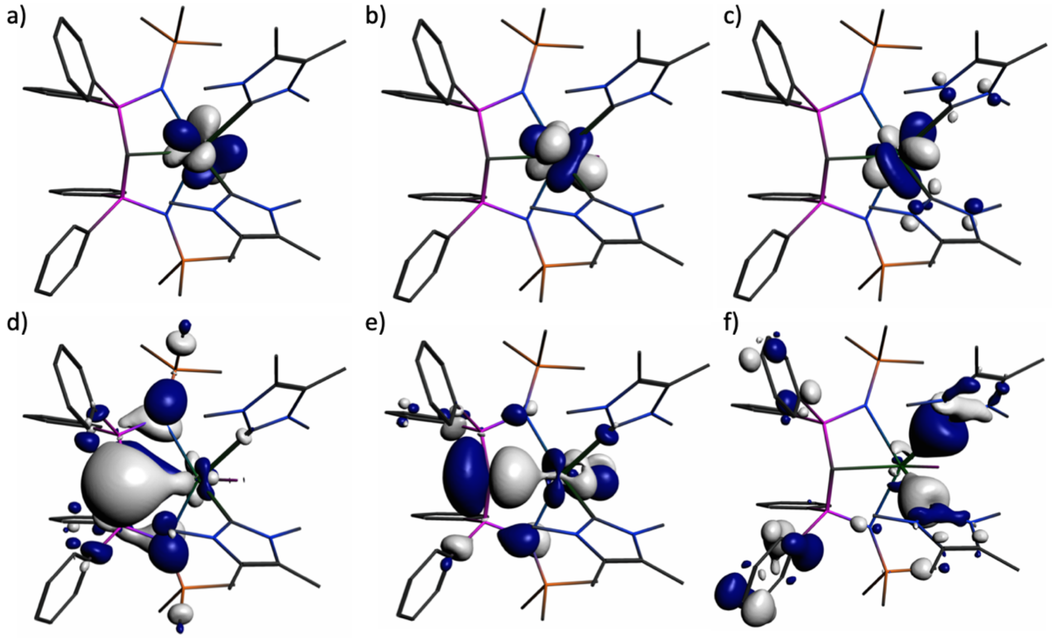

Figure S56. Selected frontier Kohn Sham molecular orbitals of **6U**. a) HOMO (290a, −1.844 eV), b) HOMO−1 (289a, −1.870 eV), c) HOMO−2 (288a, −1.989 eV), d) HOMO−3 (287a, −24.364 eV), e) HOMO−4 (286a, −4.627 eV), f) HOMO−18 (272a, −6.160 eV). Hydrogen atoms are omitted for clarity.

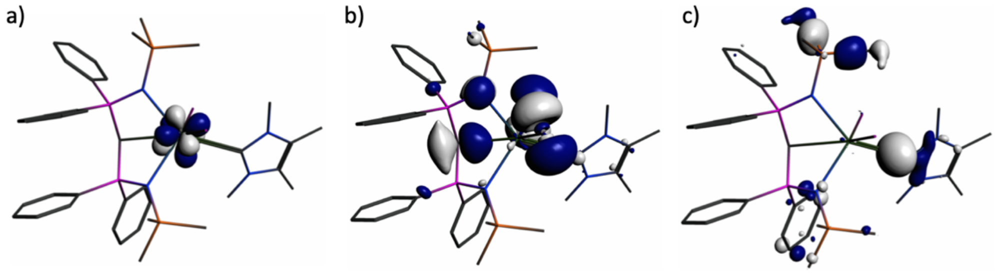

Figure S57. Selected frontier Kohn Sham molecular orbitals of **4Ce**. a) HOMO (265a, −2.461 eV), b) HOMO−2 (263a, −5.032 eV), c) HOMO−19 (246a, −6.549 eV). Hydrogen atoms are omitted for clarity.

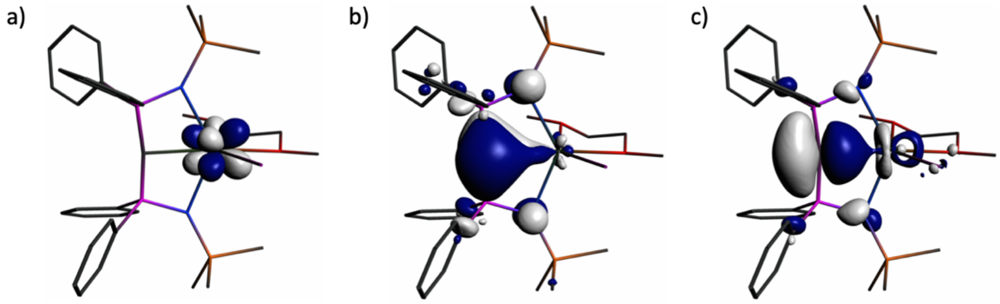

Figure S58. Selected frontier Kohn Sham molecular orbitals of **5Ce**. a) HOMO (229a, −2.086 eV), b) HOMO−1 (228a, −4.406 eV), c) HOMO−2 (227a, −4.770 eV). Hydrogen atoms are omitted for clarity.

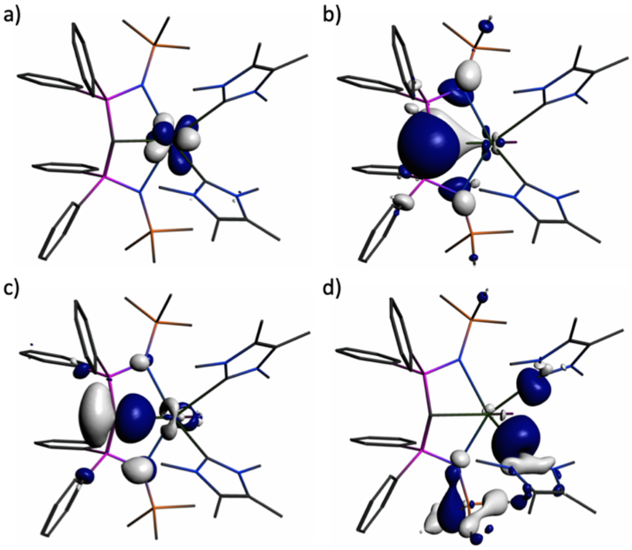

Figure S59. Selected frontier Kohn Sham molecular orbitals of **6Ce**. a) HOMO (272a, −1.952 eV), b) HOMO−1 (271a, −4.240 eV), c) HOMO−2 (270a, −4.391 eV), d) HOMO−21 (251a, −6.457 eV). Hydrogen atoms are omitted for clarity.

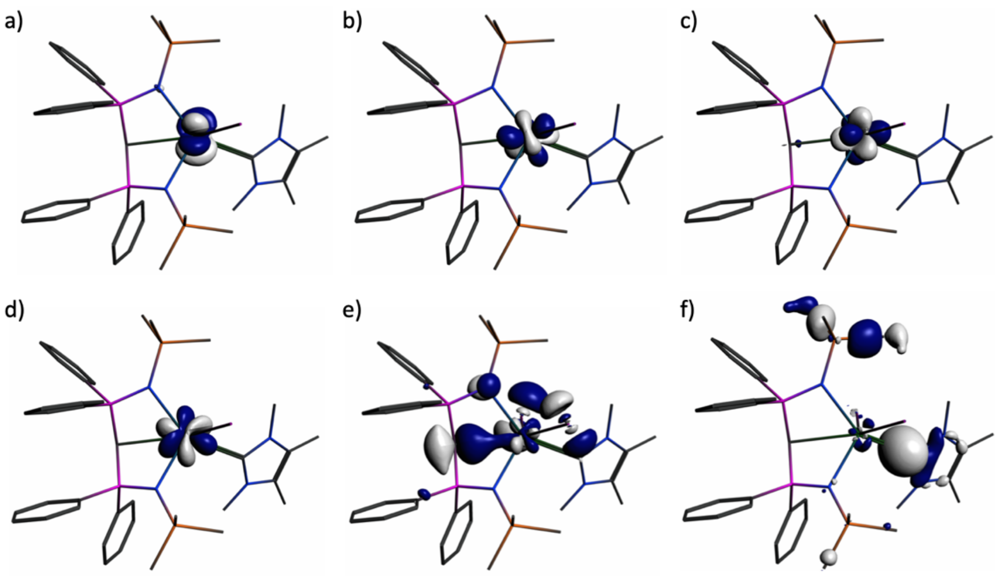

Figure S60. Selected frontier Kohn Sham molecular orbitals of **4Pm**. a) HOMO (268a, −4.231 eV), b) HOMO−1 (267a, −4.254 eV), c) HOMO−2 (266a, −4.294 eV), d) HOMO−3 (265a, −4.329 eV), e) HOMO−5 (263a, −5.042 eV), f) HOMO−22 (246a, −6.583 eV). Hydrogen atoms are omitted for clarity.

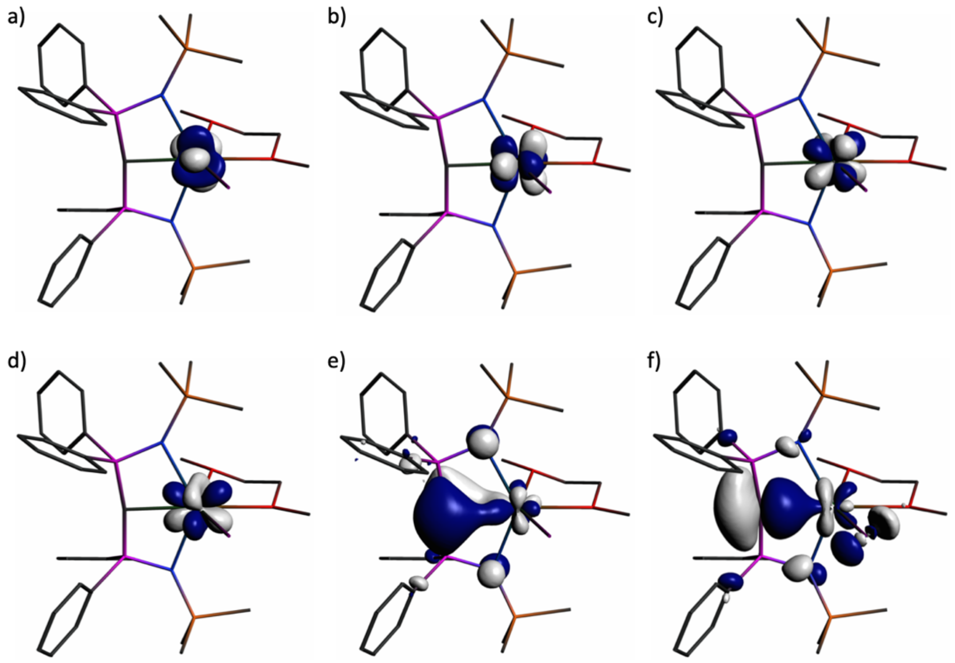

Figure S61. Selected frontier Kohn Sham molecular orbitals of **5Pm**. a) HOMO (232a, −3.673 eV), b) HOMO−1 (231a, −3.776 eV), c) HOMO−2 (230a, −3.790 eV), d) HOMO−3 (229a, −3.822 eV), e) HOMO−4 (228a, −4.469 eV), f) HOMO−5 (227a, −4.872 eV). Hydrogen atoms are omitted for clarity.

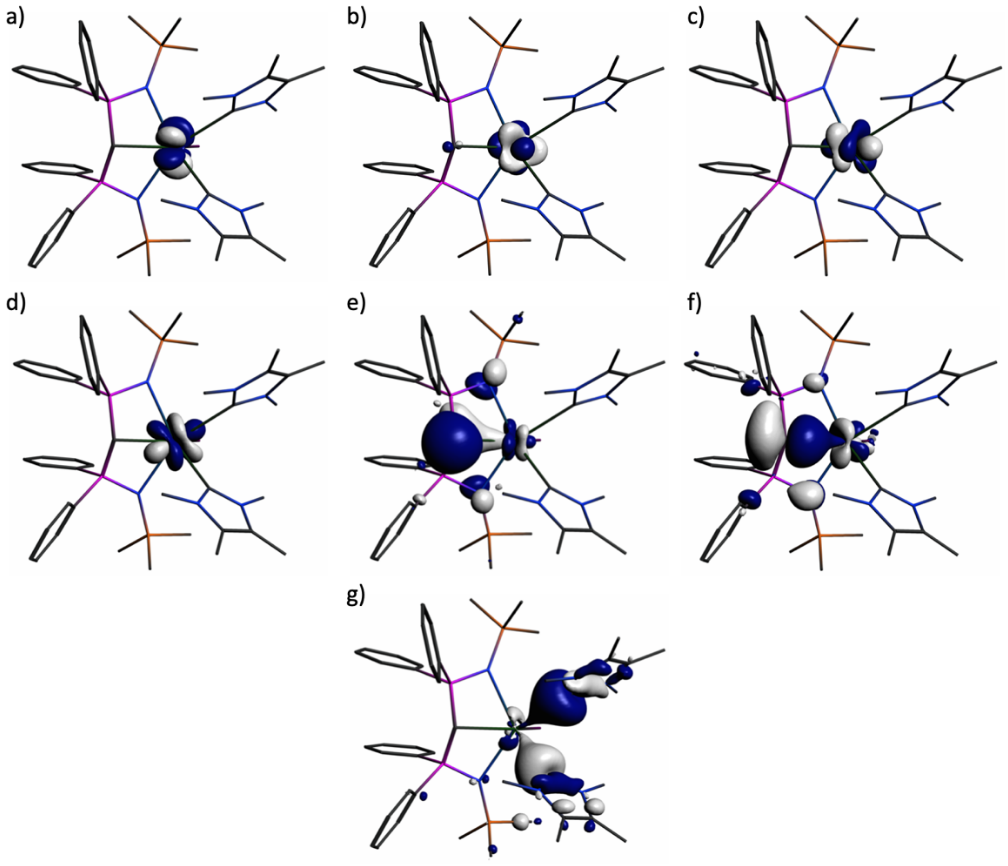

Figure S62. Selected frontier Kohn Sham molecular orbitals of **6Pm**. a) HOMO (275a, –3.477 eV), b) HOMO–1 (274a, –3.505 eV), c) HOMO–2 (273a, –3.551 eV), d) HOMO–3 (272a, –3.600 eV), e) HOMO–4 (271a, –4.259 eV), f) HOMO–5 (270a, –4.444 eV), g) HOMO–18 (257a, –5.995 eV). Hydrogen atoms are omitted for clarity.

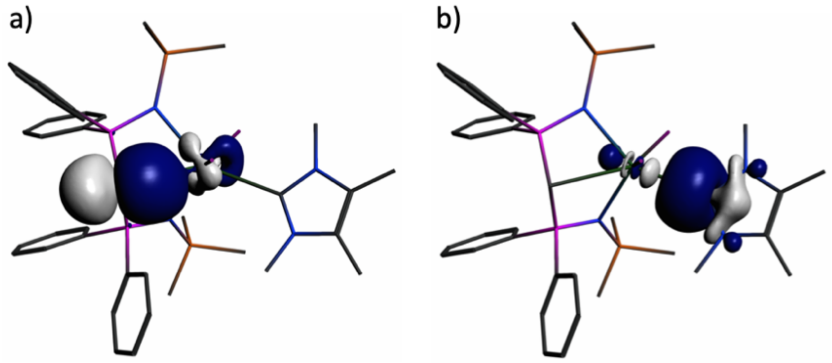

Figure S63. Selected NBOs of **4Np**. a) Np-C_{BIPM} σ-bond, b) Np-C_{NHC} σ-bond.

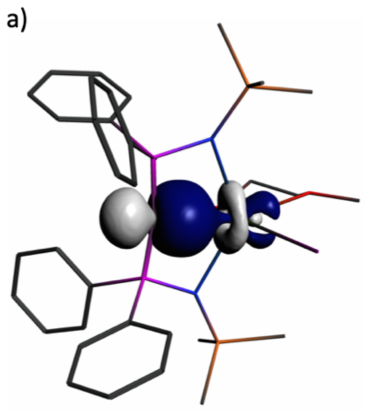

Figure S64. Selected NBOs of **5Np**. a) Np-C_{BIPM} σ-bond, b) Np-C_{BIPM} π-bond.

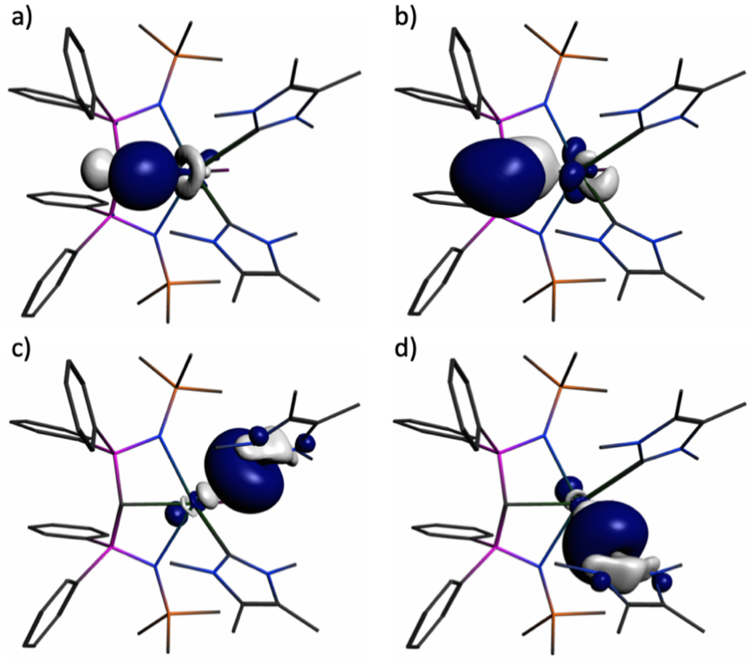

Figure S65. Selected NBOs of **6Np**. a) Np-C_{BIPM} σ-bond, b) Np-C_{BIPM} π-bond, c) Np-C_{NHC} σ-bond, d) Np-C_{NHC} σ-bond.

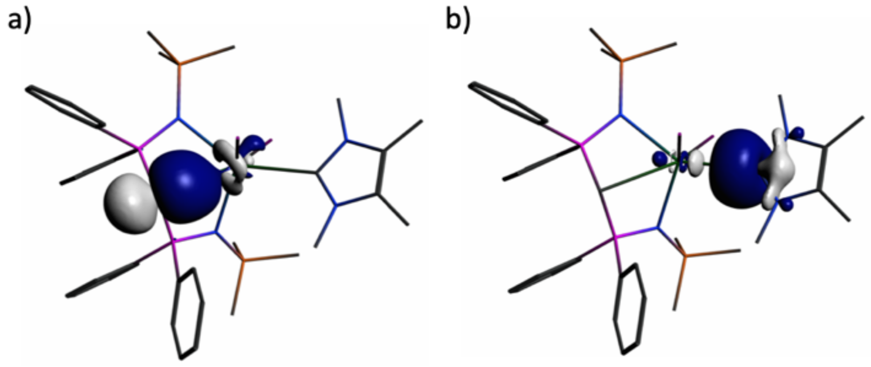

Figure S66. Selected NBOs of **4U**. a) U-C_{BIPM} σ-bond, b) U-C_{NHC} σ-bond.

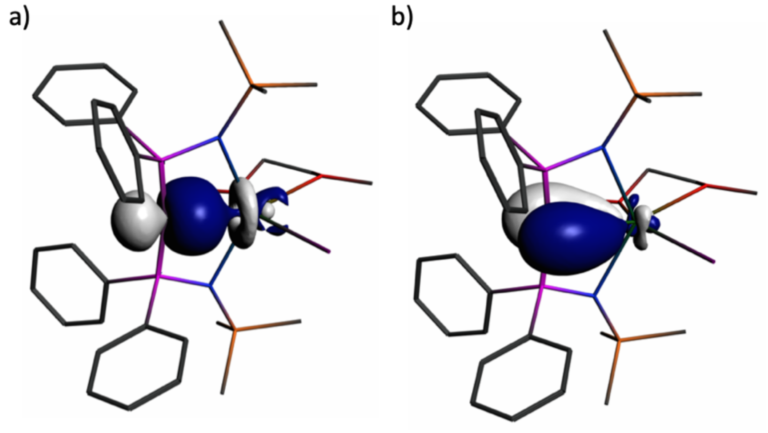

Figure S67. Selected NBOs of **5U**. a) U-C_{BIPM} σ-bond, b) U-C_{BIPM} π-bond.

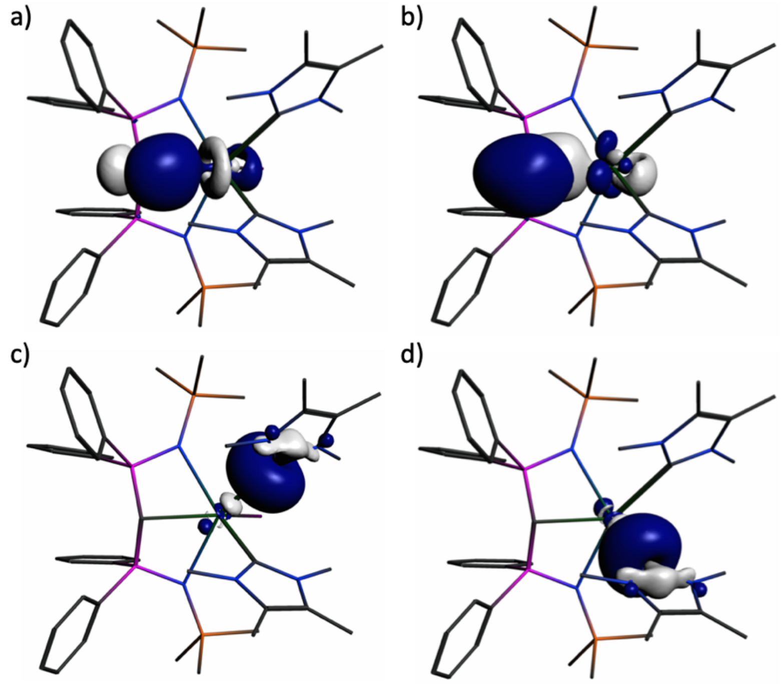

Figure S68. Selected NBOs of **6U**. a) U-C_{BIPM} σ-bond, b) U-C_{BIPM} π-bond, c) U-C_{NHC} σ-bond, d) U-C_{NHC} σ-bond.

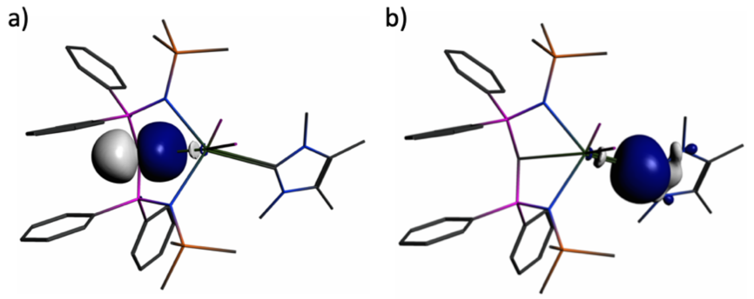

Figure S69. Selected NBOs of **4Ce**. a) Ce-C_{BIPM} σ-bond, b) Ce-C_{NHC} σ-bond.

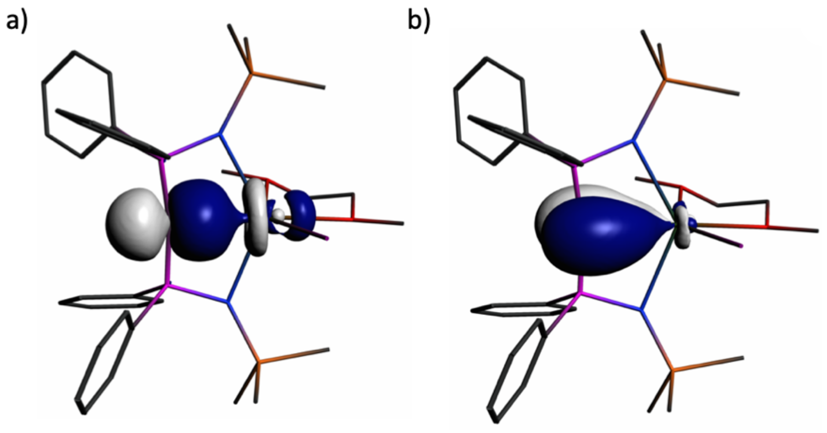

Figure S70. Selected NBOs of **5Ce**. a) Ce-C_{BIPM} σ-bond, b) Ce-C_{BIPM} π-bond.

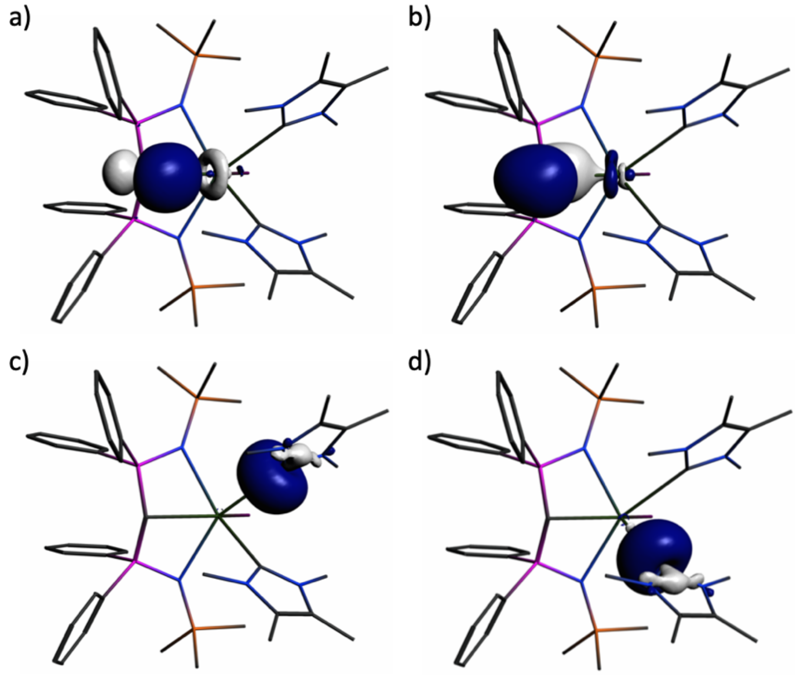

Figure S71. Selected NBOs of **6Ce**. a) Ce-C_{BIPM} σ-bond, b) Ce-C_{BIPM} π-bond, c) Ce-C_{NHC} σ-bond, d) Ce-C_{NHC} σ-bond.

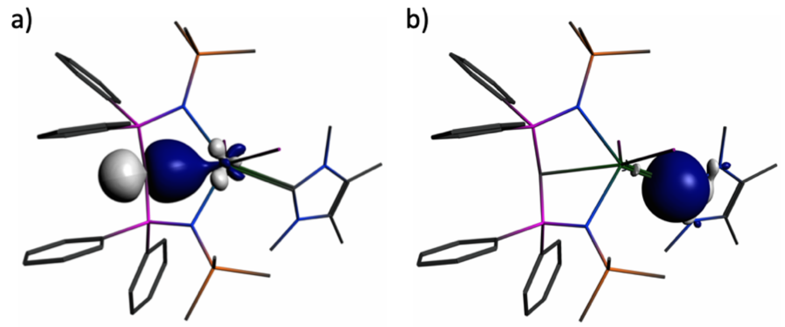

Figure S72. Selected NBOs of **4Pm**. a) Pm-C_{BIPM} σ-bond, b) Pm-C_{NHC} σ-bond.

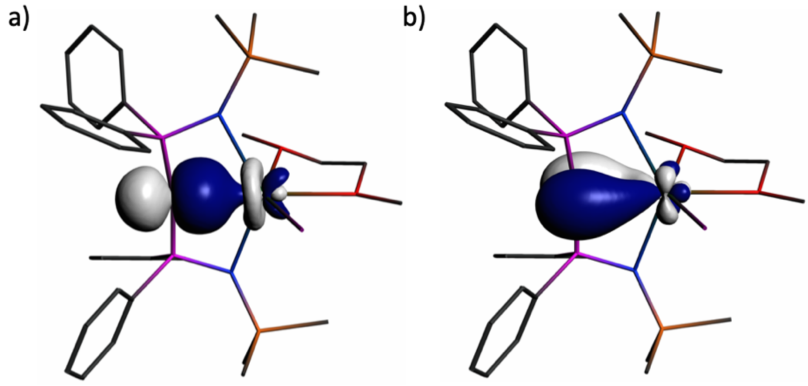

Figure S73. Selected NBOs of **5Pm**. a) Pm-C_{BIPM} σ-bond, b) Pm-C_{BIPM} π-bond.

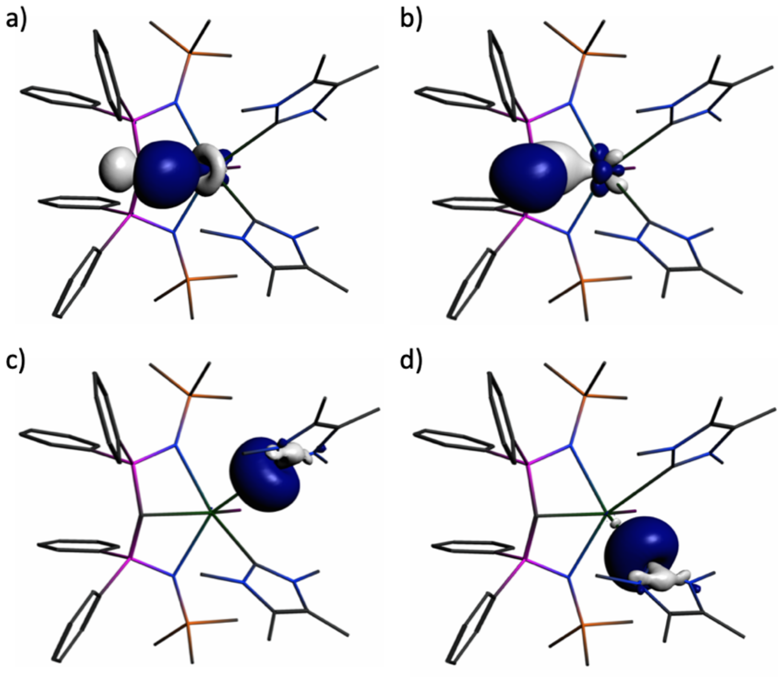

Figure S74. Selected NBOs of **6Pm**. a) Pm-C_{BIPM} σ-bond, b) Pm-C_{BIPM} π-bond, c) Pm-C_{NHC} σ-bond, d) Pm-C_{NHC} σ-bond.

Table S8. Final coordinates and energy for a single point energy calculation of geometry optimized **4Np**.

1.C	3.631314	-0.615213	-3.403553
2.C	3.111201	2.397281	-3.575070
3.C	2.162714	-4.116339	-3.106081
4.C	-3.667199	0.232367	-2.763600
5.C	2.038314	-7.083148	-2.472335
6.C	-0.601119	4.191248	-2.631498
7.C	0.071648	3.086050	-2.101225
8.C	1.201468	-6.045214	-1.800176
9.C	-3.201120	-2.679628	-1.911860
10.C	-0.872326	5.300246	-1.824976

11.C	0.408223	-3.932624	-1.355642
12.C	4.926269	1.194232	-1.392243
13.C	0.290173	-6.156532	-0.780960
14.C	0.470999	3.078183	-0.755071
15.C	-0.494670	5.291248	-0.476346
16.C	-0.184632	-7.352221	-0.024017
17.C	-4.731629	-0.813203	-0.088794
18.C	-3.119016	2.422805	0.026706
19.C	-3.828150	3.614763	0.207732
20.C	0.160788	4.181978	0.061404
21.C	-1.092476	-4.525603	0.537613
22.C	0.344937	0.557058	0.894479
23.C	-2.371386	1.872461	1.075413
24.C	3.160907	3.534942	0.950868
25.C	2.679346	2.216096	0.996883
26.C	-3.786707	4.275121	1.438075
27.C	3.282647	1.296653	1.874821
28.C	4.199119	3.936754	1.797285
29.C	-2.314108	2.556027	2.305333
30.C	-3.020327	3.746516	2.485154
31.C	-1.767711	-0.638848	2.369959
32.C	4.321499	1.701371	2.714243
33.C	4.776331	3.024644	2.685649
34.C	-0.843713	-1.585593	2.848074
35.C	-3.012557	-0.521192	3.012451
36.C	-1.155192	-2.383449	3.952460
37.C	-3.323664	-1.324796	4.113219
38.C	-2.395107	-2.256240	4.588465
39.H	4.526569	-0.451169	-4.025997
40.H	2.276590	2.222665	-4.270050
41.H	4.026461	2.523331	-4.176521
42.H	2.794075	-0.857549	-4.072459
43.H	2.401936	-4.853270	-3.880043
44.H	1.817211	-7.160301	-3.548063
45.H	-4.197984	-0.346748	-3.535582
46.H	1.690022	-3.250613	-3.587040
47.H	-0.904215	4.184883	-3.679347
48.H	-2.753476	0.626696	-3.229722
49.H	3.842641	-1.481254	-2.756707
50.H	2.924040	3.346491	-3.050353
51.H	-4.318989	1.073480	-2.483010
52.H	3.112962	-6.870397	-2.365975
53.H	0.291577	2.223457	-2.729709
54.H	3.089835	-3.805716	-2.603124
55.H	-2.334107	-2.877568	-2.559715
56.H	-4.108163	-2.874668	-2.507240
57.H	1.850282	-8.066709	-2.026133
58.H	-1.382817	6.169976	-2.242900
59.H	5.776015	1.197889	-2.095187
60.H	-3.193751	-3.399853	-1.081319
61.H	-3.140123	1.924879	-0.939655
62.H	4.957254	2.134964	-0.823353

63.H	-4.409490	4.026416	-0.618262
64.H	-5.642516	-1.117597	-0.631596
65.H	0.355923	-8.247262	-0.353973
66.H	5.078042	0.363239	-0.686603
67.H	-1.259118	-7.538609	-0.178763
68.H	-0.714301	6.149190	0.160389
69.H	-4.907157	0.199485	0.304130
70.H	-1.377149	-3.474002	0.442641
71.H	-1.998783	-5.142331	0.486489
72.H	2.729770	4.253657	0.254763
73.H	-4.608873	-1.497687	0.763953
74.H	-0.017092	-7.244527	1.058611
75.H	0.438727	4.179268	1.115621
76.H	-4.342459	5.203494	1.580963
77.H	-0.605992	-4.671556	1.511037
78.H	2.969207	0.250329	1.877635
79.H	0.773167	0.413736	1.888243
80.H	4.557295	4.966385	1.757714
81.H	0.125466	-1.711805	2.358459
82.H	-3.742989	0.205847	2.658222
83.H	-1.720893	2.152366	3.127620
84.H	-2.975658	4.261639	3.445870
85.H	4.781142	0.975227	3.386144
86.H	5.584839	3.340501	3.346985
87.H	-0.421044	-3.103994	4.315961
88.H	-4.292978	-1.216011	4.601611
89.H	-2.635818	-2.878716	5.452142
90.I	-0.190172	-0.996210	-4.175239
91.I	2.691841	-2.532431	0.816460
92.N	1.234846	-4.690505	-2.141137
93.N	1.908097	0.706462	-1.353878
94.N	-0.181553	-4.861526	-0.543743
95.N	-1.718709	-0.460507	-0.535008
96.Np	0.536569	-1.263146	-1.149496
97.P	1.353593	1.606171	-0.114823
98.P	-1.389040	0.331712	0.849304
99.Si	3.339953	0.946162	-2.384888
100.Si	-3.269856	-0.897820	-1.291322

Energy: −590.12253057 eV

Table S9. Final coordinates and energy for a single point energy calculation of geometry optimized **5Np**.

1.C	-2.809863	-3.312272	-3.766319
2.C	-2.515567	-1.947020	-3.683400
3.C	1.800292	-3.167378	-3.538957
4.C	-2.310310	2.985309	-3.263628
5.C	-2.281358	-4.197957	-2.819511
6.C	-3.649243	3.266426	-2.957841
7.C	-1.699920	-1.466971	-2.655470
8.C	-1.460180	2.470212	-2.284409

9.C	4.226375	-2.441306	-1.855803
10.C	-1.460028	-3.719389	-1.795167
11.C	-4.130923	3.020391	-1.669508
12.C	-1.162809	-2.348048	-1.704838
13.C	5.651709	0.985491	-1.023489
14.C	2.392232	-4.698633	-0.927746
15.C	-1.935100	2.225862	-0.983132
16.C	-3.281237	2.498751	-0.687393
17.C	2.817819	4.227318	-0.365291
18.C	-0.047036	5.322945	-0.162568
19.C	-0.234919	-0.034298	-0.103806
20.C	-0.378450	-2.741080	1.042390
21.C	5.144054	0.837718	1.310045
22.C	-1.687763	-3.088554	1.422320
23.C	0.690792	-3.164364	1.846982
24.C	-1.691533	1.662731	1.826566
25.C	1.460922	4.374799	2.348619
26.C	4.193256	0.183986	2.285420
27.C	-1.950654	2.906391	2.427144
28.C	-2.192380	0.501198	2.431909
29.C	-1.916605	-3.842113	2.578159
30.C	0.463903	-3.913967	3.005427
31.C	1.934899	0.362598	3.075816
32.C	-0.841989	-4.255624	3.374615
33.C	-2.944894	0.577236	3.609135
34.C	-2.692193	2.984794	3.608923
35.C	-3.195734	1.818954	4.200589
36.H	-3.453261	-3.685907	-4.564980
37.H	-2.927924	-1.250859	-4.414902
38.H	2.490519	-3.807063	-4.114143
39.H	-1.924850	3.170580	-4.267180
40.H	1.761707	-2.181802	-4.028853
41.H	-4.312091	3.676795	-3.721718
42.H	0.796454	-3.615265	-3.596723
43.H	-2.512080	-5.262821	-2.876458
44.H	4.322857	-1.522984	-2.456881
45.H	4.824622	-3.221542	-2.354017
46.H	-1.479493	-0.401768	-2.577658
47.H	-0.417440	2.257578	-2.531703
48.H	5.177345	0.837726	-1.999479
49.H	2.924810	-5.431766	-1.555456
50.H	-5.171836	3.234942	-1.421143
51.H	2.742538	4.040647	-1.447565
52.H	-0.230431	5.176248	-1.237463
53.H	-1.056414	-4.416649	-1.060059
54.H	6.568074	0.380823	-0.943582
55.H	4.668238	-2.259691	-0.864032
56.H	5.888686	2.052392	-0.892432
57.H	1.370548	-5.077186	-0.773617
58.H	3.211226	5.246235	-0.218477
59.H	0.378073	6.330523	-0.022970
60.H	2.889002	-4.678792	0.054621

61.H	3.572500	3.532123	0.043216
62.H	-1.026720	5.305952	0.339374
63.H	-3.672542	2.310728	0.312905
64.H	-2.532945	-2.780821	0.804296
65.H	6.158614	0.429354	1.460582
66.H	5.174108	1.930303	1.468101
67.H	1.705706	-2.912402	1.539292
68.H	-1.583411	3.821669	1.964928
69.H	-1.985422	-0.466030	1.972581
70.H	1.848838	5.398436	2.480369
71.H	4.233306	-0.916850	2.202941
72.H	2.211931	3.677942	2.752778
73.H	-2.936853	-4.114506	2.853604
74.H	0.555362	4.276976	2.965202
75.H	0.949530	0.695252	2.736647
76.H	4.478202	0.472311	3.312246
77.H	1.907007	-0.716917	3.290111
78.H	1.307792	-4.243652	3.614497
79.H	2.234954	0.922060	3.975866
80.H	-3.335233	-0.335820	4.061868
81.H	-2.883879	3.956550	4.066785
82.H	-1.020837	-4.849494	4.272909
83.H	-3.782524	1.880514	5.119116
84.I	2.462663	1.334540	-3.485876
85.N	1.548789	-1.792851	-0.825104
86.N	0.636902	2.380960	0.233714
87.Np	2.044391	0.552348	-0.518678
88.O	4.694202	0.556379	-0.027070
89.O	2.861917	0.645788	1.998408
90.P	-0.043160	-1.663433	-0.420429
91.P	-0.758548	1.516481	0.242162
92.Si	2.417670	-2.996127	-1.765719
93.Si	1.148651	4.022868	0.509419

Energy: –546.51288965 eV

Table S10. Final coordinates and energy for a single point energy calculation of geometry optimized **6Np**.

1.C	-0.048534	0.121039	-5.264749
2.C	-3.695479	-2.792197	-4.247650
3.C	-2.329542	-3.000127	-4.031832
4.C	1.041495	2.668623	-3.991770
5.C	2.700376	0.120164	-4.001133
6.C	-4.326914	-1.683210	-3.676438
7.C	-3.084375	3.563983	-3.169775
8.C	-1.594290	-2.094475	-3.265405
9.C	-2.471923	2.311681	-3.070969
10.C	-3.593319	-0.779351	-2.902373
11.C	-2.214922	-0.967335	-2.702850
12.C	-3.333549	4.318289	-2.017780
13.C	6.100848	3.606504	-1.801743

14.C	-2.104387	1.786284	-1.819341
15.C	-2.599567	-5.447148	-1.363063
16.C	3.342754	5.382348	-1.288176
17.C	4.746025	3.190269	-1.336975
18.C	5.353467	0.754143	-1.202150
19.C	3.609165	3.924081	-1.121620
20.C	-1.243305	-5.217395	-1.113421
21.C	-3.553135	-4.525125	-0.918294
22.C	-2.978101	3.804537	-0.766821
23.C	3.100711	1.717025	-0.664324
24.C	-2.375140	2.545842	-0.672045
25.C	-0.841633	-4.076583	-0.412605
26.C	1.263566	3.373884	-0.376554
27.C	-3.153210	-3.384666	-0.217122
28.C	-1.793169	-3.153583	0.050069
29.C	-1.040364	-0.247536	-0.020892
30.C	4.471754	0.220668	2.332644
31.C	-2.507612	-1.450838	2.256469
32.C	2.064979	0.887601	2.196656
33.C	-2.988847	-0.175792	2.586350
34.C	2.672614	-3.031832	2.880123
35.C	-0.125762	1.922497	2.799890
36.C	-2.984967	-2.555499	2.983460
37.C	0.070534	-4.538188	3.129502
38.C	-3.883003	0.002317	3.646905
39.C	-3.883414	-2.383323	4.039542
40.C	3.235397	1.545466	4.077322
41.C	1.963921	2.039872	4.196164
42.C	-4.324655	-1.101070	4.384166
43.C	0.426446	-1.856801	4.572795
44.C	4.403544	1.631054	5.000644
45.C	1.325355	2.847500	5.275599
46.H	0.367200	0.542620	-6.193731
47.H	0.020275	-0.973251	-5.335599
48.H	-1.117239	0.374333	-5.234227
49.H	1.310637	2.922629	-5.029418
50.H	-4.267262	-3.497279	-4.853516
51.H	3.086937	0.398889	-4.994069
52.H	-1.832935	-3.872136	-4.460609
53.H	-3.369799	3.951837	-4.149563
54.H	-5.394382	-1.518163	-3.835361
55.H	2.743492	-0.973575	-3.906886
56.H	-2.290522	1.735328	-3.979322
57.H	0.085615	3.161376	-3.764484
58.H	1.814894	3.095225	-3.336515
59.H	3.378161	0.540783	-3.246384
60.H	-0.527546	-2.255830	-3.103526
61.H	6.388423	3.094146	-2.732436
62.H	-4.096851	0.082367	-2.461582
63.H	-3.810899	5.296876	-2.095917
64.H	6.120111	4.685453	-1.997817
65.H	5.755499	0.734010	-2.223969

66.H	-2.913622	-6.338459	-1.909349
67.H	2.542578	5.567065	-2.021868
68.H	4.243831	5.893459	-1.648516
69.H	-0.491467	-5.926546	-1.465122
70.H	-4.612555	-4.692365	-1.120970
71.H	6.878923	3.389563	-1.053791
72.H	4.825406	-0.190568	-1.022072
73.H	0.885012	4.123296	-1.081412
74.H	6.189294	0.854744	-0.495269
75.H	3.042169	5.860085	-0.342925
76.H	0.623462	2.488184	-0.466528
77.H	0.218170	-3.896202	-0.220499
78.H	-3.181694	4.381625	0.137996
79.H	-3.905797	-2.674315	0.129718
80.H	-2.109305	2.119377	0.296978
81.H	1.204530	3.773077	0.645409
82.H	4.208304	-0.352140	1.436704
83.H	2.977627	-3.554469	1.963012
84.H	5.225387	0.976237	2.068873
85.H	-0.429366	1.396880	1.883225
86.H	0.098522	-5.086279	2.177284
87.H	-2.662332	0.673120	1.984167
88.H	-2.662717	-3.561496	2.713288
89.H	-0.282322	3.004942	2.681227
90.H	3.207193	-2.073296	2.907373
91.H	4.898665	-0.468035	3.072117
92.H	-0.967771	-4.535226	3.487672
93.H	3.009573	-3.623320	3.745614
94.H	0.663116	-5.109504	3.861427
95.H	-0.742648	1.560931	3.631787
96.H	-4.243680	1.003201	3.894559
97.H	-4.242071	-3.253434	4.592663
98.H	5.255806	2.160657	4.547524
99.H	0.950180	-0.888881	4.586038
100.H	-0.649573	-1.662852	4.688787
101.H	0.983159	3.827497	4.907958
102.H	-5.020847	-0.965465	5.213864
103.H	4.756773	0.632224	5.301102
104.H	0.760855	-2.432200	5.450269
105.H	0.452438	2.334740	5.707657
106.H	4.124359	2.169707	5.914242
107.H	2.039010	3.029388	6.088015
108.I	3.304426	-2.647053	-1.075082
109.N	0.325599	0.304666	-2.224100
110.N	4.411541	1.860037	-1.049767
111.N	2.632913	3.006559	-0.716432
112.N	0.271080	-1.899515	1.566118
113.N	3.269929	0.858215	2.857179
114.N	1.280189	1.628246	3.045275
115.Np	1.426362	-0.360648	-0.077766
116.P	-1.211224	0.164810	-1.650632
117.P	-1.215308	-1.639881	0.933372

118.Si	0.948671	0.781513	-3.792316
119.Si	0.803367	-2.794485	2.966761

Energy: −704.34159680 eV

Table S11. Final coordinates and energy for a single point energy calculation of geometry optimized **4U**.

1.C	3.674010	-0.620054	-3.335940
2.C	3.147983	2.393310	-3.569636
3.C	2.183696	-4.147115	-3.130358
4.C	-3.674347	0.218353	-2.749594
5.C	2.020290	-7.105829	-2.480321
6.C	-0.593936	4.218591	-2.617204
7.C	0.089781	3.113471	-2.100694
8.C	1.197212	-6.057498	-1.811219
9.C	-3.167836	-2.683013	-1.906551
10.C	-0.899926	5.303141	-1.790593
11.C	0.432372	-3.929847	-1.374984
12.C	4.935931	1.259344	-1.340019
13.C	0.290549	-6.153592	-0.788523
14.C	0.465140	3.082904	-0.748763
15.C	-0.546828	5.270243	-0.436714
16.C	-0.190772	-7.338213	-0.020843
17.C	-4.741100	-0.833385	-0.079572
18.C	-3.097274	2.455659	0.032061
19.C	-3.803400	3.647934	0.222838
20.C	0.121428	4.162078	0.086664
21.C	-1.060983	-4.505331	0.541092
22.C	0.341530	0.574540	0.875018
23.C	-2.359984	1.890772	1.079947
24.C	3.155751	3.561728	0.955349
25.C	2.684786	2.239629	1.006521
26.C	-3.772353	4.291255	1.461942
27.C	3.299824	1.327006	1.883367
28.C	4.201365	3.971961	1.788843
29.C	-2.308118	2.560632	2.317067
30.C	-3.013984	3.749213	2.506811
31.C	-1.755474	-0.619669	2.387305
32.C	4.342548	1.741914	2.713639
33.C	4.791864	3.065895	2.673863
34.C	-0.825237	-1.554590	2.877139
35.C	-3.008111	-0.516255	3.017912
36.C	-1.138032	-2.351937	3.981595
37.C	-3.321211	-1.322674	4.115917
38.C	-2.386516	-2.240562	4.603120
39.H	4.572838	-0.491280	-3.959388
40.H	2.312060	2.204522	-4.258268
41.H	4.058831	2.514186	-4.176458
42.H	2.835185	-0.865904	-4.001298
43.H	2.373239	-4.873862	-3.928267
44.H	1.793125	-7.187377	-3.554481

45.H	-4.235879	-0.350841	-3.505775
46.H	1.738674	-3.248487	-3.576850
47.H	-0.879872	4.230913	-3.670161
48.H	-2.758264	0.580796	-3.234969
49.H	3.862060	-1.470149	-2.662364
50.H	2.959130	3.348442	-3.058669
51.H	-4.296096	1.079894	-2.465799
52.H	3.096889	-6.899868	-2.381492
53.H	0.333385	2.267005	-2.744241
54.H	3.132648	-3.888950	-2.638351
55.H	-2.293929	-2.846293	-2.553870
56.H	-4.064797	-2.903805	-2.506218
57.H	1.826828	-8.085309	-2.026706
58.H	-1.419553	6.172431	-2.197656
59.H	5.803802	1.268008	-2.018239
60.H	-3.136936	-3.407603	-1.081652
61.H	-3.111440	1.969669	-0.942262
62.H	4.931186	2.209913	-0.790988
63.H	-4.377292	4.073336	-0.601965
64.H	-5.652902	-1.139336	-0.616072
65.H	0.339242	-8.240779	-0.347895
66.H	5.084766	0.446767	-0.614614
67.H	-1.268069	-7.515913	-0.166124
68.H	-0.795074	6.109569	0.214827
69.H	-4.916179	0.179539	0.309607
70.H	-1.343709	-3.451908	0.444155
71.H	-1.970846	-5.119557	0.508225
72.H	2.712620	4.277271	0.262811
73.H	-4.614156	-1.513464	0.774438
74.H	-0.015281	-7.224191	1.059812
75.H	0.379960	4.139534	1.146035
76.H	-4.330140	5.217091	1.613804
77.H	-0.559702	-4.647063	1.508398
78.H	2.995869	0.276934	1.893378
79.H	0.800413	0.350402	1.839659
80.H	4.555917	5.002777	1.740885
81.H	0.149666	-1.676389	2.396035
82.H	-3.744569	0.201315	2.656422
83.H	-1.720086	2.146402	3.137983
84.H	-2.976916	4.252315	3.474866
85.H	4.811181	1.021449	3.385412
86.H	5.607415	3.387377	3.324003
87.H	-0.398189	-3.061373	4.355800
88.H	-4.297693	-1.227356	4.593489
89.H	-2.629423	-2.865880	5.463939
90.I	-0.175466	-1.024675	-4.192073
91.I	2.672709	-2.548243	0.801434
92.N	1.246887	-4.704332	-2.160216
93.N	1.945280	0.716529	-1.340441
94.N	-0.167496	-4.851966	-0.554914
95.N	-1.735361	-0.473124	-0.512606
96.U	0.516204	-1.266010	-1.159631

97.P	1.365074	1.616926	-0.114988
98.P	-1.386253	0.342530	0.856049
99.Si	3.376464	0.961177	-2.359396
100.Si	-3.279581	-0.908238	-1.280402

Energy: −589.14704475 eV

Table S12. Final coordinates and energy for a single point energy calculation of geometry optimized **5U**.

1.C	-2.814376	-3.327769	-3.765774
2.C	-2.536224	-1.959980	-3.676505
3.C	1.798893	-3.166123	-3.523557
4.C	-2.310575	2.994329	-3.262276
5.C	-2.280285	-4.210389	-2.820916
6.C	-3.656935	3.245701	-2.970873
7.C	-1.726955	-1.474403	-2.646243
8.C	-1.462227	2.486764	-2.277069
9.C	4.210759	-2.448103	-1.811610
10.C	-1.466625	-3.726383	-1.792796
11.C	-4.149792	2.977003	-1.691628
12.C	-1.181191	-2.353319	-1.698516
13.C	5.676154	0.986805	-1.003525
14.C	2.367773	-4.713548	-0.925304
15.C	-1.947804	2.220168	-0.983626
16.C	-3.301850	2.464214	-0.703339
17.C	2.804097	4.216794	-0.335926
18.C	-0.053549	5.311617	-0.163833
19.C	-0.258333	-0.040057	-0.094695
20.C	-0.395235	-2.745713	1.056945
21.C	5.176740	0.789319	1.325088
22.C	-1.706167	-3.078782	1.441646
23.C	0.672487	-3.175473	1.860300
24.C	-1.708133	1.656866	1.843198
25.C	1.433407	4.361787	2.365395
26.C	4.202816	0.158782	2.291695
27.C	-1.999296	2.902968	2.425453
28.C	-2.152460	0.490682	2.483763
29.C	-1.939696	-3.822084	2.602596
30.C	0.440125	-3.914652	3.024695
31.C	1.940053	0.377925	3.049964
32.C	-0.867272	-4.239858	3.398996
33.C	-2.879922	0.565036	3.676331
34.C	-2.718603	2.979024	3.620744
35.C	-3.164995	1.808884	4.247132
36.H	-3.449762	-3.706189	-4.568561
37.H	-2.955256	-1.266953	-4.407913
38.H	2.472187	-3.827705	-4.091048
39.H	-1.916670	3.197100	-4.259656
40.H	1.794829	-2.182601	-4.015711
41.H	-4.317970	3.650127	-3.739580
42.H	0.784497	-3.582613	-3.587112

43.H	-2.499390	-5.277996	-2.882872
44.H	4.315308	-1.520971	-2.395653
45.H	4.820894	-3.216966	-2.310382
46.H	-1.514450	-0.406976	-2.564699
47.H	-0.412513	2.295794	-2.515882
48.H	5.208509	0.858296	-1.985549
49.H	2.909786	-5.439403	-1.551538
50.H	-5.198601	3.168948	-1.456632
51.H	2.741425	4.018445	-1.415874
52.H	-0.211975	5.169605	-1.242133
53.H	-1.058940	-4.421956	-1.057308
54.H	6.591774	0.380882	-0.926506
55.H	4.636656	-2.280590	-0.811473
56.H	5.912010	2.050630	-0.847416
57.H	1.345084	-5.093461	-0.790689
58.H	3.182685	5.241873	-0.200331
59.H	0.364058	6.318719	-0.010384
60.H	2.849557	-4.703334	0.063644
61.H	3.565864	3.540730	0.087083
62.H	-1.042311	5.288711	0.317129
63.H	-3.700119	2.260257	0.290834
64.H	-2.550226	-2.765067	0.824164
65.H	6.177746	0.346882	1.465464
66.H	5.244022	1.878768	1.492022
67.H	1.690715	-2.936294	1.548929
68.H	-1.673656	3.823760	1.939252
69.H	-1.921500	-0.477721	2.037160
70.H	1.812440	5.385990	2.506692
71.H	4.206321	-0.942029	2.204259
72.H	2.188996	3.669349	2.766318
73.H	-2.962178	-4.082756	2.882918
74.H	0.526525	4.252218	2.976129
75.H	0.975037	0.773202	2.720384
76.H	4.484388	0.434347	3.322640
77.H	1.860702	-0.707593	3.211526
78.H	1.282131	-4.250008	3.633833
79.H	2.262784	0.878869	3.975899
80.H	-3.227165	-0.351486	4.156819
81.H	-2.938005	3.953290	4.061449
82.H	-1.050296	-4.825212	4.302054
83.H	-3.735299	1.868932	5.175828
84.I	2.501727	1.357519	-3.494981
85.N	1.530067	-1.795556	-0.822574
86.N	0.624849	2.367522	0.258128
87.U	2.045354	0.560348	-0.546590
88.O	4.710522	0.537946	-0.017328
89.O	2.887498	0.666321	1.990015
90.P	-0.062571	-1.663208	-0.407587
91.P	-0.776553	1.506163	0.251501
92.Si	2.403328	-3.005009	-1.744870
93.Si	1.130818	4.011207	0.526577

Energy: −545.42819150 eV

Table S13. Final coordinates and energy for a single point energy calculation of geometry optimized **6U**.

1.C	-0.035049	0.153730	-5.272355
2.C	-3.693264	-2.780967	-4.261174
3.C	-2.325013	-2.990381	-4.060168
4.C	1.055577	2.690866	-3.982476
5.C	2.712799	0.135718	-4.020222
6.C	-4.319746	-1.677622	-3.674029
7.C	-3.090753	3.558141	-3.161642
8.C	-1.582515	-2.091584	-3.292167
9.C	-2.458389	2.315126	-3.072513
10.C	-3.578612	-0.779809	-2.900211
11.C	-2.198031	-0.969095	-2.715197
12.C	-3.372349	4.289588	-2.002257
13.C	6.080324	3.608369	-1.784171
14.C	-2.098410	1.777789	-1.823364
15.C	-2.608964	-5.489239	-1.312722
16.C	3.352558	5.414934	-1.239694
17.C	4.720476	3.203171	-1.323274
18.C	5.299818	0.761536	-1.214316
19.C	3.593584	3.949118	-1.101168
20.C	-1.250178	-5.251104	-1.083932
21.C	-3.559494	-4.562694	-0.871372
22.C	-3.025238	3.763613	-0.753844
23.C	3.046123	1.739865	-0.684051
24.C	-2.398427	2.516026	-0.669411
25.C	-0.843152	-4.098150	-0.406956
26.C	1.241185	3.427619	-0.363904
27.C	-3.154615	-3.411152	-0.191437
28.C	-1.791839	-3.171968	0.055791
29.C	-1.017297	-0.263085	-0.039897
30.C	4.525613	0.257499	2.317349
31.C	-2.510381	-1.441375	2.232113
32.C	2.107225	0.886909	2.190327
33.C	-3.023342	-0.166130	2.509691
34.C	2.687989	-3.004911	2.887665
35.C	-0.097249	1.891675	2.808917
36.C	-2.975365	-2.530445	2.990500
37.C	0.106130	-4.549098	3.104812
38.C	-3.942916	0.028456	3.545473
39.C	-3.895859	-2.341389	4.024195
40.C	3.276298	1.560724	4.069240
41.C	1.998746	2.037971	4.192769
42.C	-4.374791	-1.058316	4.312457
43.C	0.409870	-1.863784	4.557786
44.C	4.447815	1.667692	4.986026
45.C	1.354011	2.834011	5.276947
46.H	0.388857	0.575847	-6.197489
47.H	0.024458	-0.940631	-5.348578
48.H	-1.101482	0.416552	-5.247344
49.H	1.355906	2.954248	-5.009039
50.H	-4.270496	-3.479344	-4.869927

51.H	3.079626	0.392393	-5.026488
52.H	-1.832589	-3.857152	-4.504189
53.H	-3.367466	3.956102	-4.139797
54.H	-5.388675	-1.510983	-3.821132
55.H	2.753244	-0.956319	-3.906416
56.H	-2.254527	1.755590	-3.986580
57.H	0.089807	3.175504	-3.781441
58.H	1.803689	3.116168	-3.298358
59.H	3.405876	0.570539	-3.288487
60.H	-0.514054	-2.253959	-3.141734
61.H	6.353540	3.118443	-2.731488
62.H	-4.077487	0.078748	-2.448151
63.H	-3.867821	5.259786	-2.072538
64.H	6.116648	4.691966	-1.950877
65.H	5.741660	0.767101	-2.219806
66.H	-2.927130	-6.391616	-1.837911
67.H	2.576859	5.630532	-1.990991
68.H	4.270980	5.921528	-1.559779
69.H	-0.500862	-5.964945	-1.431523
70.H	-4.621033	-4.736576	-1.056913
71.H	6.860433	3.358014	-1.048464
72.H	4.758435	-0.183575	-1.082377
73.H	0.866317	4.187545	-1.059691
74.H	6.110258	0.828617	-0.473892
75.H	3.031435	5.873051	-0.291717
76.H	0.590024	2.549769	-0.448022
77.H	0.218666	-3.912281	-0.232094
78.H	-3.254206	4.321855	0.156576
79.H	-3.905647	-2.699661	0.156153
80.H	-2.134720	2.082810	0.297049
81.H	1.199741	3.821348	0.661771
82.H	4.262366	-0.339320	1.436508
83.H	3.009137	-3.539135	1.983090
84.H	5.256432	1.026369	2.027337
85.H	-0.404286	1.362230	1.895593
86.H	0.181820	-5.104525	2.159716
87.H	-2.702363	0.668490	1.884869
88.H	-2.627670	-3.538066	2.761586
89.H	-0.270338	2.971009	2.685175
90.H	3.208062	-2.037896	2.906275
91.H	4.982601	-0.409340	3.059676
92.H	-0.945291	-4.565208	3.421806
93.H	3.022923	-3.578073	3.766304
94.H	0.680555	-5.102195	3.864611
95.H	-0.705737	1.525617	3.645928
96.H	-4.332569	1.028321	3.748003
97.H	-4.245176	-3.199226	4.601982
98.H	5.299449	2.182400	4.514442
99.H	0.921450	-0.889521	4.574845
100.H	-0.669187	-1.684082	4.666122
101.H	0.981551	3.803399	4.910232
102.H	-5.092892	-0.910162	5.121039

103.H	4.800732	0.677340	5.313623
104.H	0.745693	-2.435702	5.436886
105.H	0.500347	2.301033	5.723166
106.H	4.174064	2.233526	5.884631
107.H	2.073664	3.037144	6.079090
108.I	3.260680	-2.677005	-1.081559
109.N	0.344381	0.317221	-2.229544
110.N	4.365274	1.872050	-1.062827
111.N	2.599844	3.040961	-0.719732
112.N	0.279901	-1.901169	1.551439
113.N	3.316535	0.870408	2.850997
114.N	1.313375	1.616743	3.046139
115.U	1.442512	-0.359441	-0.056351
116.P	-1.192186	0.163609	-1.666645
117.P	-1.212842	-1.648043	0.918473
118.Si	0.964970	0.803380	-3.795933
119.Si	0.814685	-2.795072	2.955922

Energy: −703.49454966 eV

Table S14. Final coordinates and energy for a single point energy calculation of geometry optimized **4Ce**.

1.C	2.142813	1.961351	-4.079856
2.C	3.534023	2.033725	-3.966477
3.C	-0.270371	-2.540865	-3.881371
4.C	4.187693	-1.771723	-2.776384
5.C	1.345517	1.945069	-2.931115
6.C	4.130610	2.063128	-2.699577
7.C	-0.973479	4.519418	-2.658264
8.C	5.334630	-1.678475	-1.984337
9.C	2.921966	-1.643110	-2.194911
10.C	0.716559	-4.456146	-1.699854
11.C	-2.190907	-3.603669	-1.769925
12.C	1.935780	1.992297	-1.658157
13.C	3.338858	2.028093	-1.550575
14.C	-3.331627	3.602510	-0.914001
15.C	5.212552	-1.440245	-0.608860
16.C	2.788797	-1.429661	-0.817877
17.C	3.949523	-1.311677	-0.029352
18.C	-5.660239	0.729631	-0.055249
19.C	-1.101880	5.186672	0.338864
20.C	-4.101115	-1.082973	0.591912
21.C	2.449463	4.138268	0.680918
22.C	0.836434	0.375372	0.616763
23.C	1.552164	3.118057	1.037131
24.C	-6.227661	-1.224989	1.451431
25.C	-7.652860	-0.857022	1.723143
26.C	2.039242	-3.463831	1.458479
27.C	1.253903	-2.297755	1.466125
28.C	2.936683	5.020550	1.650497
29.C	-3.124334	-3.050844	1.753917

30.C	-5.512932	-2.286484	1.947670
31.C	1.117628	3.024346	2.372270
32.C	2.051271	-4.321026	2.562793
33.C	-5.963398	-3.374571	2.871732
34.C	0.472370	-2.014917	2.601297
35.C	2.522945	4.903263	2.980651
36.C	1.604201	3.909067	3.335824
37.C	1.279071	-4.026266	3.691011
38.C	0.490266	-2.870871	3.706121
39.H	1.671281	1.925356	-5.062779
40.H	4.156021	2.061644	-4.863051
41.H	-0.638901	-3.388772	-4.481827
42.H	-0.887608	-1.663816	-4.124757
43.H	0.760478	-2.339700	-4.205918
44.H	4.273035	-1.943003	-3.850303
45.H	-1.283677	3.834275	-3.461140
46.H	0.260152	1.897805	-3.016301
47.H	-1.466423	5.489296	-2.835142
48.H	6.322820	-1.784194	-2.435469
49.H	2.032383	-1.700930	-2.817134
50.H	5.216431	2.107936	-2.605400
51.H	0.438145	-5.282949	-2.374555
52.H	0.112395	4.677675	-2.745577
53.H	-2.435763	-4.339588	-2.553019
54.H	1.778835	-4.230175	-1.876726
55.H	-2.944724	-2.802966	-1.830574
56.H	-3.679217	2.888572	-1.675578
57.H	-3.822188	4.569577	-1.114065
58.H	0.610961	-4.821013	-0.667088
59.H	-2.292556	-4.113126	-0.800702
60.H	-5.062700	0.791134	-0.974136
61.H	3.813115	2.035898	-0.568880
62.H	6.104081	-1.358233	0.014695
63.H	-3.665608	3.268722	0.081636
64.H	2.772694	4.249303	-0.353755
65.H	-6.719271	0.762291	-0.335248
66.H	-1.717538	6.070898	0.102967
67.H	-0.050731	5.508782	0.368437
68.H	-5.430472	1.581734	0.600659
69.H	2.652909	-3.703127	0.590204
70.H	3.864755	-1.126834	1.043110
71.H	-7.938062	0.075721	1.224594
72.H	-8.344835	-1.641644	1.381306
73.H	-2.260303	-2.788646	1.138391
74.H	-1.380934	4.842117	1.346334
75.H	3.640290	5.802330	1.361340
76.H	-3.376207	-4.100647	1.559328
77.H	1.092985	0.466815	1.673042
78.H	-6.771254	-3.975164	2.425808
79.H	-7.821211	-0.713369	2.801189
80.H	2.670990	-5.218429	2.540178
81.H	-0.159688	-1.123579	2.635206

82.H	-2.853522	-2.924763	2.809963
83.H	-5.144218	-4.059057	3.122186
84.H	0.368946	2.281693	2.657981
85.H	-6.343688	-2.959134	3.817150
86.H	2.907404	5.589648	3.737067
87.H	1.256006	3.823245	4.366133
88.H	1.294424	-4.692182	4.555604
89.H	-0.113445	-2.626238	4.581466
90.Ce	-1.786697	0.140749	-0.247271
91.I	-3.027644	0.308189	-3.137636
92.I	-2.330884	0.982451	2.795835
93.N	-0.115887	-1.586189	-0.998688
94.N	-0.686012	2.299952	-0.610743
95.N	-5.351768	-0.526612	0.616566
96.N	-4.232850	-2.178616	1.401420
97.P	0.843758	1.952501	-0.189410
98.P	1.139034	-1.220515	-0.028419
99.Si	-0.426487	-2.986272	-2.049808
100.Si	-1.460850	3.859645	-0.954625

Energy: −588.15415491 eV

Table S15. Final coordinates and energy for a single point energy calculation of geometry optimized **5Ce**.

1.C	0.697623	-1.035128	-5.036294
2.C	0.536350	3.429408	-4.151900
3.C	3.543276	-0.826166	-3.894586
4.C	-0.407350	4.291654	-3.577575
5.C	0.985833	2.312408	-3.442861
6.C	1.801396	-3.303871	-3.354373
7.C	-2.573587	-0.675027	-2.531001
8.C	-2.343047	-4.349677	-2.229926
9.C	-3.037101	-3.006284	-2.205045
10.C	-0.903077	4.025295	-2.297696
11.C	0.491223	2.035721	-2.157536
12.C	-0.458674	2.899834	-1.594671
13.C	-0.841610	-5.727754	-0.980880
14.C	3.330461	2.372034	-0.959785
15.C	4.652991	2.675137	-0.615803
16.C	2.820835	1.078710	-0.760463
17.C	5.478274	1.692810	-0.060796
18.C	3.656921	0.098311	-0.194377
19.C	4.973523	0.403018	0.153150
20.C	0.136672	0.226863	0.143970
21.C	-4.709107	1.275123	1.013625
22.C	-3.535136	0.525706	1.135429
23.C	-4.685789	2.655307	1.242394
24.C	-2.321128	1.139683	1.481221
25.C	-3.481148	3.278403	1.591330
26.C	-2.307088	2.527841	1.706506
27.C	-1.910154	-4.222523	2.354357

28.C	1.476373	0.741334	3.057092
29.C	0.072992	0.734472	3.047075
30.C	-3.325502	-1.913149	3.763097
31.C	2.174668	1.139798	4.199993
32.C	-0.622552	1.141718	4.199068
33.C	-0.378330	-2.570483	4.380319
34.C	1.476624	1.545675	5.342446
35.C	0.076416	1.548744	5.339196
36.H	1.103273	-1.539280	-5.929364
37.H	0.627879	0.039231	-5.260168
38.H	0.924988	3.631537	-5.151222
39.H	-0.326159	-1.407700	-4.877733
40.H	3.920468	-1.314857	-4.807887
41.H	2.231240	-3.769570	-4.255867
42.H	-0.753297	5.168919	-4.127661
43.H	3.601825	0.261488	-4.052889
44.H	1.734750	1.659628	-3.890754
45.H	-3.023095	-0.694499	-3.536024
46.H	-3.675885	-2.917448	-3.101861
47.H	-1.796240	-4.487740	-3.180306
48.H	0.780280	-3.709872	-3.248205
49.H	4.226421	-1.071894	-3.068381
50.H	2.392611	-3.638367	-2.487454
51.H	-3.097458	-5.151211	-2.136660
52.H	-1.732888	0.024781	-2.510532
53.H	-3.325402	-0.373137	-1.786218
54.H	-0.345595	-6.037258	-1.913570
55.H	-1.636487	4.693528	-1.842626
56.H	-3.674106	-2.903524	-1.307725
57.H	2.699079	3.149271	-1.392106
58.H	5.036064	3.682469	-0.786560
59.H	-1.625743	-6.452392	-0.712696
60.H	-0.848518	2.678883	-0.600301
61.H	-0.096356	-5.658766	-0.180968
62.H	6.510747	1.929118	0.202561
63.H	3.278678	-0.913355	-0.029158
64.H	5.607821	-0.371160	0.588164
65.H	-5.646433	0.779522	0.753612
66.H	-3.547922	-0.554110	0.985866
67.H	-5.603182	3.240683	1.159291
68.H	-2.622769	-4.280543	1.517279
69.H	-3.458096	4.352508	1.782674
70.H	-0.948789	-4.652025	2.028182
71.H	-1.376539	3.022586	1.990091
72.H	2.015646	0.442006	2.157398
73.H	-2.290725	-4.865002	3.165099
74.H	-4.152587	-2.002421	3.041904
75.H	-3.303232	-0.870167	4.113330
76.H	0.593982	-2.880210	3.966007
77.H	3.265610	1.139463	4.192862
78.H	-1.713321	1.149732	4.206512
79.H	-3.569538	-2.548810	4.630085

80.H	-0.233538	-1.615340	4.907623
81.H	-0.687016	-3.325594	5.122726
82.H	2.022309	1.864803	6.232350
83.H	-0.472461	1.870741	6.225514
84.Ce	-0.045682	-2.147613	-0.388234
85.I	2.286800	-3.838559	0.831961
86.N	1.117654	-0.789008	-2.048955
87.N	-1.200073	-1.460205	1.650904
88.O	-2.031884	-1.979372	-2.213964
89.O	-1.419408	-4.410842	-1.129721
90.P	1.090973	0.598780	-1.173280
91.P	-0.777528	0.128664	1.535202
92.Si	1.786038	-1.413712	-3.528576
93.Si	-1.671639	-2.461175	3.012277

Energy: −544.68353825 eV

Table S16. Final coordinates and energy for a single point energy calculation of geometry optimized **6Ce**.

1.C	-0.580992	-0.703151	-4.771780
2.C	2.154303	-1.923480	-4.466717
3.C	-5.771553	-2.203170	-4.037133
4.C	-4.217350	0.353048	-3.634161
5.C	-0.281364	-3.458041	-3.508749
6.C	-4.654304	-2.055414	-3.057947
7.C	4.784613	0.626567	-2.827408
8.C	5.905300	0.013656	-2.256488
9.C	-4.091818	-2.963858	-2.198569
10.C	3.502351	0.300690	-2.380078
11.C	-4.406223	-4.404217	-1.961813
12.C	-2.957756	-0.972587	-1.931220
13.C	5.737372	-0.913762	-1.222387
14.C	3.522629	4.051522	-1.162932
15.C	3.321902	-0.644689	-1.357244
16.C	4.833459	3.852316	-0.715998
17.C	2.355177	-3.768435	-1.015396
18.C	4.454504	-1.236194	-0.770155
19.C	2.406210	-5.098903	-0.591874
20.C	2.470132	3.333386	-0.589150
21.C	-2.231076	-2.867170	-0.500540
22.C	1.763504	-2.778601	-0.211277
23.C	-4.804741	1.769186	-0.131206
24.C	5.086865	2.924081	0.300192
25.C	1.857614	-5.465717	0.643554
26.C	2.713984	2.407660	0.439142
27.C	-2.165859	4.423283	0.618874
28.C	0.982654	-0.066744	0.466741
29.C	4.035168	2.202610	0.872262
30.C	1.230642	-3.156509	1.029303
31.C	-3.006512	0.489776	1.029079
32.C	1.269062	-4.489258	1.454587

33.C	0.470495	5.182727	1.884005
34.C	-4.982599	0.794332	2.179685
35.C	-6.366092	1.303841	2.413613
36.C	-1.800690	-0.812556	2.775505
37.C	-4.152027	0.057079	2.983303
38.C	1.719586	1.373586	2.930134
39.C	-1.473610	3.337988	3.369342
40.C	2.222505	2.489146	3.621814
41.C	1.494431	0.188183	3.644298
42.C	-4.355353	-0.493292	4.356025
43.C	2.458582	2.431261	4.997437
44.C	1.721190	0.126583	5.023632
45.C	2.195694	1.252450	5.706294
46.H	-0.575521	-1.180993	-5.765597
47.H	1.977909	-2.485980	-5.399152
48.H	-5.449602	-1.973693	-5.064572
49.H	-0.213426	0.328007	-4.882852
50.H	-4.161098	0.147843	-4.710877
51.H	2.592197	-0.953114	-4.742254
52.H	-0.177558	-3.947715	-4.491359
53.H	-6.146610	-3.233731	-4.031218
54.H	-1.625346	-0.647376	-4.431119
55.H	-6.618673	-1.539818	-3.800962
56.H	2.915342	-2.468813	-3.889754
57.H	-5.215940	0.747674	-3.400086
58.H	4.907745	1.357936	-3.627575
59.H	-3.457621	1.104681	-3.388446
60.H	-1.355940	-3.408160	-3.276756
61.H	-5.224068	-4.725926	-2.617918
62.H	6.906577	0.260107	-2.614948
63.H	0.205471	-4.100893	-2.759986
64.H	2.629848	0.769282	-2.835593
65.H	-3.540099	-5.050045	-2.173365
66.H	3.314876	4.770134	-1.957444
67.H	2.790619	-3.497016	-1.976859
68.H	5.656350	4.416753	-1.159342
69.H	2.877439	-5.851207	-1.226721
70.H	-4.717569	-4.594366	-0.922836
71.H	6.606859	-1.392659	-0.768374
72.H	1.447303	3.495382	-0.932419
73.H	-4.016078	1.907001	-0.876851
74.H	-2.025179	-3.916672	-0.734997
75.H	-5.635561	1.214690	-0.588435
76.H	-1.269176	-2.341759	-0.482327
77.H	-1.844832	4.698170	-0.397090
78.H	4.335789	-1.963332	0.034715
79.H	-5.160338	2.759331	0.181304
80.H	6.107483	2.758358	0.648809
81.H	-2.717271	-2.800977	0.482072
82.H	-2.987875	3.698529	0.523328
83.H	1.898900	-6.505168	0.974666
84.H	-2.573831	5.321477	1.110484

85.H	1.070017	5.367081	0.980679
86.H	4.243860	1.484726	1.667111
87.H	-7.086361	0.896487	1.686927
88.H	0.795763	-2.376330	1.655429
89.H	-0.105224	6.098825	2.098955
90.H	-6.412502	2.401845	2.347928
91.H	0.851143	-4.765204	2.424985
92.H	-0.986185	-0.719947	2.044628
93.H	1.163244	5.033483	2.725096
94.H	2.445428	3.405949	3.076399
95.H	-6.709724	1.019966	3.415613
96.H	-2.027223	-1.874904	2.944110
97.H	-2.298887	2.613813	3.279289
98.H	1.156899	-0.689935	3.093172
99.H	-1.469459	-0.359997	3.718164
100.H	-1.878621	4.253508	3.833094
101.H	-0.721714	2.917620	4.054853
102.H	-4.276797	-1.591740	4.374239
103.H	-5.351936	-0.227865	4.728435
104.H	-3.617080	-0.096207	5.069144
105.H	2.851376	3.307682	5.515772
106.H	1.537341	-0.803073	5.566847
107.H	2.374837	1.208405	6.782285
108.Ce	-0.986606	0.720602	-0.834053
109.I	-1.234997	2.986222	-3.021888
110.N	-3.948401	-0.859159	-2.872006
111.N	0.547950	-0.917194	-2.025423
112.N	-3.070206	-2.277942	-1.533520
113.N	-0.078943	2.324101	0.874938
114.N	-4.262242	1.037711	1.003479
115.N	-2.966325	-0.112438	2.258398
116.P	1.615568	-1.020905	-0.782190
117.P	1.291701	1.465206	1.130841
118.Si	0.482746	-1.719557	-3.587279
119.Si	-0.745712	3.739178	1.664081

Energy: −702.46114925 eV

Table S17. Final coordinates and energy for a single point energy calculation of geometry optimized **4Pm**.

1.C	2.158642	1.951814	-4.068778
2.C	3.549251	2.028929	-3.951553
3.C	-0.292632	-2.530232	-3.882126
4.C	4.148356	-1.750601	-2.783419
5.C	1.358639	1.931028	-2.922178
6.C	4.142310	2.057618	-2.682966
7.C	-0.927715	4.525101	-2.662732
8.C	5.298054	-1.658769	-1.995325
9.C	2.884690	-1.630036	-2.196192
10.C	0.695321	-4.434328	-1.695053
11.C	-2.216908	-3.609328	-1.790045

12.C	1.944921	1.978153	-1.647130
13.C	3.347682	2.017382	-1.536023
14.C	-3.321702	3.613835	-0.971906
15.C	5.180281	-1.430312	-0.617925
16.C	2.755322	-1.426995	-0.817302
17.C	3.919000	-1.310530	-0.032709
18.C	-5.566991	0.746832	0.002010
19.C	-1.108812	5.164669	0.340056
20.C	-4.024841	-1.095045	0.602256
21.C	2.464223	4.115405	0.702781
22.C	0.824254	0.372984	0.631085
23.C	1.547865	3.107886	1.046173
24.C	-6.154122	-1.244906	1.451901
25.C	-7.580762	-0.882935	1.723788
26.C	2.033125	-3.459346	1.462680
27.C	1.228503	-2.306596	1.465991
28.C	2.954970	4.986884	1.680439
29.C	-3.053823	-3.080473	1.736284
30.C	-5.443300	-2.316467	1.931954
31.C	1.095858	3.019436	2.375962
32.C	2.056751	-4.314269	2.568612
33.C	-5.900359	-3.417475	2.836968
34.C	0.437250	-2.036563	2.597399
35.C	2.524985	4.872718	3.005672
36.C	1.584998	3.893875	3.347305
37.C	1.275621	-4.031322	3.693606
38.C	0.465957	-2.890430	3.703472
39.H	1.689820	1.915737	-5.052960
40.H	4.173499	2.060807	-4.846427
41.H	-0.644529	-3.388052	-4.478596
42.H	-0.921843	-1.664713	-4.135586
43.H	0.736546	-2.315146	-4.202824
44.H	4.229858	-1.913736	-3.858804
45.H	-1.228019	3.850673	-3.478353
46.H	0.273793	1.881385	-3.009642
47.H	-1.414534	5.498594	-2.835808
48.H	6.284708	-1.756942	-2.451117
49.H	1.993123	-1.685847	-2.815316
50.H	5.227695	2.105630	-2.585950
51.H	0.423712	-5.261765	-2.371814
52.H	0.159905	4.679586	-2.730838
53.H	-2.440936	-4.347554	-2.577134
54.H	1.756919	-4.202677	-1.867653
55.H	-2.977038	-2.816285	-1.857861
56.H	-3.662703	2.891696	-1.727825
57.H	-3.793246	4.584344	-1.199006
58.H	0.587845	-4.801304	-0.663147
59.H	-2.324697	-4.120897	-0.822971
60.H	-5.018451	0.807716	-0.946388
61.H	3.819523	2.024130	-0.553253
62.H	6.073943	-1.348475	0.002608
63.H	-3.682347	3.302189	0.021232

64.H	2.800371	4.224398	-0.327720
65.H	-6.636474	0.827976	-0.217168
66.H	-1.720841	6.051036	0.103351
67.H	-0.058430	5.485812	0.392160
68.H	-5.268206	1.572324	0.663707
69.H	2.653752	-3.689664	0.597092
70.H	3.838229	-1.132518	1.041021
71.H	-7.879829	0.032829	1.202327
72.H	-8.265571	-1.683519	1.405920
73.H	-2.186274	-2.800832	1.133698
74.H	-1.406533	4.807954	1.337997
75.H	3.675188	5.757260	1.401857
76.H	-3.302968	-4.126514	1.519419
77.H	1.082567	0.459320	1.686980
78.H	-6.720437	-3.996787	2.385399
79.H	-7.745385	-0.712474	2.798550
80.H	2.692653	-5.200347	2.549693
81.H	-0.209079	-1.155357	2.626562
82.H	-2.794523	-2.975284	2.797458
83.H	-5.088689	-4.118405	3.064802
84.H	0.332955	2.287377	2.650672
85.H	-6.266816	-3.017311	3.794412
86.H	2.913504	5.549648	3.768457
87.H	1.223393	3.811376	4.373227
88.H	1.299804	-4.695237	4.559441
89.H	-0.145669	-2.655928	4.576136
90.Pm	-1.789036	0.165254	-0.255490
91.I	-3.031848	0.289144	-3.107415
92.I	-2.352833	1.005847	2.767821
93.N	-0.154350	-1.573805	-0.989930
94.N	-0.679865	2.289979	-0.623327
95.N	-5.273789	-0.533742	0.632971
96.N	-4.160877	-2.202609	1.392512
97.P	0.842991	1.942844	-0.184258
98.P	1.105776	-1.223133	-0.024466
99.Si	-0.457447	-2.970989	-2.048856
100.Si	-1.447990	3.850977	-0.973680

Energy: −589.23163282 eV

Table S18. Final coordinates and energy for a single point energy calculation of geometry optimized **5Pm**.

1.C	0.711023	-1.064662	-5.048036
2.C	0.525242	3.438623	-4.137170
3.C	3.546727	-0.833786	-3.886570
4.C	-0.390267	4.316498	-3.543607
5.C	0.963729	2.309311	-3.440619
6.C	1.819435	-3.319106	-3.338984
7.C	-2.533454	-0.664865	-2.551449
8.C	-2.267051	-4.331323	-2.215552
9.C	-2.978257	-2.997251	-2.208335
10.C	-0.870095	4.054021	-2.257146

11.C	0.487061	2.037454	-2.147284
12.C	-0.436294	2.917328	-1.565612
13.C	-0.800681	-5.703569	-0.924632
14.C	3.327725	2.351543	-0.966036
15.C	4.654138	2.646989	-0.629864
16.C	2.811064	1.062133	-0.758324
17.C	5.474662	1.662576	-0.073345
18.C	3.642497	0.079200	-0.190086
19.C	4.962466	0.377834	0.151118
20.C	0.126180	0.205806	0.133371
21.C	-4.712236	1.243674	1.000350
22.C	-3.535540	0.499144	1.119274
23.C	-4.696281	2.621753	1.242901
24.C	-2.326652	1.116394	1.476285
25.C	-3.495951	3.247640	1.602161
26.C	-2.318820	2.502069	1.714132
27.C	-1.903460	-4.246624	2.380383
28.C	1.486007	0.698764	3.032850
29.C	0.082813	0.718077	3.031573
30.C	-3.306246	-1.916084	3.774288
31.C	2.198493	1.094940	4.166819
32.C	-0.598545	1.145973	4.184411
33.C	-0.351749	-2.563609	4.369465
34.C	1.514977	1.522256	5.310418
35.C	0.114975	1.549352	5.316235
36.H	1.118283	-1.579036	-5.934690
37.H	0.647766	0.008219	-5.282797
38.H	0.902076	3.637570	-5.141798
39.H	-0.315915	-1.430114	-4.891040
40.H	3.933883	-1.324009	-4.795112
41.H	2.251160	-3.795314	-4.234134
42.H	-0.727599	5.201879	-4.084140
43.H	3.601035	0.254042	-4.048311
44.H	1.691099	1.642860	-3.903168
45.H	-2.958279	-0.701711	-3.566692
46.H	-3.602599	-2.920786	-3.116038
47.H	-1.700758	-4.464191	-3.154896
48.H	0.800859	-3.728798	-3.221700
49.H	4.225349	-1.073375	-3.054269
50.H	2.412006	-3.635521	-2.465983
51.H	-3.013176	-5.141780	-2.135380
52.H	-1.700827	0.043641	-2.520421
53.H	-3.306331	-0.361271	-1.829179
54.H	-0.290428	-6.025752	-1.845339
55.H	-1.583834	4.733012	-1.788195
56.H	-3.632179	-2.899203	-1.322888
57.H	2.699441	3.130346	-1.400110
58.H	5.043217	3.650632	-0.808963
59.H	-1.597914	-6.417203	-0.665266
60.H	-0.815379	2.701302	-0.565691
61.H	-0.069473	-5.632002	-0.112494
62.H	6.509834	1.892761	0.182726

63.H	3.258450	-0.929751	-0.019767
64.H	5.593063	-0.398200	0.587473
65.H	-5.646470	0.746981	0.731527
66.H	-3.541673	-0.578836	0.959139
67.H	-5.616620	3.203875	1.162424
68.H	-2.617367	-4.313792	1.545026
69.H	-3.479257	4.319897	1.803904
70.H	-0.941855	-4.676200	2.056021
71.H	-1.391755	2.997954	2.005800
72.H	2.015080	0.379076	2.134709
73.H	-2.281764	-4.879376	3.199863
74.H	-4.140310	-2.009857	3.061715
75.H	-3.275285	-0.868875	4.111181
76.H	0.622543	-2.850540	3.943828
77.H	3.288954	1.074810	4.153144
78.H	-1.688581	1.170737	4.199950
79.H	-3.545483	-2.539530	4.651294
80.H	-0.223555	-1.606386	4.897250
81.H	-0.635536	-3.326430	5.113595
82.H	2.072219	1.838228	6.194916
83.H	-0.421844	1.885939	6.204359
84.Pm	-0.062144	-2.134982	-0.397897
85.I	2.249517	-3.798842	0.838166
86.N	1.107174	-0.795731	-2.062840
87.N	-1.202366	-1.483909	1.641278
88.O	-1.986174	-1.958608	-2.205420
89.O	-1.364462	-4.382369	-1.097588
90.P	1.085910	0.582030	-1.188547
91.P	-0.785282	0.100959	1.534683
92.Si	1.790097	-1.431688	-3.530084
93.Si	-1.660764	-2.478639	3.014170

Energy: −545.84775623 eV

Table S19. Final coordinates and energy for a single point energy calculation of geometry optimized **6Pm**.

1.C	-0.561028	-0.675175	-4.787529
2.C	2.164760	-1.926458	-4.471440
3.C	-5.749706	-2.187347	-4.039007
4.C	-4.183366	0.353381	-3.637742
5.C	-0.283647	-3.433086	-3.518109
6.C	-4.638922	-2.046106	-3.051526
7.C	4.808258	0.596617	-2.812938
8.C	5.920349	-0.018802	-2.228282
9.C	-4.090338	-2.954974	-2.184194
10.C	3.520478	0.283375	-2.372054
11.C	-4.419815	-4.391446	-1.944518
12.C	-2.936380	-0.973957	-1.923862
13.C	5.738558	-0.935530	-1.186842
14.C	3.525289	4.061369	-1.145863
15.C	3.326026	-0.652441	-1.342806
16.C	4.831404	3.872158	-0.681283

17.C	2.335478	-3.776500	-1.015407
18.C	4.450151	-1.245934	-0.741820
19.C	2.387895	-5.103666	-0.581614
20.C	2.471514	3.330546	-0.590791
21.C	-2.227644	-2.872109	-0.488440
22.C	1.751553	-2.779586	-0.214609
23.C	-4.786335	1.766954	-0.162617
24.C	5.079753	2.939648	0.332269
25.C	1.850799	-5.459675	0.662119
26.C	2.709991	2.402965	0.437187
27.C	-2.170674	4.430847	0.597768
28.C	0.977835	-0.074326	0.466991
29.C	4.026984	2.205800	0.885901
30.C	1.227490	-3.147637	1.032814
31.C	-2.982669	0.511591	1.012168
32.C	1.270095	-4.476268	1.470001
33.C	0.473311	5.193949	1.838176
34.C	-4.964949	0.801972	2.153195
35.C	-6.353256	1.300814	2.381663
36.C	-1.771330	-0.774325	2.765158
37.C	-4.130530	0.075471	2.962683
38.C	1.713769	1.361739	2.922591
39.C	-1.458183	3.358437	3.349521
40.C	2.196951	2.477208	3.627998
41.C	1.522066	0.160780	3.619524
42.C	-4.332473	-0.469779	4.337633
43.C	2.447622	2.402357	5.000118
44.C	1.764718	0.081274	4.995100
45.C	2.220618	1.205994	5.691501
46.H	-0.555437	-1.149847	-5.783022
47.H	1.989056	-2.498366	-5.398186
48.H	-5.412421	-1.981786	-5.066683
49.H	-0.187520	0.354304	-4.893123
50.H	-4.127168	0.141438	-4.713083
51.H	2.615539	-0.964527	-4.755194
52.H	-0.195503	-3.919696	-4.503644
53.H	-6.147005	-3.209348	-4.020203
54.H	-1.605528	-0.613437	-4.449546
55.H	-6.584178	-1.501897	-3.823242
56.H	2.916197	-2.473334	-3.882867
57.H	-5.180477	0.753195	-3.407105
58.H	4.942040	1.319178	-3.619268
59.H	-3.421408	1.103354	-3.394389
60.H	-1.354477	-3.378665	-3.270423
61.H	-5.251256	-4.700748	-2.589250
62.H	6.925968	0.216496	-2.582028
63.H	0.209824	-4.081284	-2.778229
64.H	2.654339	0.753217	-2.838236
65.H	-3.565947	-5.048773	-2.169467
66.H	3.322067	4.783378	-1.938480
67.H	2.764199	-3.513679	-1.982227
68.H	5.654621	4.448413	-1.108173

69.H	2.851613	-5.862141	-1.214481
70.H	-4.718962	-4.579227	-0.901939
71.H	6.601277	-1.416274	-0.722136
72.H	1.451858	3.485654	-0.946470
73.H	-3.992743	1.914619	-0.901061
74.H	-2.018153	-3.918711	-0.733175
75.H	-5.604188	1.196529	-0.623222
76.H	-1.270225	-2.341055	-0.461441
77.H	-1.856666	4.702551	-0.420585
78.H	4.320606	-1.965994	0.067661
79.H	-5.161267	2.751314	0.144869
80.H	6.096791	2.781682	0.694442
81.H	-2.717325	-2.817916	0.493032
82.H	-2.992569	3.705695	0.508715
83.H	1.894841	-6.496243	1.001798
84.H	-2.575123	5.330554	1.090872
85.H	1.040369	5.395917	0.917565
86.H	4.231237	1.485832	1.679988
87.H	-7.065170	0.897109	1.645064
88.H	0.796749	-2.362074	1.654851
89.H	-0.100315	6.102772	2.086012
90.H	-6.406037	2.399194	2.327664
91.H	0.861540	-4.742955	2.446880
92.H	-0.960306	-0.685670	2.029869
93.H	1.196183	5.038830	2.651915
94.H	2.393336	3.407847	3.096710
95.H	-6.702965	1.003591	3.377616
96.H	-1.994183	-1.835359	2.945962
97.H	-2.282062	2.630844	3.270941
98.H	1.199399	-0.714955	3.056318
99.H	-1.440644	-0.309947	3.702259
100.H	-1.862389	4.275713	3.809390
101.H	-0.699883	2.944729	4.032196
102.H	-4.244076	-1.567242	4.362016
103.H	-5.332099	-0.211041	4.706602
104.H	-3.599194	-0.062367	5.050157
105.H	2.824369	3.278980	5.529694
106.H	1.608897	-0.861639	5.523250
107.H	2.412703	1.148068	6.764492
108.Pm	-0.975669	0.721105	-0.839191
109.I	-1.288116	2.971272	-2.992352
110.N	-3.921976	-0.856322	-2.868541
111.N	0.557274	-0.898063	-2.038357
112.N	-3.064737	-2.274759	-1.518599
113.N	-0.083148	2.323312	0.857540
114.N	-4.241341	1.047449	0.979212
115.N	-2.941091	-0.085171	2.242190
116.P	1.609678	-1.022681	-0.791850
117.P	1.281449	1.465198	1.123910
118.Si	0.491353	-1.698920	-3.600231
119.Si	-0.744468	3.748657	1.634760

Energy: −703.54428584 eV

S10. References

1. NuDat 2.8, Chart of the Nuclides. https://www.nndc.bnl.gov/nudat2/.
2. Kasani, A.; Kamalesh, R. P.; McDonald, R.; Cavell, R. G. $[Ph_2P(NSiMe_3)]_2CLi_2$: A Dilithium Dianionic Methanide Salt with an Unusual Li_4C_2 Cluster Structure. *Angew. Chem. Int. Ed.* **1999**, *38*, 1483-1484.
3. Ong, C. M.; Stephan, D. W. Lithiations of Bis-diphenyl-*N*-trimethylsilylphosphiniminomethane: An X-ray Structure of a 1,1-Dilithiomethane Derivative. *J. Am. Chem. Soc.* **1999**, *121*, 2939-2940.
4. Wooles, A. J.; Gregson, M.; Cooper, O. J.; Middleton-Gear, A.; Mills, D. P.; Lewis, W.; Blake, A. J.; Liddle, S. T. Group 1 Bis(iminophosphorano)methanides, Part 1: *N*-Alkyl and Silyl Derivatives of the Sterically Demanding Methanes $H_2C(PPh_2NR)_2$ (R = Adamantyl and Trimethylsilyl). *Organometallics* **2011**, *30*, 5314-5325.
5. Reilly, S. D.; Brown, J. L.; Scott, B. L.; Gaunt, A. J. Synthesis and characterization of $NpCl_4(DME)_2$ and $PuCl_4(DME)_2$ neutral transuranic An(IV) starting materials. *Dalton Trans.* **2014**, *43*, 1498-1501.
6. Goodwin, C. A. P.; Gaunt, A. J.; Janicke, M. T.; Scott, B. L. $[AnI_3(THF)_4]$ (An = Np, Pu) preparation bypassing An^0 metal precursors: access to Np^{3+}/Pu^{3+} nonaqueous and organometallic complexes. *J. Am. Chem. Soc.* **2021**, *143*, 20680-20696.
7. Talavera, G.; Pena, J.; Alcarazo, M. Dihalo(imidazolium)sulfuranes: A Versatile Platform for the Synthesis of New Electrophilic Group-Transfer Reagents. *J. Am. Chem. Soc.* **2015**, *137*, 8704-8707.
8. Ansell, M. B.; Roberts, D. E.; Cloke, F. G.; Navarro, O.; Spencer, J. Synthesis of an $[(NHC)_2Pd(SiMe_3)_2]$ Complex and Catalytic *cis*-Bis(silyl)ations of Alkynes with Unactivated Disilanes. *Angew. Chem. Int. Ed.* **2015**, *54*, 5578-5582.
9. Bailey, P. J.; Coxall, R. A.; Dick, C. M.; Fabre, S.; Henderson, L. C.; Herber, C.; Liddle, S. T.; Loroño-González, D.; Parkin A.; Parsons, S. The first structural characterisation of a group

2 metal alkylperoxide complex: comments on the cleavage of dioxygen by magnesium alkyl complexes. *Chem. Eur. J.* **2003**, *9*, 4820-4828.

10. Mills, D. P.; Moro, F.; McMaster, J.; Van Slageren, J.; Lewis, W.; Blake, A. J.; Liddle, S. T. A delocalized arene-bridged diuranium single-molecule magnet. *Nat. Chem.* **2011**, *3*, 454-460.

11. Scott, B. L. *Actinide Research Quarterly*; LA-UR-15-24862; Los Alamos National Laboratory: 2015; pp 6-9.

12. Gregson, M.; Lu, E.; McMaster, J.; Lewis, W.; Blake, A. J.; Liddle, S. T. A cerium(IV)-carbon multiple bond. *Angew. Chem. Int. Ed.* **2013**, *52*, 13016-13019.

13. Mills, D. P.; Cooper, O. J.; Tuna, F.; McInnes, E. J. L.; Davies, E. S.; McMaster, J.; Moro, F.; Lewis, W.; Blake, A. J.; Liddle, S. T. Synthesis of a Uranium(VI)-Carbene: Reductive Formation of Uranyl(V)-Methanides, Oxidative Preparation of a $[R_2C=U=O]^{2+}$ Analogue of the $[O=U=O]^{2+}$ Uranyl Ion (R = $Ph_2PNSiMe_3$), and Comparison of the Nature of $U^{IV}=C$, $U^{V}=C$ and $U^{VI}=C$ Double Bonds. *J. Am. Chem. Soc.* **2012**, *134*, 10047-10054.

14. Bruker (2012). *SMART APEX II and SMART APEX III.* Bruker AXS Inc., Madison, Wisconsin, USA.

15. *CrysAlisPro 39.27b, Oxford Diffraction / Agilent Technologies UK Ltd., Yarnton, U.K.*, Oxford Diffraction / Agilent Technologies UK Ltd: 2017.

16. Dolomanov, O. V.; Bourhis, L. J.; Gildea, R. J.; Howard, J. A. K.; Puschmann, H., OLEX2: a complete structure solution, refinement and analysis program. *J. Appl. Crystallogr.* **2009**, *42*, 339-341.

17. Sheldrick, G. M. SHELXT - Integrated space-group and crystal-structure determination. *Acta Crystallogr. A* **2015**, *71*, 3-8.

18. Sheldrick, G. M. Crystal structure refinement with SHELXL. *Acta Crystallogr. C* **2015**, 71, 3-8.

19. Farugia, L. J. *J. Appl. Cryst.* **2012**, *45*, 849-854.

20. Persistence of Vision (TM) Raytracer, Persistence of Vision Pty. Ltd., Williamstown, Victoria, Australia

21. Clegg, W.; Blake, A. J.; Cole, J. M.; Evans, J. S. O.; Main, P.; Parsons, S.; Watkin, D. J. *Crystal Structure Analysis*. Oxford University Press, 2009.

22. Fonseca Guerra, C.; Snijders, J. G.; Te Velde, G.; Baerends, E. J. Towards an order-N DFT Method. *Theor. Chem. Acc.* **1998**, *99*, 391-403.

23. Te Velde, G.; Bickelhaupt, F. M.; Baerends, E. J.; Fonseca Guerra, C.; Van Gisbergen, S. J. A.; Snijders, J. G.; Ziegler, T. Chemistry with ADF. *J. Comput. Chem.* **2001**, *22*, 931-967.

24. Van Lenthe, E.; Baerends, E. J.; Snijders, J. G. Relativistic regular two-component Hamiltonians. *J. Chem. Phys.* **1993**, *99*, 4597-4610.

25. Van Lenthe, E.; Baerends, E. J.; Snijders, J. G. Relativistic total energy using regular approximations. *J. Chem. Phys.* **1994**, *101*, 9783-9792.

26. Van Lenthe, E.; Ehlers, A. E.; Baerends, E. J. Geometry optimization in the Zero Order Regular Approximation for relativistic effects. *J. Chem. Phys.* **1999**, *110*, 8943-8953.

27. Vosko, S. H.; Wilk, L.; Nusair, M. Accurate spin-dependent electron liquid correlation energies for local spin density calculations: a critical analysis. *Can. J. Phys.* **1980**, *58*, 1200-1211.

28. Becke, A. D. Density-functional exchange-energy approximation with correct asymptotic behaviour. *Phys. Rev. A.* **1988**, *38*, 3098-3100.

29. Perdew, J. P. Density-functional approximation for the correlation energy of the inhomogeneous electron gas. *Phys. Rev. B.* **1986**, *33*, 8822-8824.

30. Glendening, E. D.; Badenhoop, J. K.; Reed, A. E.; Carpenter, J. E.; Bohmann, J. A.; Morales, C. M.; Landis, C. R.; Weinhold, F. (Theoretical Chemistry Institute, University of Wisconsin, Madison, WI, 2013); http://nbo6.chem.wisc.edu/.

31. Bader, R. F. W. *Atoms in Molecules: A Quantum Theory*, Oxford University Press, New York, 1990.

32. Bader, R. F. W. A bond path: a universal indicator of bonded interactions. *J. Phys. Chem. A* **1998**, *102*, 7314-7323.

33. http://www.quimica.urv.es/XAIM.
